# Azobenzene's Cross‐Scale Optics and Photonics: Molecular Photoswitching, Mesoscopic Material Motions, and Adaptive Devices

**DOI:** 10.1002/adma.202522702

**Published:** 2026-08-05

**Authors:** Heeju Son, Soyul Kwak, Heerin Noh, Minsang Kim, Doyeon Noh, Subhayan Chakraborty, Jaewon Lee, YongDeok Cho, Kyeongsoo Kim, Taewoong Eom, Moonyoung Kim, Jangwon Kim, Hoyoung Lee, Soyoung Lee, Jaehun Kim, Grace G. D. Han, Shin‐Tson Wu, Stefano Oscurato, Arri Priimagi, Marina Saphiannikova, Seungwoo Lee

**Affiliations:** ^1^ KU‐KIST Graduate School of Converging Science and Technology Korea University Seoul South Korea; ^2^ Department of Biomicrosystem Technology Korea University Seoul South Korea; ^3^ Department of Chemistry and Biochemistry University of California Santa Barbara California USA; ^4^ Department of Integrative Energy Engineering (College of Engineering) Korea University Seoul South Korea; ^5^ College of Optics and Photonics University of Central Florida Orlando Florida USA; ^6^ Physics Department “E. Pancini” Complesso Universitario di Monte Sant'Angelo University of Naples “Federico II” Naples Italy; ^7^ Smart Photonic Materials Faculty of Engineering and Natural Sciences Tampere University Tampere Finland; ^8^ Division Theory of Polymers Leibniz Institute of Polymer Research Dresden Dresden Germany; ^9^ Faculty of Mechanical Science and Engineering Dresden University of Technology Dresden Germany; ^10^ Center for Opto‐Electronic Materials and Devices Post‐Silicon Semiconductor Institute Korea Institute of Science and Technology (KIST) Seoul South Korea

**Keywords:** azobenzene, directional photodeformation, liquid crystals, photoisomerization, photomechanics

## Abstract

Azobenzene is a widely studied molecular photoswitch that converts light absorption into reversible *E/Z* isomerization and, when embedded in soft or ordered media, into optical, mechanical, thermal, transport, and bioadaptive functions. This review examines azobenzene optics and photonics through a cross‐scale structure‐property‐function framework. We first summarize the mechanistic landscape of *trans–cis* isomerization, including π–π* and n–π* excitation, ultrafast relaxation pathways, and molecular design rules that tune absorption wavelength, quantum yield, photostationary state (PSS), and *cis‐*state lifetime. We then connect single‐molecule switching to collective responses in azobenzene‐containing materials, including photoalignment and all‐optical poling, stress‐driven surface patterning in amorphous polymers, photomechanics in liquid‐crystalline polymer networks (LCNs) and liquid crystal elastomers (LCEs), and phase‐transition‐based responses. On this basis, we organize applications according to their dominant device functions: information processing and reconfigurable photonics, dynamic liquid crystals (LCs) and adaptive optical devices, molecular solar thermal (MOST) energy storage, mechanical motion and soft robotics, mechanically enabled processing, bioadaptive transport, and opto/iontronic interfaces. The Review emphasizes quantitative links between molecular orientation, stress generation, and macroscopic deformation, and highlights how modeling and materials design can improve visible/red‐light operation, fatigue resistance, penetration depth, manufacturability, and device integration. We close by outlining challenges and opportunities for durable, scalable, and multifunctional azobenzene‐based adaptive photonic matter.

## Graphical abstract

Azobenzene photoswitches translate molecular‐scale *E*/*Z* photoisomerization into macroscopic material responses and device‐level photonic functions. This Review highlights how azobenzene research has evolved from molecular photochemistry to photoalignment, mass migration, photomechanics, and heat release, ultimately enabling holography, reconfigurable optics, metasurfaces, optical information storage, and solar–thermal energy systems across multiple length scales.

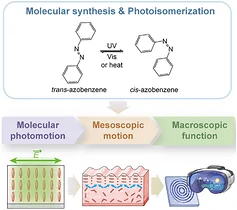

## 1 Introduction

Azobenzene is a widely studied molecular photoswitch whose significance extends beyond a single reversible photochemical reaction [1, 2]. Its nearly two‐century history links organic synthesis, molecular photochemistry, liquid crystal (LC) science, polymer physics, soft matter, energy storage, and modern photonics (Figure 1) [1, 3, 4, 5]. First described by Mitscherlich in 1834 [6] and later prepared on a larger scale by Noble in 1856 [7, 8], azobenzene became a central photoswitch after Hartley identified the *cis‐*form and reversible light‐induced isomerization in 1937 [9, 10]. Since then, azobenzene research has progressed not only chronologically but also across length scales [1, 3, 11, 12].

**FIGURE 1:**
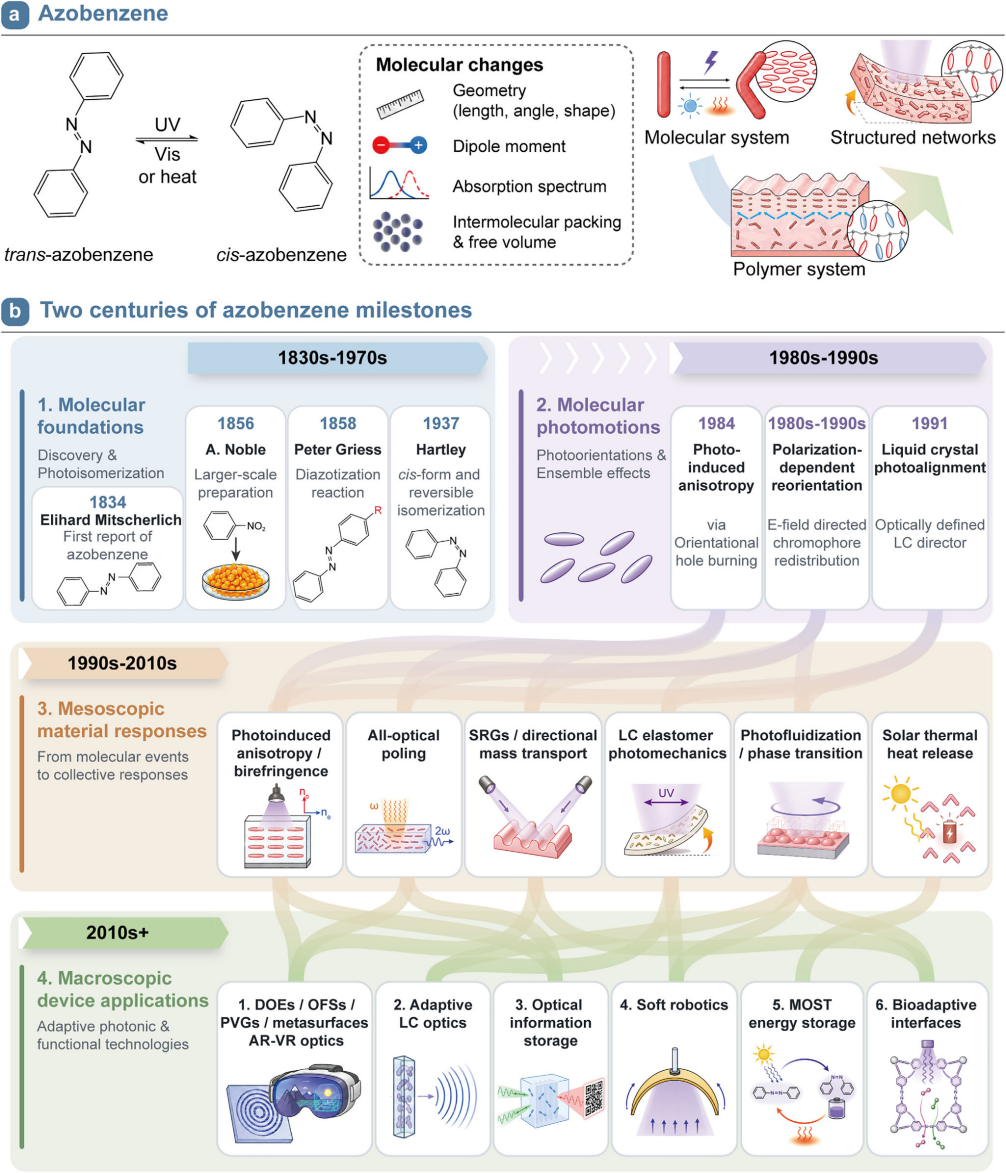
Two centuries of azobenzene research: major milestones from molecular discovery to adaptive photonics. (a) Molecular photoswitching of azobenzene. Reversible *trans–cis* (or *E*/*Z*) photoisomerization changes molecular geometry, dipole moment, absorption spectrum, intermolecular packing and free volume of azobenzene, allowing a nanoscale photochemical event to perturb surrounding molecular, polymer, and network environments. (b) Historical and cross‐scale evolution of azobenzene research. The schematic highlights selected milestones in azobenzene chemistry, photoisomerization, polymer and liquid crystal (LC) materials, photomechanics, molecular solar thermal (MOST) energy storage, and emerging reconfigurable photonic platforms. This timeline is intended as an orienting map rather than an exhaustive historical list.

In particular, azobenzene consists of two phenyl rings linked by an N═N bond (Figure 1a), and its two isomers have distinct structures and properties [13]. In the thermodynamically stable *trans*, or *E*, isomer, the aromatic rings are positioned on opposite sides of the azo bond, giving a nearly planar structure with a negligible dipole moment [14, 15, 16]. In the metastable *cis*, or *Z*, isomer, the phenyl rings lie on the same side of the N═N bond, producing a bent, nonplanar structure with a sizable dipole moment [2, 16, 17]. The *trans* isomer is more stable by roughly 50 kJ mol^−1^, and the ground‐state isomerization barrier is on the order of 100 kJ mol^−1^ [13, 18]. Consequently, *trans‐*azobenzene is stable at room temperature, whereas the *cis* isomer thermally relaxes back to the *trans* state in the dark over a timescale determined by molecular structure and environment [2, 13]. Light absorption can reversibly shift this equilibrium, providing the molecular basis for azobenzene's function as a photoswitch [2, 13, 19].

The interconversion between *trans* and *cis* azobenzene lies at the heart of its interaction with light [1, 2]. *Trans‐*azobenzene strongly absorbs in the ultraviolet (UV) region through a π–π* transition and can isomerize to the *cis* form upon UV illumination [2, 19]. The *cis* isomer can be converted back to *trans* either thermally or by visible‐light irradiation through excitation of the n–π* band [2, 20]. Alternating UV and visible light therefore allows repeated switching between two molecular states with different geometry, dipole moment, absorption spectrum, free volume, and intermolecular packing [2, 3, 4]. These molecular changes are small in absolute length scale, but they are highly effective in perturbing their surrounding environment [11, 13]. In molecular or supramolecular systems, they modulate conformation, polarity, and host‐guest interactions [13, 21, 22]. In polymers and LCs, they generate orientational redistribution, stress, free‐volume changes, and phase transitions [3, 23, 24, 25, 26, 27]. In structured films and networks, they can be amplified into mass transport, deformation, birefringence, surface patterning, or heat release [3, 11, 28, 29, 30, 31]. This ability to convert a nanoscale photochemical event into mesoscale and macroscopic material responses is the central feature that distinguishes azobenzene from many other photochromic molecules [11, 12].

As summarized in Figure 1b, the field first developed around molecular‐scale fundamentals, then expanded into cross‐scale material responses, and has more recently differentiated toward device‐ and system‐level photonic functions [1, 3, 32, 33, 34, 35, 36]. This cross‐scale evolution provides an important perspective through which to understand the present state of azobenzene materials’ research [1, 3, 11, 12]. Until roughly the 1970s, the field was dominated by molecular‐scale questions: molecular structure, synthesis, UV/visible (UV/vis) absorption, π–π* and n–π* transitions, *E/Z* isomer assignment, thermal relaxation, quantum yields, and excited‐state isomerization pathways [6, 7, 8, 9, 10, 14, 18, 37, 38]. These studies established azobenzene as a chemically tunable, reversible light‐responsive molecule [2, 4]. In the 1980s and early 1990s, research began to move beyond isolated molecular photochemistry toward ensemble‐level molecular photomotions, particularly photoinduced anisotropy, orientational hole burning, and polarization‐dependent reorientation in azobenzene‐containing polymers and LC systems [3, 31, 39, 40, 41, 42, 43]. A major differentiation then occurred from the 1990s to the 2010s: repeated molecular *E/Z* photoisomerization was increasingly understood as a driving force for mesoscopic and macroscopic material responses [12, 28, 29, 44, 45, 46]. During this period, azobenzene photoresponses were linked to photoalignment and birefringence [3, 31, 39, 40], all‐optical poling [47, 48, 49, 50, 51], surface relief grating (SRG) formation and directional mass migration [12, 28, 29, 52], liquid crystal elastomer (LCE)/liquid‐crystalline polymer network (LCN) photomechanics [24, 25, 44, 45], photofluidization or photosoftening [46, 53], phase transitions [23, 54, 55], and molecular solar thermal (MOST) heat release [5, 30, 56]. Since the 2010s, these material‐level properties have been further translated into device‐level functions, including diffractive optical elements (DOEs) [32, 57], optical Fourier surfaces (OFSs) [32, 57, 58], geometric‐phase [35, 59, 60] and polarization‐volume gratings (PVGs) [34, 61, 62], augmented‐reality (AR)/virtual‐reality (VR) relevant LC optics [63, 64], reconfigurable metasurfaces [36, 65, 66], optical information storage [33, 67], soft robotic actuators [68, 69, 70], MOST energy storage systems [5, 30, 56], and photoadaptive biointerfaces [19, 71, 72]. Thus, the history of azobenzene research can be viewed as a progression from “molecular photoswitching”, to “material architecture and macroscopic response”, and finally to “device function”.

Our review is organized around this molecular‐to‐device amplification pathway. We focus on how molecular *E/Z* photoswitching propagates upward across length scales: molecular design controls absorption wavelength, quantum yield, photostationary state (PSS), thermal half‐life, fatigue resistance, dipole change, and shape change; these intrinsic switching metrics determine collective material responses such as photoalignment, birefringence, all‐optical poling, stress generation, mass migration, phase transition, photomechanical deformation, and MOST‐related heat release; and these material responses enable device functions including diffractive optics, geometric‐phase optics, reconfigurable LC devices, OFSs, metasurfaces, information storage, soft actuation, solar thermal energy storage, biointerfaces, and molecular/optoelectronic systems. In this framework, azobenzene is not treated merely as a molecular photoswitch or as a component of individual application platforms, but as a cross‐scale material transducer that links photochemistry to structure, mechanics, optics, and device performance.

This perspective differentiates the present Review from prior accounts that have mainly focused on molecular photophysics [2, 18], azopolymers [3], LCs [40, 73, 74, 75], photomechanics [11, 24, 25], MOST [5, 76, 77], or photopharmacology [19, 71, 78] as separate topics. Here, the unifying theme is the quantitative **structure–property–function** relationship that connects molecular design, molecular orientation, stress generation, material deformation, optical modulation, and device‐level performance. This organization also addresses a practical design question: for a target photonic or energy function, which molecular tuning parameters, host environments, and material architectures should be prioritized? We first examine the molecular photoisomerization mechanism, spectroscopic signatures, and molecular engineering principles of azobenzene. We then discuss how these molecular events generate collective responses in polymers, LCs, networks, and condensed‐phase materials. Finally, we survey device‐level applications by emphasizing how the underlying material responses are translated into optical, mechanical, energy, electronic, and biological functions.

## 2 Fundamental Photoresponse

### 2.1 Molecular Photoisomerization Mechanism and Spectroscopic Characteristics

#### 2.1.1 Electronic Transitions and Isomerization Pathways

Azobenzene's photoisomerization (Figure 2a) is initiated by light absorption, exciting the molecule from its ground state to an electronically excited state (Figure 2b) [2, 18, 79]. In *trans*‐azobenzene, the strongest absorption is in the UV (a wavelength range of 320–350 nm), corresponding to a π→π* transition (S_2_ state), while a weaker n→π* band appears in the violet/blue (∼450 nm wavelength) range (S_1_ state) [2, 18, 19, 79]. Hence, UV light is used to drive the *trans–cis* conversion, whereas the *cis–trans* back‐conversion can be triggered by blue light [2, 18]. In the absence of light, *cis*‐isomer will thermally relax to *trans*‐isomer over time (hours to days at room temperature for unsubstituted azobenzene) [10, 18, 20]. The distinct absorption bands of the *trans‐* and *cis‐*forms give azobenzene its classic photochromism: *trans* is usually colorless or light yellow (due to the weak n–π* band in the visible), while *cis* has a shifted spectrum and can impart a different color; for example, a *para*–methoxyazobenzene changes from orange (*trans*) to yellowish (*cis*) upon UV irradiation and reverses under visible light.

**FIGURE 2:**
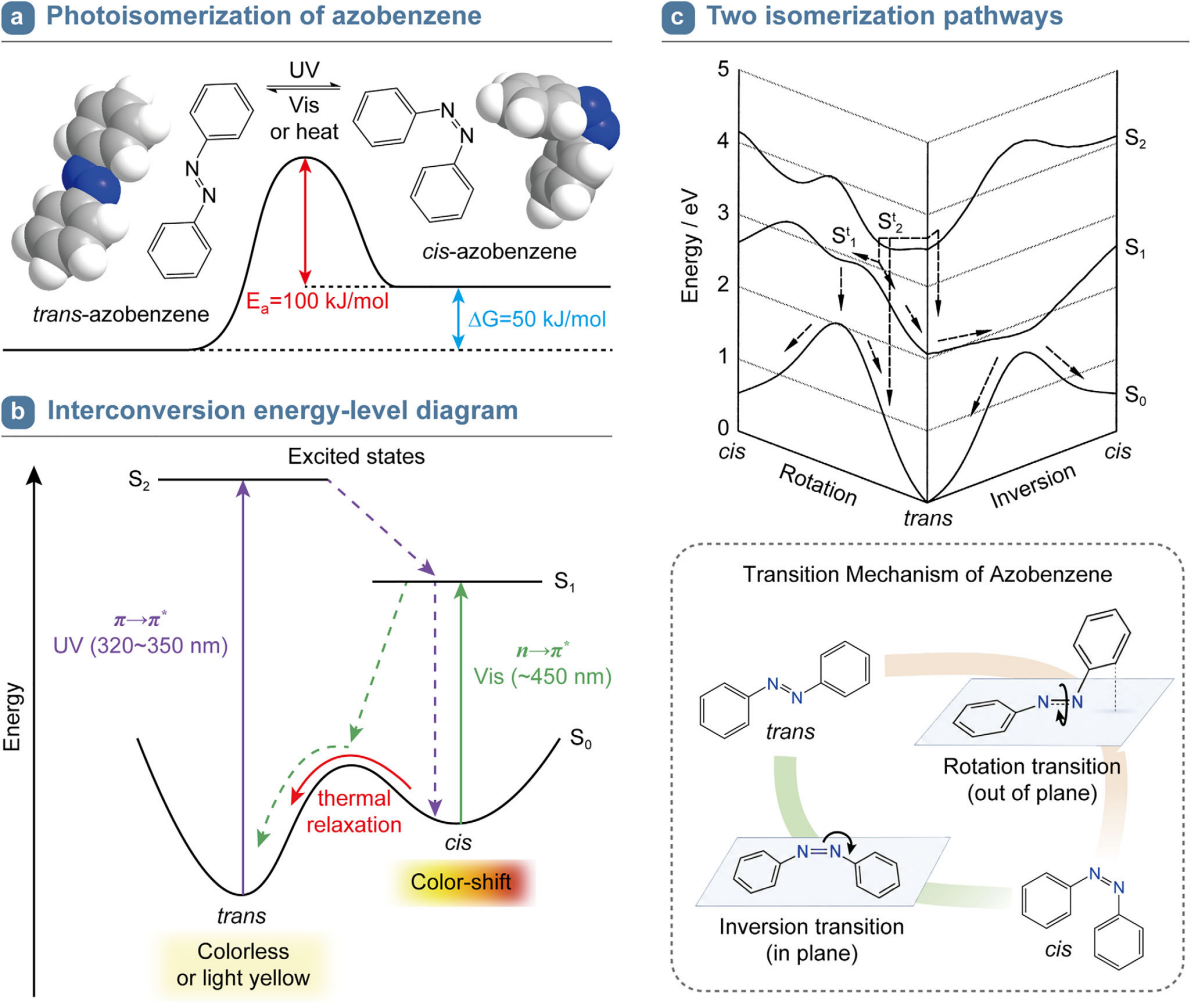
Schematic of azobenzene photoisomerization. (a) Molecular structures illustrating the reversible *trans–cis* photoisomerization of azobenzene and (b) interconversion energy‐level diagram for photoisomerization: ultraviolet (UV) light (typically 320–350 nm wavelength) excites the π–π* transition (from ground state (S_0_ state) of *trans* to S_2_ state) of the *trans* isomer, inducing a conformational change to the *cis* isomer. The *cis* form can be converted back to *trans* by visible light (∼450 nm wavelength, exciting the n–π* band (from S_0_ state of *cis* to S_1_ state)) or by thermal relaxation in the dark. *Trans*‐azobenzene is planar and more stable, while *cis*‐azobenzene is nonplanar and stores the absorbed photon energy (about 50 kJ mol^−1^ higher in energy). (c) Potential energy curves of azobenzene (S_0_, S_1_, and S_2_) and representative possible decay pathways along the molecular transition mechanism. Reproduced with permission [80]. Copyright 1996, American Chemical Society.

On the molecular scale, the mechanism by which the N═N bond converts from *trans* to *cis* (and vice versa) has been a topic of enduring research and debate [37, 81]. Two primary isomerization pathways were long considered (Figure 2c): [80] a rotation about the N═N bond (breaking the π‐bond at excitation and bending 180°) or an inversion mechanism, where one –N–center in the excited state becomes pyramidal (sp^3^‐like) and the molecule inverts through a semi‐linear transition state [37, 81, 82]. It is now understood that the reality is more complex, and multiple pathways and electronic states can participate depending on experimental conditions and environment. The prevailing view is that *trans–cis* photoisomerization predominantly occurs via an out‐of‐plane rotation on the excited potential energy surface, whereas the *cis*–*trans* may proceed via a different route, possibly an inversion on the n–π* state [81, 83, 84, 85]. Femtosecond (fs) transient absorption studies have shown that after UV excitation of *trans* (π–π*), the molecule quickly undergoes internal conversion from the S_2_ (π–π*) state to the S_1_ (n–π*) state, and then isomerizes towards *cis* [80, 86]. This suggests a picture where the initially accessed excited state relaxes to a lower state that guides the reaction coordinate.

In 2004, Diau proposed a “concerted inversion” mechanism in which both C─N═N bond angles bend simultaneously in a concerted fashion [87]. More recent ultrafast spectroscopy and computational work continue to refine this understanding. For instance, a 2022 study [88] provided direct evidence for a multi‐state pathway, even involving triplet states under certain conditions, to resolve an entropy‐related puzzle in azobenzene isomerization kinetics. In summary, azobenzene's isomerization is a fast, picosecond (ps) scale photochemical reaction with a rich mechanistic landscape involving conical intersections and possibly multiple excited‐state pathways before the molecule settles into the new isomer [81, 83, 84, 86, 88].

Leveraging these mechanistic insights, Klajn et al. recently reported a supramolecular strategy, disequilibration by sensitization under confinement (DESC), enabling *trans*–*cis* switching using low‐energy (green to red) light [89]. In this approach, a water‐soluble coordination cage co‐encapsulates a *trans*‐azobenzene and a triplet‐sensitizer (e.g., BODIPY). Upon irradiation, triplet energy transfer from the sensitizer to azobenzene occurs inside the confined cavity, eventually converting the azobenzene to the* cis *isomer and expelling it from the cavity. This leaves the cage free to process additional substrate, thus operating catalytically. The approach is applicable to a wide range of azobenzene derivatives and sensitizers, enables selective switching in mixtures, and functions in both aqueous and organic media. DESC therefore overcomes the long‐standing limitation of azobenzene chemistry, the need for UV light for isomerization, opening new possibilities for light‐controlled processes in biological settings and soft materials [19, 71, 90, 91].

Because DESC relies on co‐encapsulation and triplet sensitization, its current applicability is narrower than that of bulk, unconfined systems. Nevertheless, it offers a viable route to visible‐light operation under designed environments [89, 92]. The practical outcome is clear: *trans* and *cis* forms can be reliably interconverted with light of appropriate wavelength, making azobenzene a robust molecular switch.

#### 2.1.2 Spectroscopic and Structural Signatures

The *trans–cis* isomerization is accompanied by significant changes in molecular geometry and electronic structure, which are reflected in spectroscopic signatures [2, 93, 94]. In the *trans* form, azobenzene is roughly planar and conjugated, fostering a delocalized *π* system [14, 15, 94, 95]. By contrast, *cis*‐azobenzene is nonplanar and less conjugated, contributing to its ground‐state (thermodynamic) energy and different absorption spectrum [2, 17, 79, 93, 94]. Unsubstituted *trans*‐azobenzene has no net dipole moment, whereas *cis*‐azobenzene has a sizable dipole (∼3 D) due to the bent configuration [13, 16]. These differences can be probed by UV/vis (Figure 3a) [2, 38, 79, 94], infrared (IR) and Raman spectroscopy, the latter showing changes in N═N and C–N stretches depending on the isomer (Figure 3b) [93, 94, 96, 97, 98]. Representative IR/Raman signatures for *trans*‐azobenzene include an N═N stretching band near 1440 cm^−1^ with characteristic coupling to ring modes; isotope substitution confirms the assignment and coupling pattern [94, 96, 98].

**FIGURE 3:**
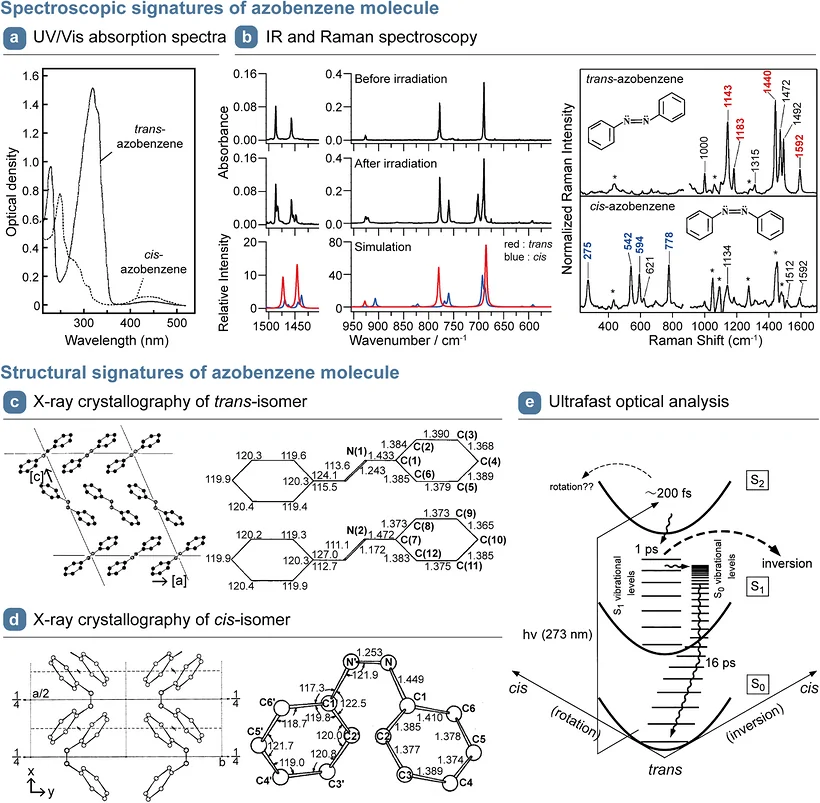
Spectroscopic and structural signatures of the azobenzene molecule accompanied by molecular geometry: (a) UV/visible (UV/vis) absorption spectra of *trans*‐ and *cis*‐azobenzene. Adapted with permission [38]. Copyright 1962, American Chemical Society. (b) Infrared (IR) spectra of pure *trans*‐ (before irradiation) and *cis*‐azobenzene (after irradiation) isolated in argon matrices at 15 K. Adapted with permission [94]. Copyright 2014, PCCP Owner Societies. Raman spectra of *trans*‐ and *cis*‐azobenzene in ethanol with 406 nm excitation; prominent peaks are marked in red (*trans*) and blue (*cis*). Adapted with permission [93]. Copyright 2007, American Chemical Society. (c) Diagram of unit cell for crystalline *trans‐*azobenzene with selected bond lengths and inter‐bond angles from single‐crystal x‐ray crystallography. Adapted with permission [14]. Copyright 1966, International Union of Crystallography. (d) Crystal structure of *cis*‐azobenzene with corresponding bond metrics from x‐ray crystallography of *cis*‐azobenzene. Adapted with permission [17]. Copyright 1971, Acta Chemica Scandinavica. (e) Relaxation pathways following S_2_←S_0_ photoexcitation of pure *trans‐*azobenzene in hexane. Reproduced with permission [97]. Copyright 2000, American Chemical Society.

X‐ray crystallography and other methods have provided structural confirmation, revealing the *trans* isomer's N═N bond length of ∼1.19 Å and planarity (Figure 3c), whereas *cis*‐azobenzene has a longer N═N bond length (∼1.25 Å) and a dihedral angle of ∼173.5° between the phenyl rings (Figure 3d) [14, 15, 17, 99, 100, 101, 102]. These data confirm that the *cis* isomer is significantly strained and less *π*‐delocalized, rationalizing its ∼50 kJ mol^−1^ destabilization. In gas‐phase electron diffraction, the central N═N bond length in *trans*‐azobenzene is on the order of 1.25–1.26 Å and the C─N bonds ≈1.42–1.43 Å; ring torsions are minimal, consistent with an essentially planar *trans* backbone [99, 101, 102]. Thermal *cis–trans* relaxation barriers determined under minimally perturbing conditions are ∼1.09 ± 0.09 eV (measured in azobenzenes encapsulated in metal‐organic frameworks) and ∼1.17 ± 0.20 eV (measured in argon atmosphere), consistent with an inversion‐dominated pathway in many derivatives [20, 103]. Ultrafast optical studies resolve S_2_ (π–π*) excitation feeding S_1_ (n–π*) surfaces before ground‐state recovery and isomerization [80, 83, 86, 104]; fs fluorescence and ps time‐resolved Raman spectroscopy track the sub‐ps to ps evolution of key coordinates (Figure 3e) [97, 105, 106].

#### 2.1.3 Mechanism‐Driven Molecular Engineering

The photochemical behavior of azobenzene derivatives is best understood not as a trial‐and‐error classification, but as a set of design levers that perturb orbital energies, excited‐state ordering, steric constraints, and ground‐state stability (Figure 4) [1, 2, 92, 107]. Historically, three families have been used to describe mainly *para*‐substituted azobenzenes: [2] azobenzene‐type systems (Figure 4a), aminoazobenzenes (Figure 4b), and *pseudo*‐stilbene or push‐pull systems (Figure 4c). This terminology remains useful for orientation, but it is incomplete unless the underlying rationale is stated [2]. To move the operating wavelength from the UV toward the visible, one must reduce the relevant electronic energy gaps and/or separate the *E*‐ and *Z*‐isomer absorption bands. *Para* donor‐acceptor substitution increases charge‐transfer character, red‐shifts the π–π* transition, and can enable visible‐light excitation; however, the same electronic perturbation often accelerates thermal *Z*‐to‐*E* relaxation and can reduce bistability [2, 108, 109]. Thus, push‐pull substitution is advantageous for fast optical modulation, photoalignment, or mass‐transport systems in which repeated cycling is desired [12, 110, 111, 112], but it is less suitable when a long‐lived metastable *Z* state is required, such as in MOST or photopharmacology [5, 71, 76, 77, 78].

**FIGURE 4:**
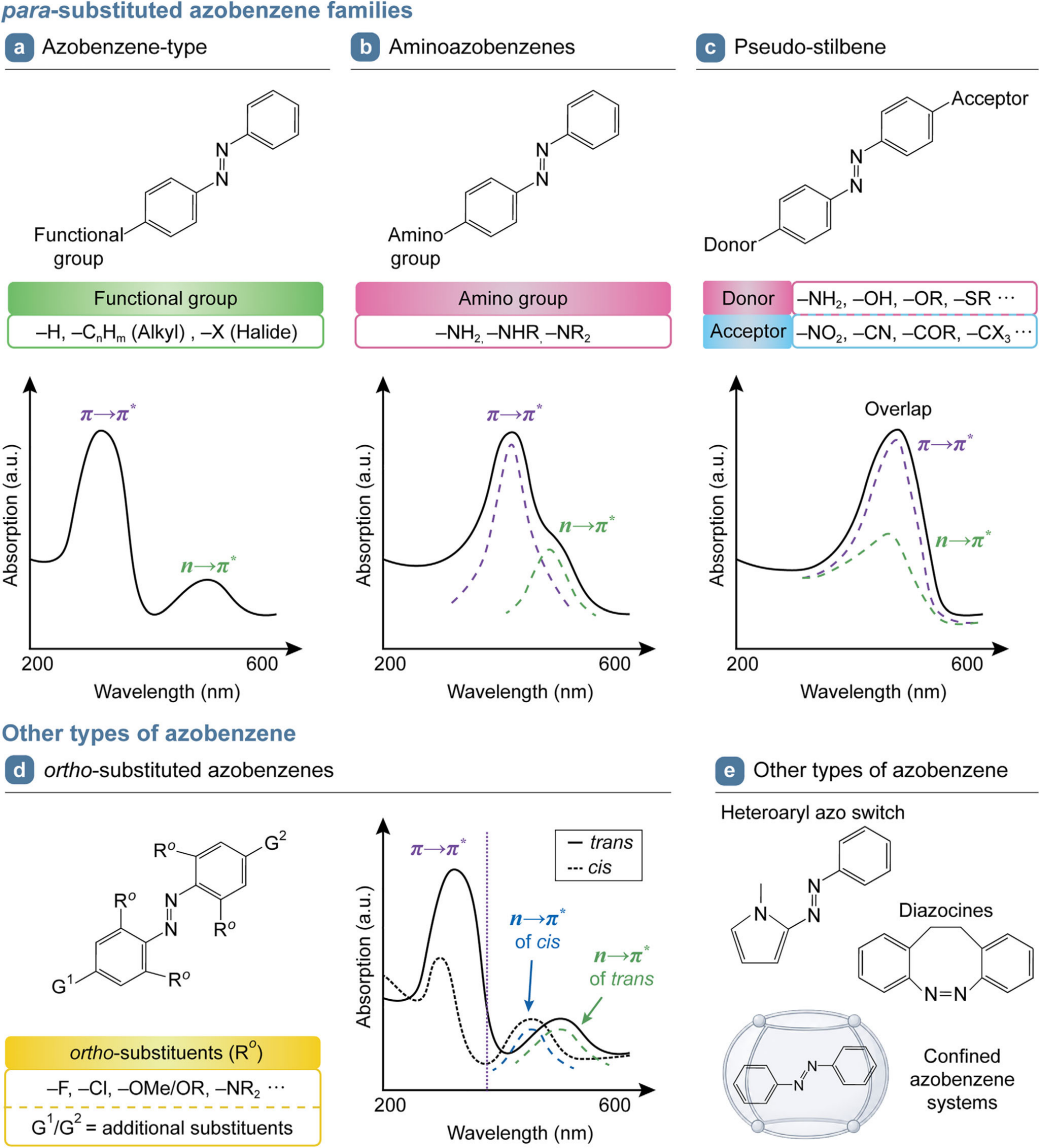
Schematics of tunable UV/vis spectra of azobenzene as a function of substitution. (a) “Azobenzene‐type” (unsubstituted or simple substituents) which resemble parent azobenzene with a strong UV π–π* band and a weak visible n–π* band with minimal overlap; (b) “Aminoazobenzenes” (*para*‐ or *ortho*‐substituted with electron donors like –NR_2_) which exhibit both π–π* and n–π* bands in the visible range and generally have faster thermal isomerization; and (c) “Pseudo‐stilbene” type, with push–pull substitution (*para*‐donor and acceptor), in which both π–π* and n–π* bands overlap, allowing a single visible wavelength to drive bidirectional switching. (d) “*ortho*‐substituted azobenzene” types exhibit separated n–π* band for the *trans* and *cis* isomers due to steric/electronic tuning, enabling efficient, wavelength‐selective visible‐light control of photoisomerization and broad tuning of the *cis*‐state half‐life. (e) Architecture‐ and environment‐driven azo photoswitch design. Heteroaryl, diazocines, and confined azobenzene systems expand photoswitching control by tuning excited‐state pathways, isomer stability, photostationary state (PSS) contrast, and low‐energy sensitized switching.

*Ortho*‐substitution (Figure 4d) provides a complementary, more wavelength‐selective design strategy. *Ortho*‐fluoro, *ortho*‐chloro, *ortho*‐methoxy, and related substitutions can shift and split the n–π* transitions of *E* and *Z* isomers through a combination of inductive effects, steric twisting, and lone‐pair energy modulation [1, 2, 113, 114, 115, 116, 117, 118]. This separation enables bidirectional visible‐light switching. This is because one wavelength preferentially addresses the *E* isomer while another addresses the *Z* isomer [113, 114, 115, 116, 117]. In many *ortho*‐halogenated azobenzenes, electron‐withdrawing substituents lower the energy of nonbonding orbitals and increase the *Z*‐isomer thermal half‐life, allowing visible‐light operation without sacrificing storage stability [114, 117, 118]. By contrast, *ortho*‐alkoxy or *ortho*‐amino designs can red‐shift switching strongly but may shorten half‐life depending on hydrogen bonding, steric strain, and solvent environment [113, 119, 120]. Heteroaryl azo switches, diazocines, BF_2_‐coordinated azo compounds, macrocyclic azobenzenes, and host‐guest/confined systems add further levers (Figure 4e): they can reshape excited‐state pathways, stabilize a chosen isomer, increase PSS contrast, or enable sensitized low‐energy switching [89, 107, 109, 121, 122, 123, 124, 125, 126, 127, 128, 129]. As such, we use Figure 4 as a design map rather than a rigid taxonomy: molecular electronics and sterics set absorption and kinetic parameters, while environment and architecture determine whether those parameters translate into optical memory, photomechanics, energy storage, or biological control.

### 2.2 Photophysical Performance

The efficiency of azobenzene's photoisomerization is quantified by its quantum yields, which are typically quite high, making it an efficient photoswitch. In solution at room temperature, *trans*–*cis* quantum yields are in the range of 0.1–0.2 under π–π* excitation, i.e., 10%–20% of absorbed UV photons lead to isomerization [2, 38, 130, 131, 132, 133, 134]. *cis*–*trans* quantum yields under n–π* visible excitation are higher, 0.33–0.60, reflecting the different excitation bands rather than the reaction direction [2, 131, 133, 134]. Some specialized azobenzenes (e.g. rotation‐restricted or band‐engineered systems) reach π–π* excitation quantum yields of > 0.5 [2, 87, 108, 114, 115, 117, 118, 134, 135, 136, 137, 138, 139], and under idealized low‐collision or tailored ultrafast conditions, for example hole‐catalytic systems or strong coupling, band‐ and direction‐specific apparent yields close to unity have been reported, indicating near‐deterministic isomerization in those conditions [140, 141]. By contrast, in constrained or densely packed solids (e.g. single crystals or tightly packed films), the attainable conversion can decrease despite longer *cis* thermal lifetimes, due to steric/packing limits and polarization/alignment effects [142, 143, 144, 145, 146, 147, 148, 149, 150]. Because these values vary strongly with excitation band/wavelength, solvent polarity/viscosity, temperature, protonation, substitution pattern, and confinement, the reported values of quantum yield for a given azobenzene scaffold can change widely with conditions, and outliers do occur [2, 114, 130, 134, 135, 138, 142, 143, 144, 145, 146, 147, 148, 149, 150].

The actual reconfiguration process, i.e., the structural rearrangement from *cis* to *trans* or vice versa, occurs on an ultrafast timescale, typically within a few ps after photon absorption [104, 105, 151, 152, 153, 154, 155]. In contrast, the half‐life of the *cis* state, representing how long the metastable form persists before thermally reverting to *trans*, can span from microseconds (µs) to years depending on molecular structure and environment [22, 112, 117, 121, 135, 154, 156]. Therefore, it is important to distinguish between the intrinsic photoswitching speed (ps‐timescale) and the thermal lifetime of the metastable state. The wavelength and intensity of illumination define how frequently *trans* molecules undergo switching cycles, effectively setting the optical “clock speed” of azobenzene photoswitching. The photophysical performance of different classes of azobenzene derivatives is summarized in Table 1. This wide range of timescales from ultrafast photochemistry to slow thermal relaxation is another aspect that makes azobenzene useful: one can trigger a change quickly and then have a metastable state persist until deliberately reversed.

**TABLE 1:** Photophysical performance of azobenzene molecules (quantum yields).

Conditions	State^a^	λ_*Excitation*_ ^b^ (nm)	$\phi_{T\to C}$ ^c^	$\phi_{C\to T}$ ^d^	τ_*photo*_ ^e^	$t_{thermal}^{1/2}$ ^f^	Additional information	Refs.
**I. Unsubstituted azobenzene**								
Benchmark	Solution	UV (π–π*) 280–334 & vis (n–π*) 405–436	0.12–0.15 (π–π*), 0.23–0.32 (n–π*)	0.33–0.60	0.1–100 ps	∼2 days (methanol @ 20°C.: ∼ 11.1 days)	Strong wavelength/band dependence; sensitive to solvent and T; acids accelerate thermal transition	[2, 130, 131, 132, 133, 134]
**II. Substituted azobenzenes**								
Amino‐azobenzene type	Solution	UV & vis	0.17–0.36 (π–π*), 0.25–0.84 (n–π*)	0.40–0.68	sub‐ps–100 ps	∼3.8 h	Strong band dependence; highly sensitive to solvent and substitute type	[2, 87, 120]
Pseudostilbene type (Push‐pull)	Solution	Vis (400–650 nm)	0.0013–0.19	Highly variable	sub‐ps–1.5 ps	ms–tens of seconds	Strong wavelength/solvent dependence; visible‐band overlap; rotation‐dominated	[108, 134, 136, 137, 139]
Vis‐only switching azobenzene (*ortho*‐halogenation type)	Solution	Vis (green‐blue)	∼0.23	∼0.51	sub‐ps–100 ps	∼700 days	Bidirectional visible‐only switching; long‐lived *cis* isomer (derivative/solvent dependent)	[114, 117, 118]
Hydroxy‐azobenzene type	Solution	UV & vis	Highly variable	Highly variable	sub‐ps–100 ps	6 ms–30 min	Intramolecular H‐bond/ESIPT^*^ → fast thermal back; strong solvent (polarity)/pH sensitivity.	[135, 138]
**III. Constrained or rigid environments**								
Channel/porous inclusion hosts	Inclusion crystal /host‐guest	UV & vis	0.07–0.10	Variable	sub‐ps–100 ps	Variable	Host size/occupancy dependence; Polarization dependence	[142, 143, 147]
Framework‐linked & network‐anchored	Crystalline framework (MOF^**^/COF^***^)	UV & Vis	<<0.1	Often not reported	sub‐ps–100 ps	ms–days	Framework/solvent‐of‐crystallization dependence	[146, 148, 149]
Ordered packings (neat/co‐crystal & 2D monolayer)	Molecular crystal / ordered monolayer	UV & vis	Variable	Variable	sub‐ps–100 ps	ms–weeks	Polarization/packing‐motif dependence, defects/strain dependence	[144, 145, 150]
**IV. Gas phase / ultrafast excitation / high‐efficiency schemes**								
Jet‐cooled gas phase (one‐photon)	Gas, jet‐cooled	UV (π–π*) 300–350 & Vis (n–π*) 430–470	Variable	—	sub‐ps–few‐ps	—	Strong wavelength/band dependence	[2, 81, 83, 155]
Ultrafast excitation in solution	Solution	UV (π–π*) 313–334 & Vis (n–π*) 440–460	0.20–0.27	0.09–0.12	sub‐ps–few‐ps	∼200 h (20°C, *n*‐hexane)	Strong wavelength/band/solvent dependence	[81, 83, 87, 105, 134, 154]
Indirect/catalytic high‐efficiency schemes	Solution or condensed/solid	(i) Hole‐catalysis (ii) strong coupling	Up to >1(up to ∼2)	—	fs–ps (optical), ms–s (electrical)	Variable	Molecular structure/solvent dependence, chain photoisomerization	[140, 141]

^a^ In most cases, temperatures are reported at room temperature (25°C).

^b^ Excitation wavelength used in references.

^c^ Quantum yield for *trans*–to–*cis* photoisomerization: fraction of absorbed photons that produce *cis* isomerization.

^d^ Quantum yield for *cis*–to–*trans* photoisomerization: fraction of absorbed photons that produce *trans* isomerization.

^e^ Photochemical timescale: time from photoexcitation to completion of the primary photochemical step (isomerization/internal conversion) in ultrafast measurements.

^f^ Thermal half‐life time: time for half of the *cis* population to thermally revert to *trans* in the dark (strongly temperature/solvent dependent).

* ESIPT: Excited‐state intramolecular proton transfer.

** MOF: Metal–organic framework.

*** COF: Covalent‐organic framework.

In summary, at the molecular level, azobenzene provides a reversible two‐state photoswitch whose usefulness arises from tunable absorption bands, PSS, quantum yields, shape changes, dipole changes, and thermal lifetimes [2]. These parameters should not be optimized independently: visible‐light absorption, high PSS conversion, long *Z*‐state lifetime, high quantum yield, and fatigue resistance often impose competing molecular‐design requirements [1, 2, 92, 114, 117, 145]. The key task is therefore to choose a molecular design and environment that match the desired material function. The following sections examine how these molecular parameters are amplified through polymers, LCs, networks, supramolecular assemblies, and condensed phases to generate optical anisotropy, stress, mass transport, deformation, and heat release.

### 2.3 Photoinduced Motions in Azobenzene‐Containing Materials

Beyond geometric switching, repeated photoisomerization cycles can drive orientational selection of azobenzene chromophores relative to the polarization direction of incident light. Because this process originates from individual molecular photoresponses, it can occur even in amorphous materials without requiring cooperative ordering. When azobenzenes are embedded in ordered or strongly interacting hosts, including LCs, LCEs/LCNs, semicrystalline assemblies, or glassy polymers, these molecular events become coupled to elastic, viscous, or plastic responses [3, 11, 24, 25, 45]. The resulting photoinduced motions span nanometers to millimeters and include photoorientation, birefringence, directional plastic deformation, surface‐relief formation, and macroscopic 3D motion [3, 12, 25, 29, 69, 70, 157].

#### 2.3.1 Polarized‐Light‐Induced Alignment and Anisotropy

Rather than being a truly cooperative phenomenon, the light‐induced molecular alignment observed in early azobenzene‐containing material studies arises from independent photoalignment of individual azobenzene molecules within amorphous matrices [3, 41, 42]. The macroscopic birefringence and dichroism that result merely reflect the ensemble average of these single‐molecule reorientations [3]. In 1920, Weigert reported that linearly polarized light could induce dichroism in azobenzene‐containing films [43], an observation later explained as angular‐selective photoisomerization or “orientational hole‐burning” [31, 41, 42, 158]. The mechanism is as follows: when a polymer doped with azobenzene is illuminated with linearly polarized light, those azobenzenes whose transition dipole moments are aligned with light polarization absorb preferentially and undergo *trans–cis* isomerization, as shown in Figure 5a [3, 40, 41, 42, 158, 159]. After a few *trans–cis–trans* cycles (*cis* relaxing back to *trans* either thermally or by light irradiation), most azobenzenes whose transition moments are initially parallel to the polarization become depleted, while those oriented perpendicularly are less prone to light absorption [41, 42, 158]. Thermal relaxation of the *cis* isomers can, in principle, randomize orientation; however, this effect is generally negligible under continuous illumination and becomes noticeable only at very low light intensities, where the photochemical cycling is comparable to the thermal back‐isomerization rate [3, 160, 161, 162]. This angular‐selective photoisomerization process effectively creates an orientational “hole” in the polarization direction and an accumulation of *trans* azobenzenes oriented orthogonally, resulting in a net anisotropic distribution known as photoalignment or orientational hole‐burning. This phenomenon gives rise to macroscopic dichroism and birefringence in the azopolymer film [31, 163, 164, 165]. Linearly polarized light breaks the in‐plane isotropy, driving azobenzene transition dipoles to orient predominantly perpendicular to the electric‐field vector (Weigert effect) [42, 43, 158].

**FIGURE 5:**
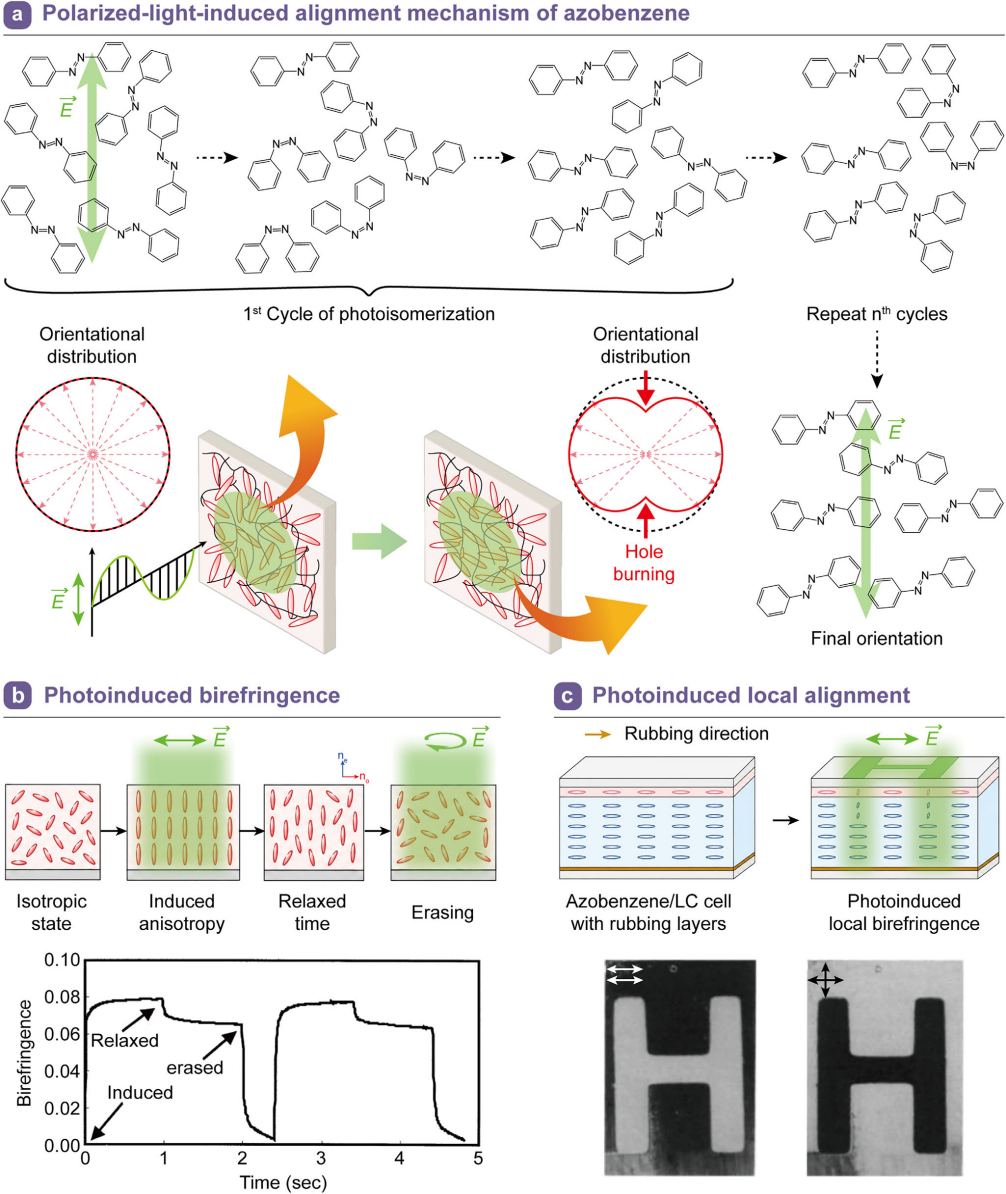
Polarized‐light‐induced photoorientation and anisotropy in azobenzene materials. (a) Orientational hole‐burning mechanism: repeatable photoisomerization of azobenzene induces orientational angular hole‐burning, illustrated by the directional distribution of azobenzene long axes (pink dashed arrows) aligned orthogonally to the polarization of incident beam. (b) Photobirefringence in azopolymers, where polarized‐light‐induced molecular reorientation produces persistent anisotropy for write‐erase birefringence. Adapted with permission [3]. Copyright 2002, American Chemical Society. (c) Photoalignment of nematic LCs on azo‐dye‐coated polyimide; a light‐written azobenzene‐defined axis directs LC orientation without mechanical rubbing. The patterned “H” is observed under crossed polarizers. Adapted with permission [39]. Copyright 1991, Springer Nature Limited.

The photoorientation phenomenon in amorphous azobenzene‐containing polymers was systematically studied in the 1980s–1990s, notably by Todorov, Natansohn, Rochon and others [41, 42, 166, 167, 168], and became the basis for all‐optical poling [48, 49, 50, 51, 169, 170] and photoalignment techniques [39, 40, 73, 75]. The photoinduced anisotropy remains temporarily after the light is turned off (as long as the polymer is glassy and the chromophores are kinetically trapped) [3]. Natansohn and Rochon's 2002 review [3] emphasized that such photoinduced orientation is a universal feature of azobenzene‐containing polymers and can be harnessed to produce polarization holograms and birefringent elements (Figure 5b). In one important application, Gibbons et al. demonstrated photoalignment of LCs: they coated a surface with an azobenzene dye, exposed it to polarized visible light to induce a uniform in‐plane orientation of the chromophores, and then found that a nematic LC could align along this “photoinduced axis”, as presented in Figure 5c [39]. This provided a light‐based, non‐contact method to orient LC molecules as an alternative to the traditional mechanical rubbing of alignment layers. Ichimura, Seki, and others later expanded this into a robust technique for patterning LC orientations by light [40, 73, 75, 171, 172, 173], enabling advanced LC display (LCD) manufacturing and optical devices [75].

#### 2.3.2 All‐Optical Poling

A related consequence of photoinduced molecular reorientation is the generation of second‐order nonlinear optical (NLO) properties through purely optical means. Whereas conventional electric poling relies on a strong external field to induce non‐centrosymmetric alignment of dipolar chromophores required for second‐harmonic generation (SHG) and the Pockels effect [47, 174, 175, 176, 177], azopolymers can achieve similar symmetry breaking through coherent optical excitation, which selectively biases chromophore orientation and enables all‐optical poling (Figure 6a) [48, 49, 50, 51, 169, 178, 179]. This optical poling was demonstrated by J. M. Nunzi's group in the early 1990s [48]. In a common method, interfering a laser beam with its frequency‐doubled beam in an azopolymer can drive a slight one‐sided alignment that endows the material with a permanent SHG capability [48, 49, 50, 51]. Although the induced nonlinearity is smaller than that from electric poling, all‐optical poling is appealing for creating embedded electro‐optic structures without electrodes. In general, azobenzene‐containing chromophores have been widely used in NLO polymers because of their large hyperpolarizability and ease of orientation [47, 180]. Many push–pull azobenzenes such as Disperse Red 1 were integrated into polyimides or copolymers for electro‐optic modulators in the 1990s [47, 175, 176, 181, 182, 183], achieving significant Pockels coefficients. Nevertheless, maintaining long‐term stability proved difficult, as molecular relaxation of the chromophores gradually reduced the electro‐optic nonlinearity. To address this problem, researchers developed stable polymeric NLO materials, for instance, by crosslinking the polymer or attaching the azo units as side chains to “lock in” the oriented structure (Figure 6b) [170, 175, 176, 184, 185]. A comprehensive review in 2004 by Yesodha et al. surveyed these strategies [47], highlighting that azobenzene systems possess inherent advantages for high optical nonlinearities due to their combination of light‐induced isomerization, reorientation, and nonlinear absorption properties. Today, modern derivatives and polymer architectures continue to improve the stability of azobenzene‐based NLO devices, keeping them relevant for applications like electro‐optic switching and frequency conversion [47, 176, 179, 183].

**FIGURE 6:**
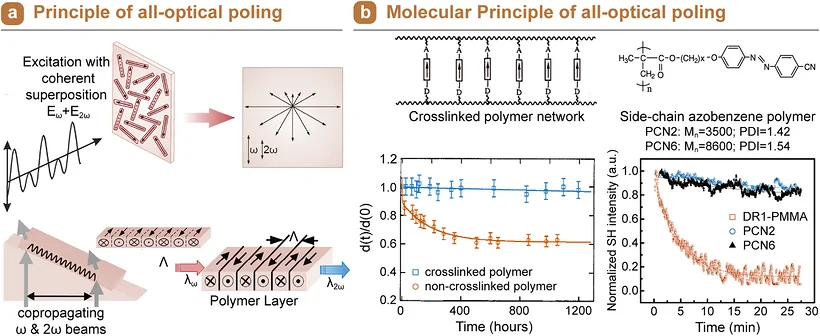
All‐optical poling process of azopolymers. (a) Coherent dual‐frequency field generated by interference system irradiates the azopolymer sample. In the sample, the resulting polar excitation upward and downward periodically induces the head‐to‐tail orientation of azobenzene units and breaks centrosymmetry, thereby enabling second‐harmonic generation (SHG). Adapted with permission [169, 179]. Copyright 2010, World Scientific Publishing Company (top panel), Copyright 2002, Society of Photo‐Optical Instrumentation Engineers (SPIE) (bottom panel). (b) Crosslinked polymer (left) and side‐chain azo polymer (right) structures used to lock in chromophore orientation for a stable nonlinear optical (NLO) system. Adapted with permission [170, 184]. Copyright 1993, American Chemical Society (left panel), Copyright 2005, Wiley‐VCH Verlag GmbH & Co. KGaA, Weinheim (right panel).

### 2.4 Photoinduced Surface Relief Gratings and Directional Mass Transport

Perhaps the most visually striking effect of light on azopolymer films is the formation of surface reliefs due to photoinduced, directional plastic deformations (called “mass transport” in earlier studies). In 1995, two groups (Rochon et al. [28] and Kumar et al. [29]) discovered that illuminating an interference pattern of light on an azopolymer thin film could cause the material to directionally deform and form the permanent SRGs: essentially a holographically inscribed sinusoidal corrugation on the surface with amplitude up to hundreds of nanometers. This was an astonishing finding. Unlike conventional photoresists, the azopolymer did not ablate or crosslink under the light; instead, it directionally transported in the solid state, with a mechanism ultimately driven by repeated *trans*–*cis* isomerization [171, 186, 187, 188, 189, 190, 191, 192, 193, 194, 195, 196]. The resultant gratings could efficiently diffract light, opening a new avenue for all‐optical holographic inscription and nanopatterning. Upon uniform light illumination, the SRGs can be erased and flat film retained [197, 198, 199].

In a typical experiment for SRG inscription, two coherent laser beams (often from an Ar^+^ laser working at 488 nm wavelength or diode‐pumped solid‐state laser working at 532 nm wavelength) intersect on the film, creating a periodic light intensity or polarization pattern (Figure 7a) [200]. After minutes of exposure, one observes a corresponding periodic modulation in the film thickness (Figure 7b) [200]. Considerable effort has been invested in establishing the correlation between the grating phase and the phase of the interference pattern [194, 201]. The most detailed and consistent interpretation was provided by the Santer group, which benefited greatly from in situ atomic force microscopy (AFM) characterization [201, 202]. In a complementary approach, Lee et al. projected an interference pattern onto azopolymeric pristine line arrays, where the grating vector of the interference pattern was deliberately designed orthogonal to that of the pre‐patterned lines [203]. Because the azopolymer could move not only along the horizontal direction of each line but also within the interline spacing perpendicular to it, this experimental geometry enabled a direct, 2D visualization of directional mass transport across all spatially varying polarization distributions of the interference pattern (Figure 7c) [194]. In particular, it turned out that in the case of an intensity interference pattern (IIP), where the light intensity is sinusoidally modulated along the grating vector without any polarization modulation, the intensity maxima correspond to valleys in the resulting polymer surface profile [194, 204, 205, 206, 207]. This indicates that the material moved away from the bright regions and accumulated at the intensity minima, although, in some cases, the opposite phase relationship can be observed depending on the specific polarization configuration of the interfering beams and material architectures [208, 209]. Note that an IIP alone is not enough for efficient SRG inscription (e.g., for mixing of *s*‐ and *s*‐polarized beams), which instead requires a non‐zero component of the electric field in the direction of the intensity gradient [3, 209].

**FIGURE 7:**
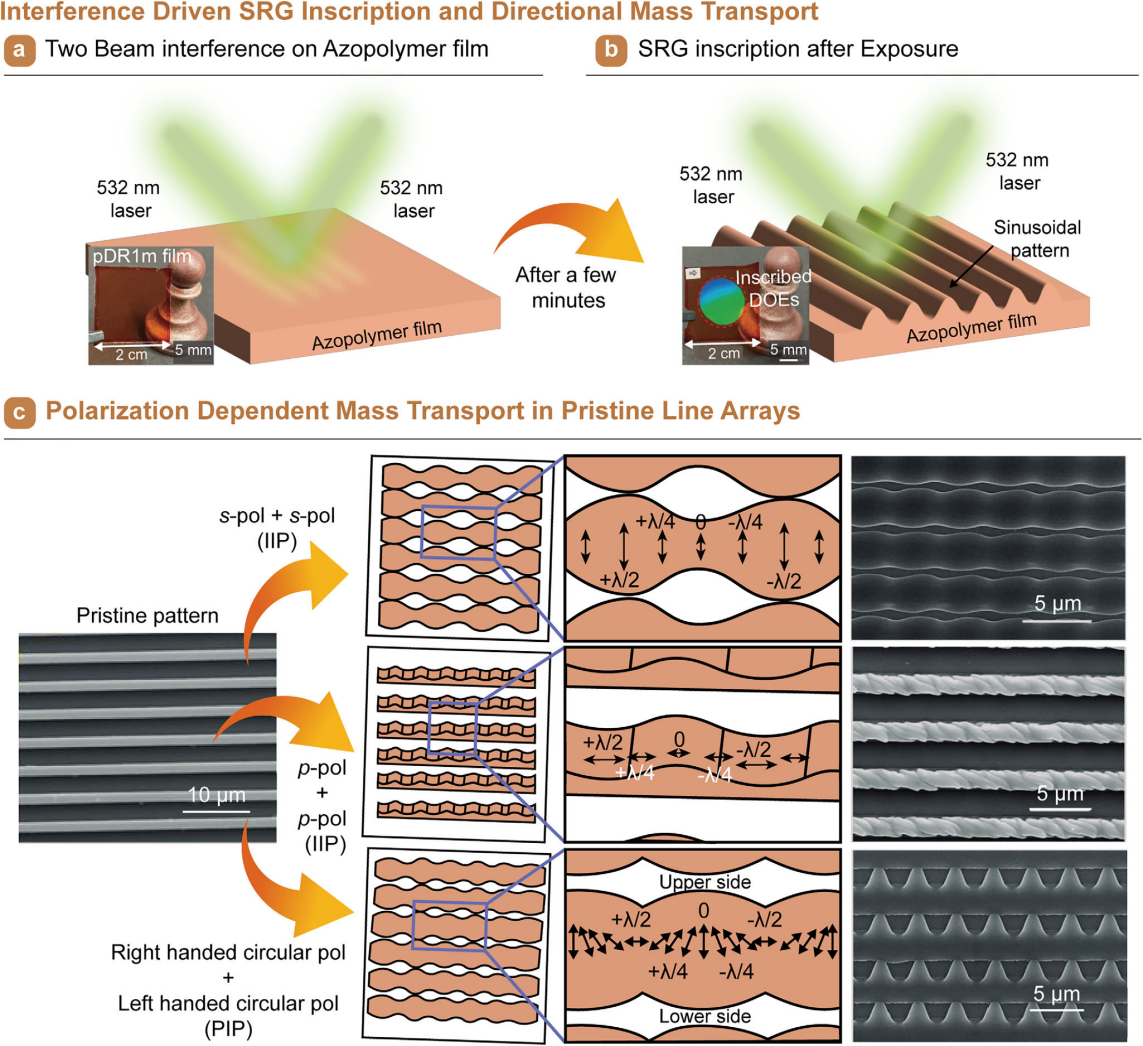
Photoinduced surface relief grating (SRG) formation and polarization‐dependent deformation in azopolymers. (a) Two‐beam interference setup generating periodic intensity or polarization patterns on the film. Adapted with permission [200]. Copyright 2021, Wiley‐VCH GmbH. (b) After irradiation, the azopolymer undergoes directional mass transport along the interference pattern, forming surface gratings. Adapted with permission [200]. Copyright 2021, Wiley‐VCH GmbH. (c) Under intensity interference pattern (IIP) and polarization interference pattern (PIP), the surface profile varies with the local polarization direction, showing polarization‐dependent line deformation. Adapted with permission [194]. Copyright 2011, Wiley‐VCH Verlag GmbH & Co. KGaA, Weinheim.

In contrast, under a polarization interference pattern (PIP), where the light intensity remains uniform but the polarization direction varies periodically along the grating vector, the regions with horizontal polarization typically evolve into valleys, suggesting polarization‐dependent material deformation even in the absence of intensity gradients [194, 204, 205, 206, 207]. Overall, it turned out that azopolymer undergoes directional plastic deformation (mass transport) just along the light polarization (Figure 7c) [194].

This phenomenon is not limited to SRG formation, but is also clearly evidenced through the directional photoreconfiguration of azopolymeric pristine patterns, which can be initially obtained by soft lithography (Figure 8). The method, first pioneered by S. Lee et al. in 2010, is sometimes referred to as directional photofluidization lithography (DPL) [203, 210, 211, 212, 213]. This terminology originated from phenomenological observations rather than a well‐defined physical mechanism, as was highlighted in a previous review [213]. At that time, the underlying mechanism responsible for the directional mass transport in azobenzene‐based materials had not yet been quantitatively established and remained largely inferred from phenomenological findings. For instance, when a single beam, which is linearly polarized along the grating vector of a soft‐lithographically defined 1D azopolymeric line array is illuminated onto the pattern, the line spacing decreases, effectively enhancing the pattern resolution (Figure 8a) [203]. The reconfigured azopolymeric patterns can subsequently serve as functional templates for material deposition processes, such as metal evaporation or other material coatings, followed by a standard lift‐off step, in a manner analogous to conventional photoresists. For instance, after metal deposition followed by a lift‐off process, the azopolymer pattern can act as a mask for metal patterning. Moreover, by increasing the light‐exposure time, finer metal line arrays can be achieved [203, 210]. Furthermore, illumination of a circularly polarized single beam onto azopolymeric pristine hole arrays results in a reduction of hole diameter, again leading to a resolution enhancement of the initial pattern (Figure 8b) [211]. This observation demonstrates that directional photodeformation in azopolymers can also be driven by circularly polarized light.

**FIGURE 8:**
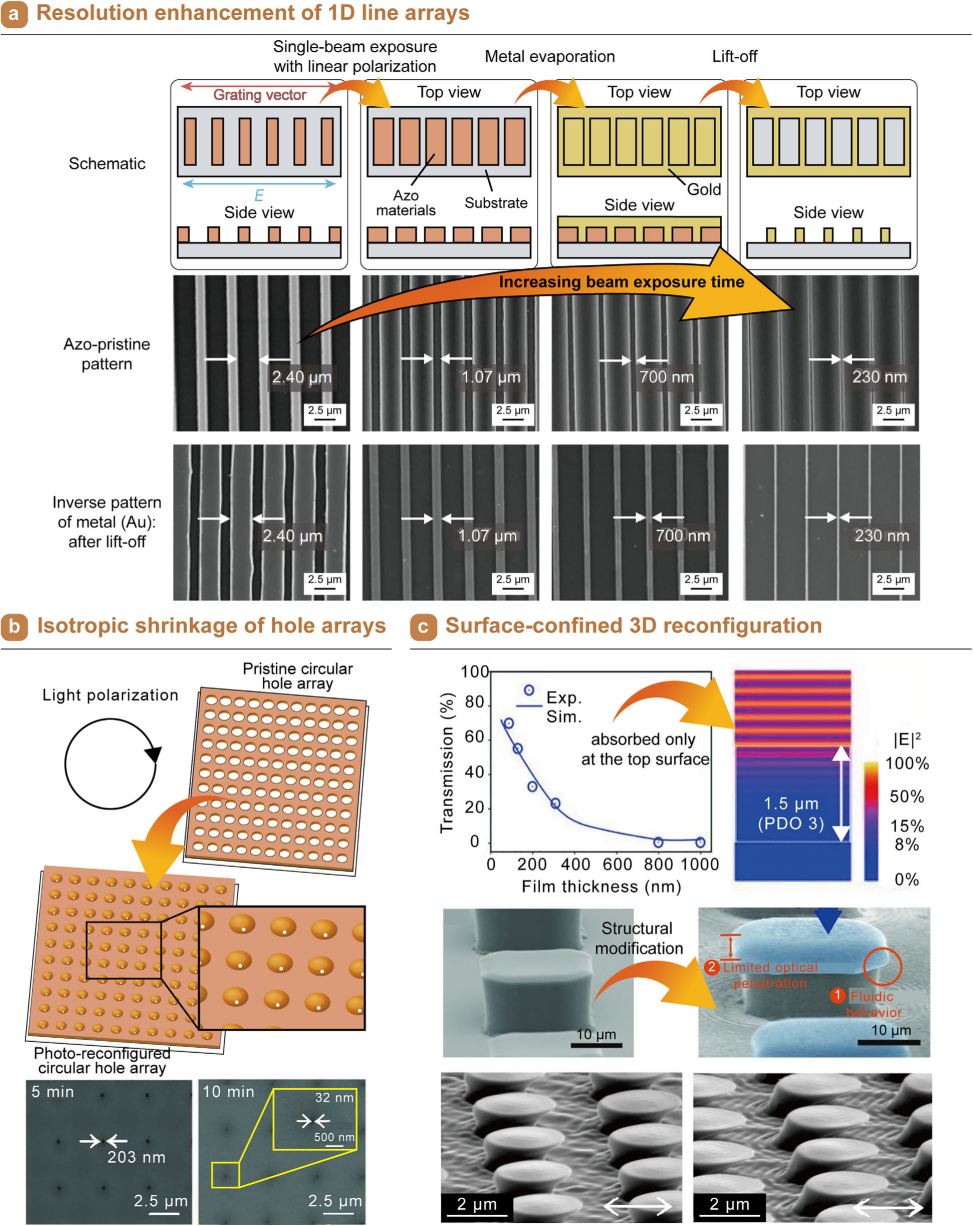
Directional photofluidization lithography (DPL) of azopolymeric pristine patterns. (a) Line arrays contract along the polarization direction under linearly polarized single‐beam irradiation, reducing line spacing. Adapted with permission [203]. Copyright 2010, American Chemical Society. (b) Circularly polarized irradiation of hole arrays causes isotropic diameter shrinkage. Adapted with permission [211]. Copyright 2011, Wiley‐VCH Verlag GmbH & Co. KGaA, Weinheim. (c) Depth‐controlled reconfiguration confined to the top layer enables 3D shaping from dome‐like to slanted microposts depending on polarization and beam incidence. Reproduced with permission [214, 215]. Copyright 2013, Wiley‐VCH Verlag GmbH & Co. KGaA, Weinheim (top panel), Copyright 2015, American Chemical Society (bottom panel).

Notably, by modulating the incident light intensity or wavelength so that the light is absorbed only at the top surface of the pristine pattern, the resulting directional reconfiguration can be confined exclusively to the surface layer, while the underlying thickness remains intact (Figure 8c) [214, 215]. This enables 3D and directional pattern reconfiguration, in which structural modification is confined to designated vertical regions within the azopolymeric pristine pattern. For instance, such surface‐confined mass transport can transform azopolymeric microposts with an initially isotropic top shape into either isotropically melted, dome‐like features (resembling a melting ice cream top) under circularly polarized single‐beam illumination without a topcoat flat layer, or into anisotropically slanted morphologies when a topcoat flat layer is attached on top and a linearly polarized beam is obliquely incident [212, 216, 217, 218, 219, 220]. These polarization‐dependent deformations highlight the intrinsic photomechanical anisotropy of azopolymers, where the direction of molecular reorientation and resulting mass transport follows the light polarization vector [221].

Very recently, this polarization‐dependent deformation principle was extended from globally uniform polarization fields to spatially programmed vectorial light fields, enabling model‐guided photoreconfiguration of individual azopolymer microstructures (Figure 9) [220]. As schematically shown in Figure 9a, an azopolymer micropillar can be irradiated with a vectorial light field whose local polarization angle, φ(*x*, *y*), varies across the illuminated area. In this configuration, the optical electric‐field vector *E* is no longer a single global direction; rather, each infinitesimal material volume, *dV*, experiences a locally defined polarization direction and corresponding light‐induced stress increments, including components parallel and perpendicular to *E* [220, 222]. The conceptual consequence is illustrated in Figure 9b: the spatial distribution of polarization‐induced stresses defines a programmable stress pathway that drives the micropillar from its initial shape (at illumination time *t* = 0) to a designed deformed morphology (at illumination time *t* = *t*′). For a symmetric vector‐field design with Δφ(*x*) = +π across the pillar (Figure 9c), the programmed polarization map generates a symmetric stress pathway (Figure 9d), resulting in time‐dependent, bending‐like deformation of the micropillar. The corresponding experimental images and viscoplastic photoalignment (VPA) model simulations (to be further discussed in the next section) show close agreement over illumination time (*t*) of 0, 15, 45, and 60 s (Figure 9e) [220, 223]. By contrast, an asymmetric vector‐field design, in which the polarization rotation changes from Δφ(*x*) = +π/2 on one side to Δφ(*x*) = −π/2 on the other side (Figure 9f), creates an asymmetric stress pathway (Figure 9g). This asymmetric stress distribution reconfigures the initially similar micropillar into a chiral‐like, twisted morphology, again with experiment and model showing consistent temporal evolution (Figure 9h) [220, 224]. Therefore, this study advances azopolymer photodeformation from phenomenological directional mass transport to a predictive design framework: molecular photoalignment and polymer‐backbone reorientation are translated into local stress fields, and those stress fields are further converted into programmable 3D microarchitectures [220, 222, 223, 224].

**FIGURE 9:**
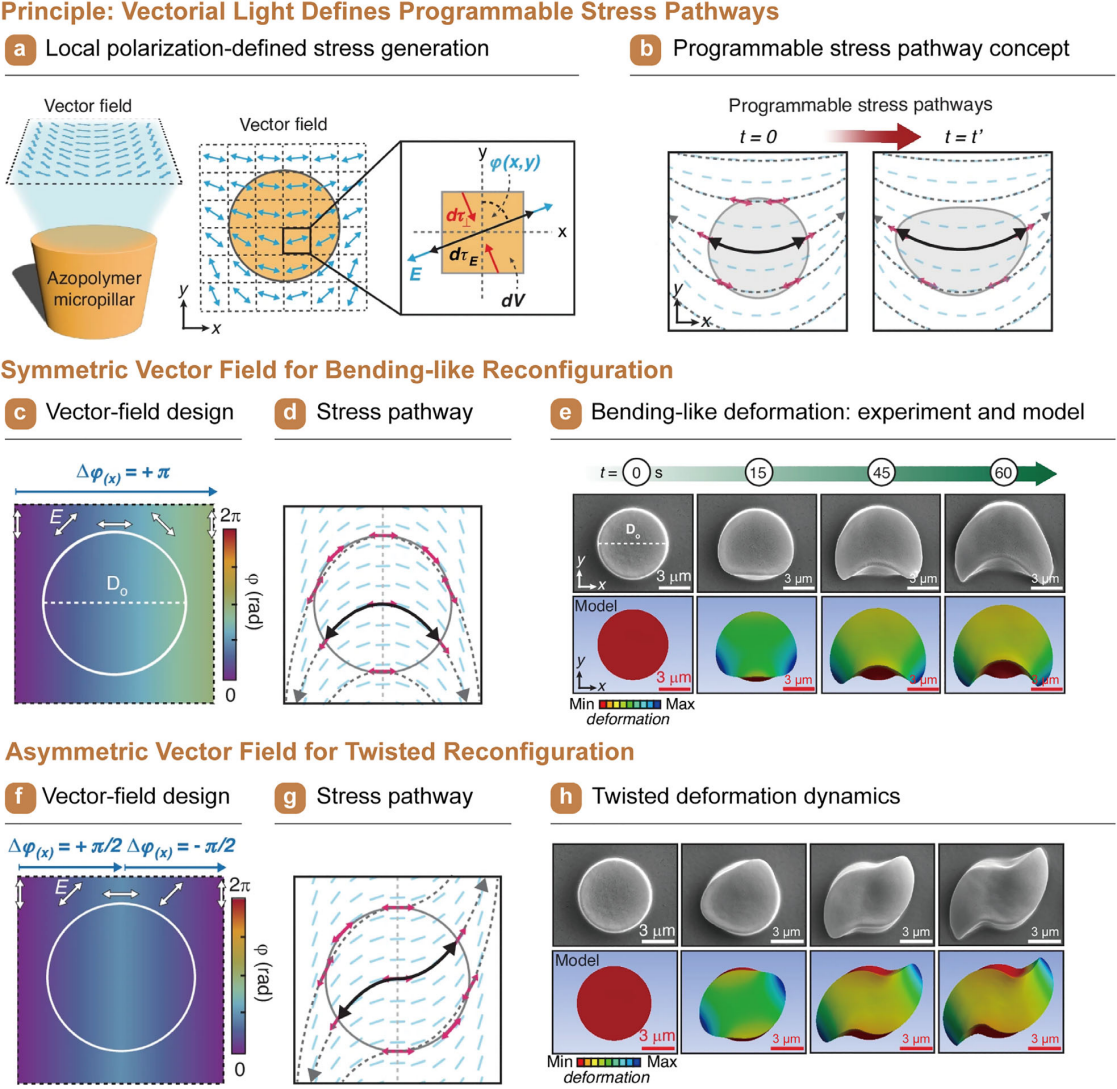
Vectorial‐light‐field‐guided stress‐driven photoreconfiguration of azopolymer micropillars. (a) Schematic of an azopolymer micropillar irradiated with a spatially programmed vectorial light field. The local polarization angle, φ(*x*, *y*), defines the optical electric‐field direction *E* at each position and generates local light‐induced stress increments within an infinitesimal material volume, *dV*. (b) Conceptual illustration of programmable stress pathways: the initial micropillar geometry at *t* = 0 evolves into a designed deformed morphology at *t* = *t*′ according to the spatial distribution of polarization‐induced stresses. (c–e) Symmetric vector‐field design with Δφ(*x*) = +π across the micropillar. (c) Programmed polarization map and local electric‐field orientations, (d) corresponding stress pathway predicted by the vectorial field, and (e) time‐resolved experimental deformation of an azopolymer micropillar at 0, 15, 45, and 60 s, compared with model predictions. (f–h) Asymmetric vector‐field design with opposite polarization rotations, Δφ(*x*) = +π/2 and Δφ(*x*) = −π/2, on different sides of the micropillar. (f) Programmed polarization map, (g) resulting asymmetric stress pathway, and (h) time‐resolved experimental and modeled deformation showing chiral‐like morphology evolution. The model images use a color scale from minimum to maximum deformation. Scale bars: 3 µm. Panel (a–h) reproduced under the terms of the Creative Commons Attribution 4.0 International License [220]. Copyright 2026, The Author(s), published by Springer Nature.

Building on such polarization‐dependent deformations, beyond its established roles in SRG formation and DPL, directional mass transport in azopolymers has emerged as a powerful mechanism capable of offering an alternative approach to conventional nanofabrication. A representative example is athermal “photonanoimprinting”, in which flat azopolymeric thin films can be patterned under a mechanically applied mold at room temperature during light illumination [215, 225, 226, 227]. This process stands in stark contrast to conventional thermal nanoimprint lithography (NIL), which typically requires heating above the glass transition temperature (*T_g_*) to soften the resist (e.g., poly(methyl methacrylate) (PMMA)) and the application of high mechanical pressure [228, 229, 230]. Consequently, azobenzene's directional photoinduced mass transport enables nanoimprinting under ambient temperature and pressure, effectively reducing dependence on specialized high‐temperature and high‐pressure infrastructure.

Beyond nanofabrication, directional mass transport offers unique opportunities in flexible and wearable electronics. For instance, an azopolymer layer coated with a silver nanowire mesh can function as a deformable conductor whose fractures or microcracks, which could be formed during repeated bending or twisting, can be autonomously healed through light‐driven directional transport of the azopolymer [231]. A related concept was further demonstrated in light‐transformable and light‐healable triboelectric nanogenerators (TENGs), where azopolymer surface reliefs were deliberately sculpted to increase the effective contact area and thereby enhance triboelectric output [232]. In these devices, the output voltage and current scaled with the surface area of the azopolymer reliefs, but repetitive mechanical contact gradually collapsed and flattened the soft surface structures, leading to continuous performance degradation. Importantly, light‐induced migration restored both the damaged surface morphology and the TENG output, demonstrating that azopolymer mass transport can serve not only as a pattern‐writing mechanism but also as an in situ repair strategy for mechanically stressed flexible energy‐harvesting devices [231, 232]. In parallel, poly(ionic liquid) films incorporating azobenzene‐based ionic liquids have broadened the scope of azobenzene‐enabled adaptive electronics beyond topographic repair [233]. In these PIL‐AzoIL films, UV‐induced *trans*‐to‐*cis* isomerization increases molecular polarity and ion mobility, resulting in higher ionic conductivity, whereas visible light restores the *trans*‐rich state and lowers the conductivity [233]. The films also exhibit self‐healing behavior and can operate as visual sensors for trifluoroacetic acid vapor, illustrating how azobenzene switching can couple mechanical repair with reversible ion‐transport modulation [233].

In addition to such device‐level and fabrication applications, this phenomenon also provides a simple optical route to visualize nanoscale near‐fields without the need for expensive and complex near‐field scanning optical microscopy (NSOM) instrumentation. Because azopolymeric surfaces undergo directional deformation that faithfully follows the local electric‐field topology of plasmonic nanostructures, one‐shot near‐field mapping can be achieved over large areas by recording the resulting surface‐relief distribution [234, 235, 236]. Collectively, these capabilities demonstrate that azopolymer‐based directional mass transport, once regarded as a phenomenon limited to SRG formation, has evolved into a versatile platform spanning both practical technologies, such as nanofabrication and self‐healing electronic devices, and fundamental studies of light‐matter interactions at the nanoscale.

#### 2.4.1 How Can Light‐Driven Mass Transport be Induced?

The mechanism of this light‐driven directional mass transport has been the subject of intense study and some controversy. More than 20 models have been proposed over the years to explain this astonishing phenomenon. Their advantages and disadvantages are discussed in the recent theoretical review [206]. Here we briefly present the most popular models:

##### 2.4.1.1 Optical Gradient Force Model

One early explanation was that the electric field gradient in the interference pattern exerts a force acting on polarized azopolymer medium (Figure 10a). This model was particularly appealing because it readily accounted for the strong polarization dependence observed experimentally: *p*‐ and *p*‐polarized beam mixing‐based IIP produced well‐defined SRGs, whereas *s*‐ and *s*‐polarized beam mixing‐counterpart yield nearly no modulation [201, 206, 237]. However, in 2004 Saphiannikova et al. demonstrated that the predicted force density is negligible, effectively undermining this approach [238]. To address the observed mass transport, Kumar and coworkers suggested that efficient cyclic isomerization dramatically reduces the viscosity of azopolymers down to values comparable to that of water [237]. This idea gave rise to the concept of a “liquidized” or later “photofluidized” azopolymer [3]. Despite several subsequent modifications, none succeeded in overcoming the fundamental limitation of the model: the optical gradient forces remain far too weak to account for the experimentally observed material transport [206, 239].

**FIGURE 10:**
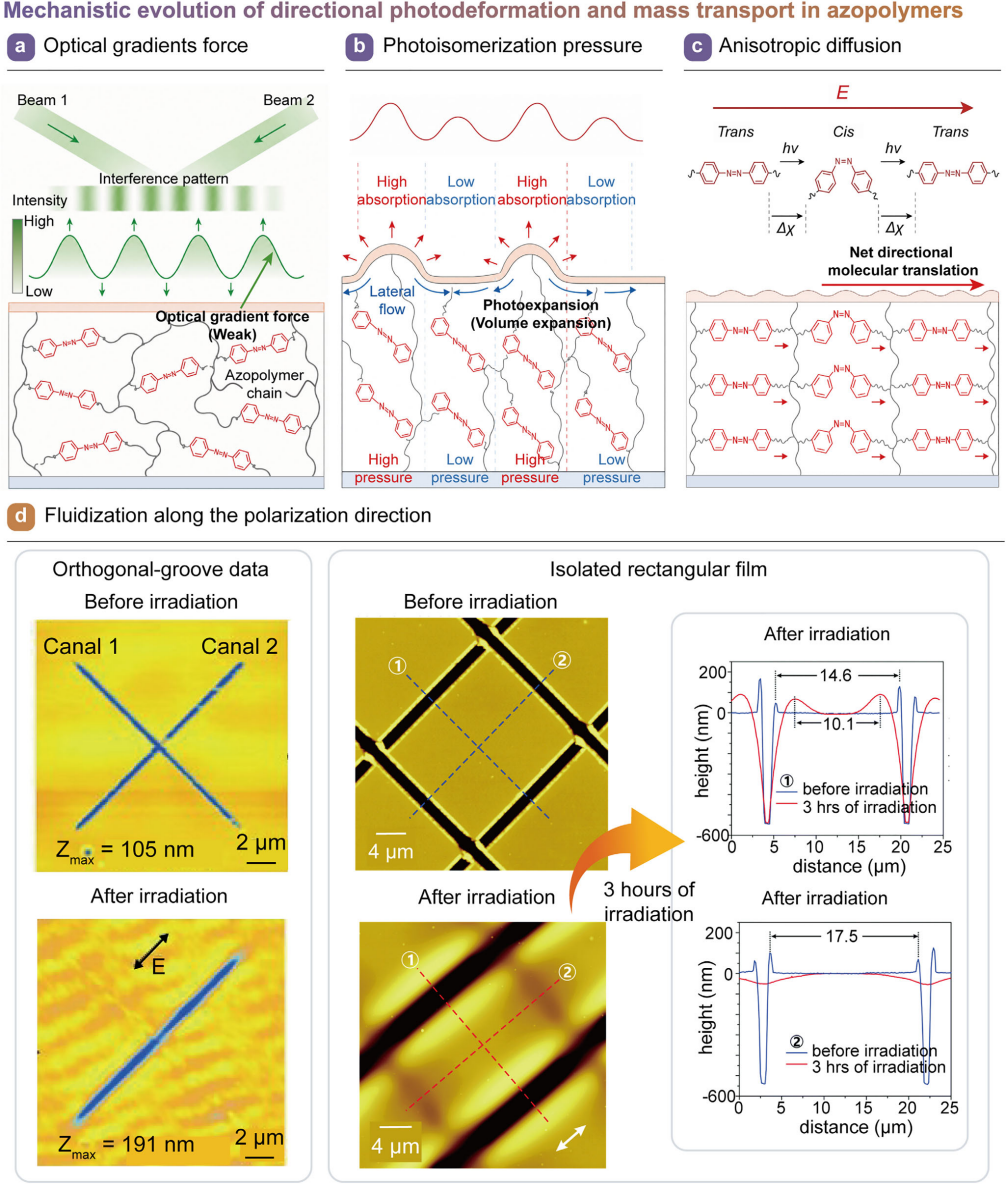
Historical development and experimental evaluation of proposed mechanisms for directional photodeformation and mass transport in azopolymers. (a–c) Earlier models based on optical gradient forces, photoisomerization pressure, and anisotropic diffusion were developed mainly to explain SRG formation, but each has important limitations: [3] the optical gradient force is too weak [52, 238, 239], the pressure model cannot explain PIP‐driven SRGs with nearly uniform intensity [240, 241], and anisotropic diffusion requires unrealistically large molecular translation steps while neglecting polymer‐matrix coupling [242, 243, 244]. (d) The photofluidization concept was supported by groove‐filling experiments and modest photosoftening of azopolymers, but re‐examination using isolated rectangular film pieces showed simultaneous groove closing and widening, indicating that softening alone is insufficient and that directional opto‐mechanical stress is required [204, 245]. Orthogonal‐groove data reproduced with permission [245]. Copyright 2005, Springer Nature Limited. Isolated rectangular film data reproduced with permission [204]. Copyright 2016, The Royal Society of Chemistry.

##### 2.4.1.2 Photoisomerization Pressure Model

As azobenzene molecules isomerize, they can push against the polymer matrix. Barrett et al. and others proposed that each *trans*–*cis*–*trans* cycle imparts a tiny force, and billions of such cycles collectively generate stresses that drive mass movement (Figure 10b) [159, 240, 241]. In essence, the polymer behaves like a photoexpanding material in regions of high photon absorption, causing a move to relieve stress. The pressure gradients cause the viscous flow from the high‐pressure areas to lower‐pressure areas, which would explain IIP‐driven formation of SRGs [159, 240]. However, PIP‐driven formation of SRGs cannot be rationalized by this model, since PIP patterns have constant light intensity and would not give rise to any pressure gradients [241].

##### 2.4.1.3 Anisotropic Diffusion Model

The anisotropic diffusion model was introduced by Nunzi et al. to explain SRG formation via photoinduced migration of azobenzenes (Figure 10c) [51, 242, 243, 244]. They proposed that after each photoisomerization cycle, an azo‐molecule undergoes a small translation along its long molecular axis, leading to anisotropic diffusion [242, 243]. The migration of azo‐molecules along the grating vector was linked to the observed SRG amplitude. However, the assumed translation distances up to 100 nm were physically unrealistic. The model also failed to describe how molecular displacement transfers to the polymer matrix, treating the host as a passive medium [244]. In 2006, Bellini et al. reformulated the idea as a random‐walk model of molecular motors, suggesting that light polarization gradients could drive 1D motion of azobenzenes along the polarization direction [246]. This modification shifted the focus from the intensity pattern to polarization gradients but still neglected polymer matrix and molecular reorientation effects. A related photoinduced molecular diffusion (PIMD) approach by Juan et al. used Monte Carlo simulations with unphysically large translation steps (up to 150 nm) and effectively treated azobenzenes as moving in vacuum [247, 248]. While these simulations reproduced some trends for the Gaussian beam and IIP‐driven formation of SRGs, they cannot explain deformations under uniform illumination.

Overall, the anisotropic diffusion framework was an early attempt to rationalize SRG formation in terms of molecular transport, but it overestimated the mobility of azobenzenes and could not account for experimentally observed deformations in the absence of optical gradients [244].

##### 2.4.1.4 Photofluidization Concept

The influential idea of a “liquidized” azopolymer inspired numerous experimental efforts aimed at verifying whether light illumination could significantly reduce the material's mechanical parameters. However, only weak photosoftening effects have been consistently observed [249]. For instance, Karageorgiev et al. reported that the elastic modulus decreases by a factor of four (from 3.4 to 0.9 GPa), while the light‐induced viscosity remains as high as 4 GPa × s [245]. In contrast, strong softening associated with thermal heating of the azopolymer near its *T_g_* was found to be detrimental to SRG formation [250, 251, 252].

Despite this, a selective filling of grooves oriented perpendicular to the polarization direction (left panel of Figure 10d) [245] was interpreted by Karageorgiev et al. as evidence for anisotropic photofluidization: “Whereas during irradiation with circularly polarized light the film behaves as an isotropic viscoelastic fluid, under linearly polarized light it shows considerable fluidity only along the direction parallel to the light field vector.” The concept of anisotropic fluidization was re‐examined in 2016 by the groups of Hurduc and Santer [204]. Repeating Karageorgiev's experiment with an isolated rectangular film piece (right panel of Figure 10d) [204], they observed that after 3 h of irradiation, one groove closed while the other widened, the latter effect also being noted in the original study. This behavior is more consistent with viscoplastic deformation than with fluidization. Indeed, the simple concept of anisotropic fluidization cannot account for simultaneous stretching in one direction and contraction in the perpendicular one; a directed force must be responsible for the anisotropic material displacement. Ultimately, both groups concluded that the induction of opto‐mechanical stresses is a necessary prerequisite for the photodeformational response in glassy azopolymers [204, 245].

##### 2.4.1.5 Orientation Model

The first orientation‐based explanation for SRG formation was proposed by Bublitz et al., who attributed surface deformation to the reorientation of side‐chain azobenzene with respect to the light polarization [207]. Their model successfully reproduced several experimental trends but neglected the mechanical coupling between chromophore orientation and the polymer matrix. Saphiannikova and Neher later introduced a thermodynamic theory linking the loss of orientational entropy to light‐induced stress, providing the first quantitative stress estimate (∼5 MPa) [244]. These early models established the key concept that chromophore reorientation drives mechanical deformation, laying the foundation for the modern orientation approach.

The modern orientation approach, developed by Saphiannikova and coworkers, provides a unified framework linking light‐induced reorientation of chromophores to mechanical stress and deformation in azopolymers (Figure 11a) [206, 207, 221, 222, 244]. It introduces an effective orientation potential, which describes how polarized light drives reorientation of azobenzenes [253, 254]. This potential enables quantitative prediction of light‐induced stresses up to ∼100 MPa, exceeding the yield stress of glassy azopolymers, and explains both reversible and plastic deformations. The model accounts for molecular architecture through the coupling between chromophore orientation and alignment of polymer backbones. It predicts that the sign of light‐induced stress depends on the orientation distribution of chromophores around the backbones [221, 222].

**FIGURE 11:**
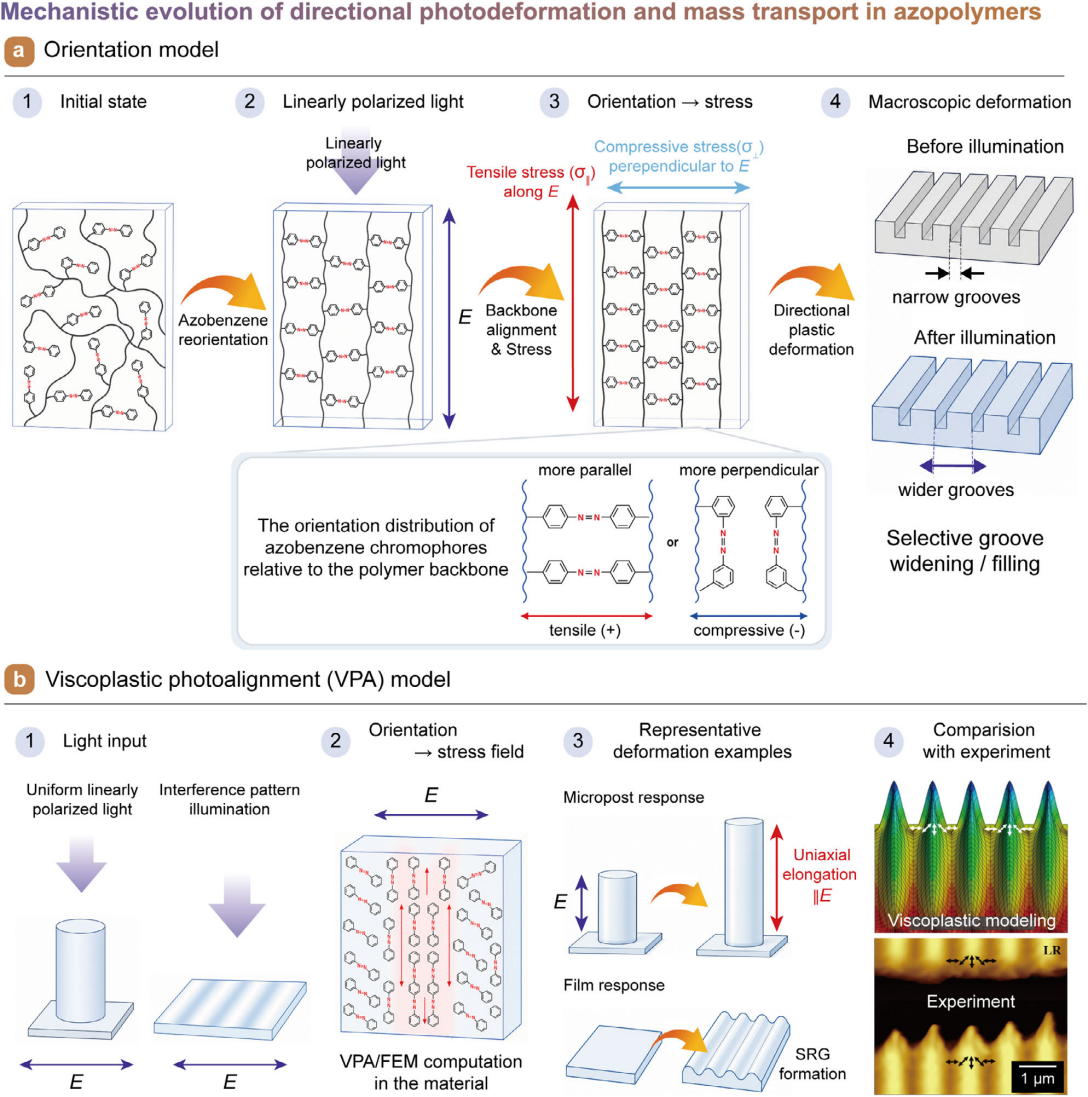
Orientation‐ and stress‐based mechanisms of directional photodeformation in azopolymers. (a) Under linearly polarized light, repeated *E/Z* photoisomerization reorients *trans* azobenzene chromophores perpendicular to the electric field vector *E*. For the representative side‐chain architecture, the couping between chromophores and backbones aligns the polymer backbones along *E*, generating tensile stress parallel to *E* and compressive stress perpendicular to *E*, which elongates the material along *E*. The stress sign depends on the chromophore orientation relative to the polymer backbones [206, 207, 221, 222, 244]. (b) The viscoplastic photoalignment (VPA) model extends this orientation framework into finite‐element viscoplastic mechanics [206, 223] and provides a unified description of micropost/colloid elongation [220, 222], SRG formation under IIP and PIP, and asymmetric or edge‐localized deformation under complex polarization fields [224, 255]. Overall, this model describes directional photodeformation as a cross‐scale process from *E*/*Z* photoisomerization to chromophore reorientation, backbone alignment, light‐induced stress, and viscoplastic mass transport. Edge‐protrusion experiment and VPA modeling. Adapted under the terms of the Creative Commons Attribution 4.0 International License [255]. Copyright 2022, The Authors, published by Wiley‐VCH GmbH.

The orientation approach readily explains why linearly polarized light induces selective filling of grooves [204, 245]. This occurs because reorientation of polymer backbones along the electric field vector leads to plastic elongation of the side‐chain azopolymer in the same direction. Consequently, grooves aligned parallel to the polarization direction are predicted to widen, in full agreement with experimental observations [222, 224, 255].

##### 2.4.1.6 Viscoplastic Photoalignment (VPA) Model

To predict photoinduced plastic deformations, the modern orientation approach has been implemented in finite element modeling (FEM) software [206, 222, 223, 255]. Since glassy azopolymers undergo plasticization under sufficiently large light‐induced stresses, analogous to metals such as steel, an elastic–viscoplastic constitutive model was adopted to describe their mechanical response [206, 223]. The light‐induced stress at each material point is computed directly from the change in the orientation state of polymer backbones. This formulation, known as the VPA model [223, 256], successfully explains directional photodeformation in side‐chain azopolymers [222, 224].

VPA modeling reproduces the experimentally observed uniaxial elongation of microposts (Figure 11b) [206, 220, 222, 223] and colloids along the polarization direction, even under uniform illumination, confirming that optical gradients are not required to drive deformation [222, 223, 255, 256]. When applied to light interference patterns, it explains the phase and amplitude of SRGs inscribed by IIP (e.g., mixing of *p*‐ and *p*‐polarized beams or *s*‐ and *s*‐polarized beams) and PIP (e.g., mixing of right‐handed circularly polarized (RCP) and left‐handed circularly polarized (LCP) beams), in agreement with experiment [206, 223, 255]. A faster growth under the PIP is shown to be promoted by a weak photosoftening effect [204]. Moreover, the asymmetric SRGs observed under elliptically polarized light‐based IIP are accurately captured, with the principal stress axes aligned along the axes of the polarization ellipse [206, 224, 254]. A striking resemblance of modeling and experiment is observed for peculiar structures protruding from the edges of an azopolymer film (Figure 11b) [206, 255]. Altogether, the VPA approach demonstrates that spatial modulation of light‐induced stress, rather than anisotropic diffusion or photofluidization, governs SRG formation, establishing the orientation mechanism as a universal framework for photoinduced directional deformations and mass transport in azopolymer [206, 222, 223, 249].

#### 2.4.2 Remarkable Libraries of SRGs and Other Patterns

Decades of study have not completely settled the mechanistic details, and it is likely that multiple factors work in concert. What is universally agreed is the phenomenology: azopolymer films under structured light or a single beam undergo molecular‐scale motions that integrate into large‐scale “directional” mass transport just along the light polarization, resulting in surface topography or directional pattern reconfiguration that matches the light intensity or polarization pattern [3, 241, 257]. More critically, because azopolymer photopatterning is reconfigurable (the patterns can be erased by heating the polymer above T_g_ or by uniform visible‐light exposure and then rewritten) [186, 202, 258], it offers a rewritable nanopatterning medium [199, 259, 260, 261]. Traditional lithography often requires multiple steps (coating, exposing, and etching), but azopolymer SRG inscription is a single‐step, light‐only process, which can even be done with a “laser printer” approach using scanning beams [58, 205, 241, 262]. The versatility of this effect is remarkable: by appropriately designing the interfering beams, for instance, through combinatorial mixing and overwriting of 1D IIP or PIP with different grating vectors, one can inscribe a wide variety of periodic and quasi‐periodic structures onto a flat surface [58, 260, 263, 264, 265]. These include tetragonal lattices (Figure 12a) [58], hexagonal arrays (Figure 12b) [58], quasi‐crystalline lattices (Figure 12c,d) [58], and even more complex moiré patterns (Figure 12e) [58]. Such deterministic SRG texturing can be realized even on curved substrates, since the inscription relies entirely on directional mass transport rather than conventional lithographic etching (Figure 12f) [266, 267]. Also, exposing an azopolymeric film to a vortex beam carrying orbital angular momentum led to spiral relief patterns (Figure 12g), as demonstrated by Ambrosio et al. [268]. By using computer‐generated holograms or a digital polarization rotator to modulate the writing beams [205, 269], researchers have created complex, hierarchically structured surfaces in a single exposure (Figure 12h) [269] or multiple exposures [270, 271].

**FIGURE 12:**
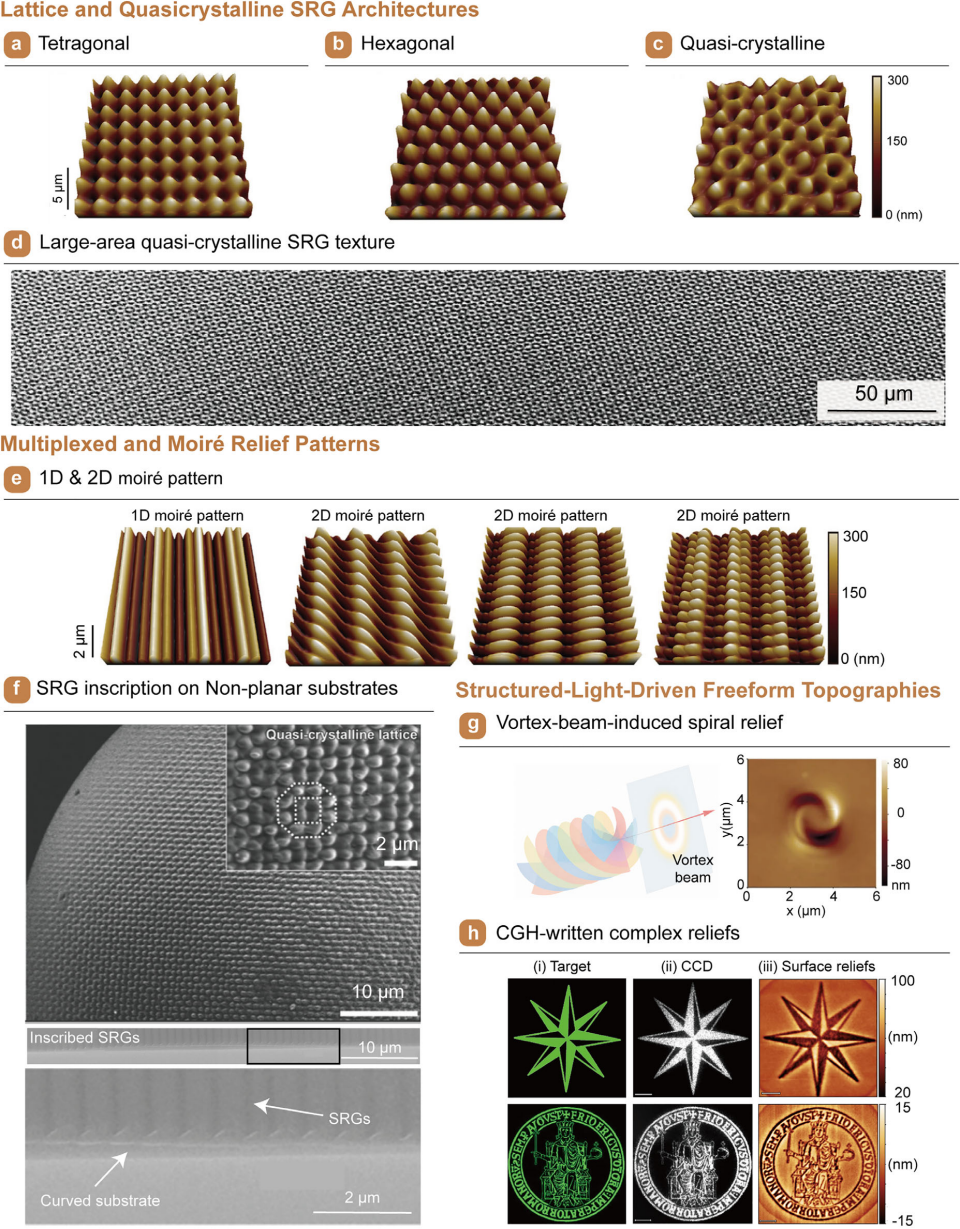
Versatile SRG inscription and reconfigurable nanopatterning in azopolymers. (a–d) AFM images of (a) 2D tetragonal SRGs, (b) 2D hexagonal SRGs, (c) 2D quasicrystal SRGs and (d) SEM image of 2D quasicrystal SRGs. Reproduced with permission [58]. Copyright 2021, Wiley‐VCH GmbH. (e) Moiré structures by sequential overwriting. Reproduced with permission [58]. Copyright 2021, Wiley‐VCH GmbH. (f) Deterministic SRG inscription realized even on a curved substrate owing to polarization‐driven directional mass transport. Adapted with permission [266, 267]. Copyright 2016, Wiley‐VCH Verlag GmbH & Co. KGaA, Weinheim (top panel); Copyright 2017, American Chemical Society (bottom panel). (g) Spiral relief structures formed by irradiation with a vortex beam carrying orbital angular momentum. Adapted with permission [268]. Copyright 2012, Macmillan Publishers Limited. (h) Complex, hierarchically structured patterns generated in a single exposure using computer‐generated holograms or digital polarization modulators. Reproduced under the terms of the Creative Commons Attribution 4.0 International License [269]. Copyright 2019, The Authors, published by Springer Nature.

Despite this progress, some fundamental questions remain. Even after 30 years of research, the precise microscopic mechanism of photoinduced mass transport is still being investigated using various theoretical and experimental methods. Researchers continue to examine how the nanoscale motion of individual chains translates to the mesoscale plastic flow that forms gratings [3, 206, 268]. On the one hand, molecular dynamics simulations of nanoscopic azopolymer samples are performed to confirm that light‐induced orientation of azobenzenes is transferred to an orientation and stretching of polymer backbones along the polarization direction [272, 273]. On the other hand, VPA modeling is applied to more and more complex irradiation patterns, including fully structured light [220, 223, 224]. This helps to understand the role of boundary effects and to elucidate the parameters of light‐induced plastic flow [206]. Advanced real‐time measurements, like digital holographic microscopy and diffraction monitoring, are being used to observe SRG formation in situ [201, 264, 274, 275]. What is clear is that azopolymers represent a unique class of light‐responsive materials that are sensitive to the vectorial nature of the light field [220]. The ability to inscribe motion and patterns by light (i.e., essentially turning optical energy directly into mechanical work and structure) is a distinguishing power of azobenzene‐based materials [11, 12, 206].

### 2.5 Photomechanical Effects

If light can move molecules and drive directional mass transport in glassy azopolymers, it can also induce macroscopic, reversible mechanical deformations [11, 12, 24, 276]. Photomechanical effects refer to reversible changes in shape or dimensionality of a material under light, and azobenzene has enabled a wide variety of such phenomena in both polymer films and bulk elastomers [24, 276, 277].

#### 2.5.1 Contraction, Bending, and Actuation

The simplest example of azobenzene photomechanics is observed in thin amorphous azopolymer films subjected to uniform light illumination [240, 278, 279, 280]. Upon exposure, *trans* isomers convert to *cis*, which occupies a larger free volume, leading to a small but measurable change in thickness [240, 281, 282, 283]. Tanchak and Barrett reported a repeatable and reversible thickness increase in polymer thin films containing push‐pull azobenzenes under visible‐light illumination (Figure 13a) [240]. The underlying photomechanical response arises from repetitive *trans–cis–trans* cycling of the azobenzene molecules, resulting in reversible expansion and contraction on the order of 1%–2% [240].

**FIGURE 13:**
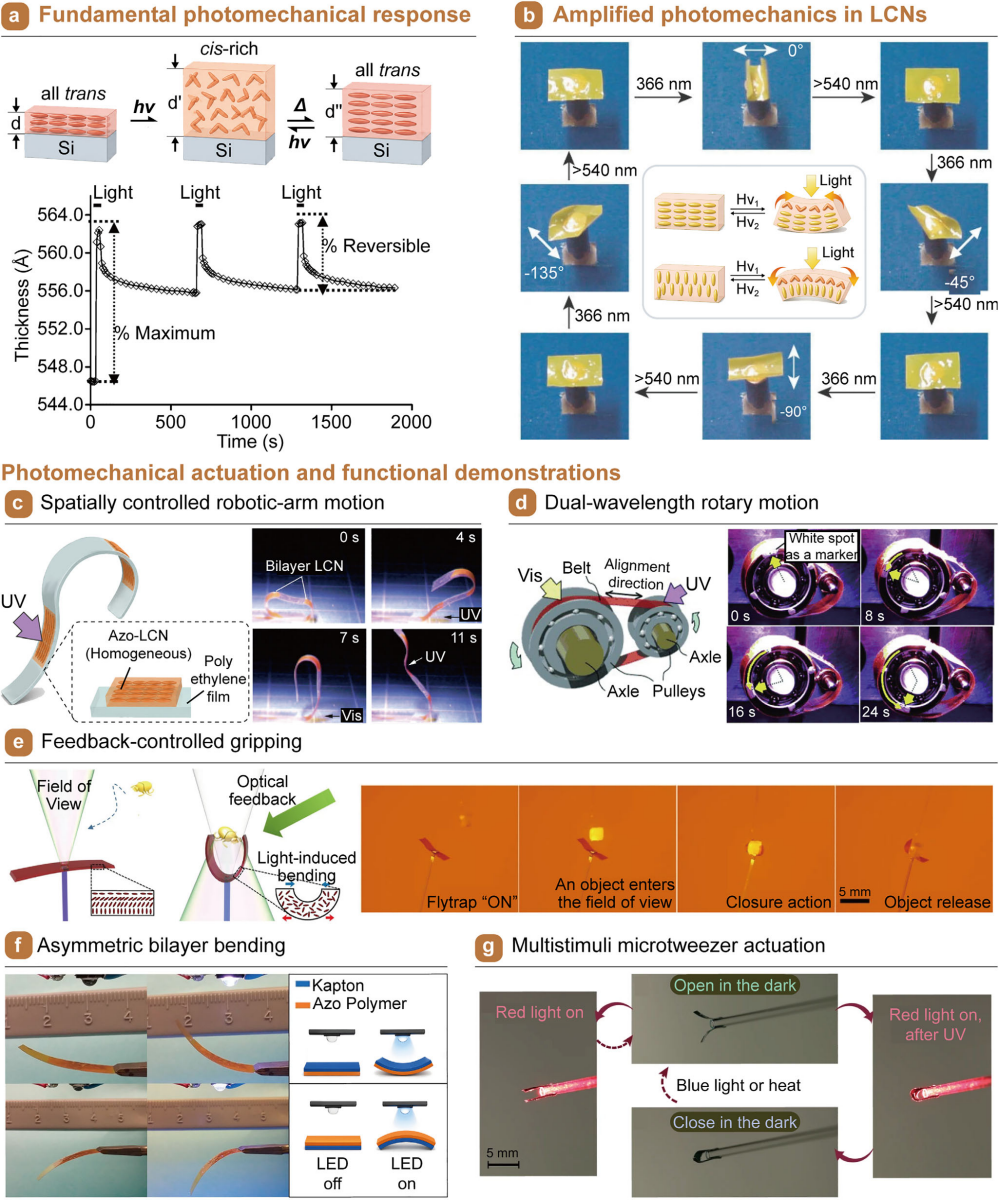
Photomechanical contraction, bending, and light‐fueled actuation in azobenzene‐containing systems. (a) Under uniform illumination, amorphous azopolymer films exhibit a small yet repeatable thickness increase driven by photoisomerization cycling, yielding stepwise expansion‐relaxation traces. Adapted with permission [240]. Copyright 2005, American Chemical Society. (b) In aligned LCNs, UV and visible light produce reversible bending whose sense and magnitude are dictated by the director orientation and by the illumination wavelength/polarization. Reproduced with permission [45, 69]. Copyright 2003, Springer Nature Limited; Copyright 2011, WILEY‐VCH Verlag GmbH & Co. KGaA, Weinheim (inset panel). (c) Spatially and wavelength‐controlled robotic‐arm motion of a bilayer LCN strip. Adapted with permission [284, 285]. Copyright 2009, The Royal Society of Chemistry; Copyright 2011, Elsevier Ltd. (d) Light‐fueled plastic motor by continuous rotation with dual‐wavelength irradiation. Reproduced with permission [70]. Copyright 2008, Wiley‐VCH Verlag GmbH & Co. KGaA, Weinheim. (e) A light‐driven artificial flytrap gripper closes and releases on demand, enabling autonomous feedback‐controlled capture. Adapted with permission [68]. Copyright 2017, Springer Nature. (f) Non‐LC bilayers bend when only one side (azobenzene polymer layer) contracts or expands under light‐emitting diode (LED) illumination. Adapted with permission [286]. Copyright 2018, American Chemical Society. (g) Multi‐stimuli micro tweezers switch between open and closed states under visible/thermal inputs, allowing reversible, contactless manipulation. Adapted under the terms of the Creative Commons Attribution 4.0 International License [287]. Copyright 2018, The Authors, published by Springer Nature.

The effect can be greatly amplified when the azobenzenes are incorporated into LCNs rather than amorphous matrices [24, 288, 289]. Liu, Broer, and coworkers have extensively investigated such systems, employing both uniformly aligned LCNs and those with spatially patterned alignment to achieve photocontrollable, contraction/expansion‐based surface deformations [290, 291, 292, 293]. Their studies also revealed that while film thickness variations can be induced by *trans–cis* isomerization alone, the most pronounced changes emerge under continuous *trans–cis–trans* oscillatory photodynamics, highlighting the importance of molecular cycling in maximizing macroscopic thin‐film actuation [293].

Transitioning from surface‐bound thin films to free‐standing LCN strips, more pronounced photomechanical responses can be achieved [24, 45, 289, 294, 295]. In LCNs, azobenzene mesogens are covalently attached as side‐chains or as crosslinks in densely crosslinked glassy LCNs or lightly crosslinked LCEs [25, 289, 294]. The pioneering demonstrations were reported in the early 2000s by the groups of Finkelmann [44], Keller [295], and Ikeda [26, 27, 45], whose study in 2003 provided an early demonstration: a thin LCN film containing azobenzene crosslinks that bends under UV irradiation, and reverts to its original shape upon visible‐light exposure (Figure 13b) [45, 69]. The bending was fully reversible and highly repeatable, and its direction was governed by the polarization of the incident light.

The underlying mechanism relies on photoinduced mechanical strain: upon *trans*–*cis* isomerization, the azobenzene units “shorten” by ∼3–5 Å along their molecular axis, thereby perturbing the orientational order of the LCNs and inducing macroscopic contraction along the director [13, 45, 296]. Due to a light absorption gradient across the film thickness, contraction is stronger near the illuminated surface, causing the entire film to bend toward the light source in uniformly aligned samples [296, 297, 298]. The directional bending illustrated in Figure 13b originates from polydomain alignment, which, although producing weaker overall photomechanical response, introduces an additional degree of control: the control of the bending direction by light polarization [45, 69, 299].

By tailoring the director orientation, a wide range of complex deformation modes can be realized, including twisting and coiling [300, 301], accordion‐like actuation [302], and out‐of‐plane buckling [303, 304]. Importantly, these movements can be driven via either photochemical or photothermal pathways, the latter being typically dominant if push–pull azobenzenes are used [287, 305]. In the context of soft robotics, both light‐driven actuation mechanisms offer unique and complementary advantages [287, 305].

Moving toward demonstrations of light‐driven soft robotics, much of the foundational work was again pioneered by the Ikeda group [70, 284, 285, 296, 306, 307]. In a remarkable early example, they fabricated a bilayer LCN strip that acted as a robotic arm, whose movement could be precisely controlled through both spatial localization and wavelength of the incident light (Figure 13c) [284, 285]. In 2008, the same group reported an even more sophisticated example: a pulley‐like system that under dual‐wavelength irradiation underwent continuous rotation, functioning as a light‐fueled plastic motor (Figure 13d) [70]. Such wireless, battery‐free actuators, requiring only light beams for operation, exemplify the potential of azobenzene‐based materials in soft robotics, where self‐sustained, autonomous motion can be achieved under constant optical excitation [70, 308, 309, 310]. Other recent demonstrations have further expanded the scope of azobenzene‐based LC systems, demonstrating, for example, light‐driven gripping devices (Figure 13e) [68, 311], photocontrol over liquid flows [312], light‐responsive cilia arrays [313, 314], and photochemically induced propulsion of biomimetic swimmers [315].

Also, the theoretical framework has been refined to predict how a given illumination (intensity and wavelength) will result in the deformation of azobenzene‐containing polymer networks [206, 221, 281, 316, 317, 318, 319, 320]. For instance, Toshchevikov and Saphiannikova developed static and full kinetic models for photodeformation that incorporate both orientational order and rubber elasticity (including LC interactions) to explain why some azobenzene‐containing elastomers expand while others contract along the polarization direction depending on their molecular architecture [316, 317, 318, 319, 320]. Indeed, experiments have shown “opposite” photoinduced strain in some cases: certain materials elongate in the light polarization or illumination direction, others shrink, likely due to differences in how the polymer backbone is coupled to the azo motion [273, 321, 322]. Understanding these nuances is important for designing materials that respond in desired ways.

It is worth noting that even if photoinduced 3D motions are predominantly studied in LCNs, even non‐liquid‐crystalline, amorphous azopolymers can show photomechanical bending if a gradient of light is applied [276, 323, 324]. For example, if one side of a polymer strip (Kapton) is coated with an azobenzene dye, illumination causes that side to contract relative to the other, bending the strip (Figure 13f) [286, 324]. The magnitude of bending is smaller than in LC systems, but still observable [45, 289, 323, 324]. Furthermore, by combining photomechanical strain with other effects, multi‐responsive actuators have been made (Figure 13g) [287, 325, 326, 327]. Lastly, photochemically controlled hydrogels with localized swelling/shrinking under light irradiation also enable light‐controlled shape morphing [328, 329, 330] as well as, for example, valves for fluid flow control in microfluidic chips [331, 332].

#### 2.5.2 Solid‐to‐Liquid or Solid‐to‐Soft Transitions

Another important phenomenon is photoinduced solid‐to‐liquid (this is real photofluidization) or solid‐to‐soft transitions in selected azobenzene material platforms, including molecular glasses, crystals, and polymeric materials [55, 129, 299, 333, 334]. In these systems, the densely packed *trans* isomers generally form an ordered solid‐state architecture stabilized by efficient molecular packing and intermolecular interactions [55, 129, 299, 333, 334]. Upon UV irradiation, *trans*–to–*cis* photoisomerization introduces a pronounced molecular shape change that disrupts local packing order, weakens intermolecular cohesion, and increases free volume and molecular mobility. As illustrated in Figure 14a, the accumulation of *cis*‐rich disordered domains progressively transforms the material from a rigid solid into a liquid‐like or mechanically softened state, demonstrating how molecular‐scale photoisomerization propagates through mesoscopic packing rearrangements to generate a macroscopic phase transition. Akiyama and Yoshida synthesized a sugar‐alcohol derivative bearing multiple azobenzene arms that is a crystalline solid in the dark but becomes a liquid upon UV exposure because *E*‐to‐*Z* isomerization disrupts molecular packing [335, 336]. Under visible light, the material recrystallizes through *Z*‐to‐*E* isomerization, as shown in Figure 14b [336]. This single‐component system functions as a light‐reversible meltable glue and illustrates how molecular switching can be amplified into a bulk phase transition [335, 336]. Azobenzene‐containing block copolymers provide a second example: *trans–cis* isomerization lowers the *T_g_* of the azobenzene‐bearing block from about 54°C to about 1°C, enabling photoplastic self‐assembly [337]. As a result, *cis*‐cast block copolymer films form strongly segregated cylindrical microdomains at about 40°C, far below the 160°C annealing temperature required for *trans*‐cast films (Figure 14c) [337]. Related studies by Iyoda, Yu, and others show that azobenzene photoalignment can guide phase‐separated block‐copolymer architectures, thereby connecting molecular switching to nanoscale domain organization and release behavior [173, 338, 339].

**FIGURE 14:**
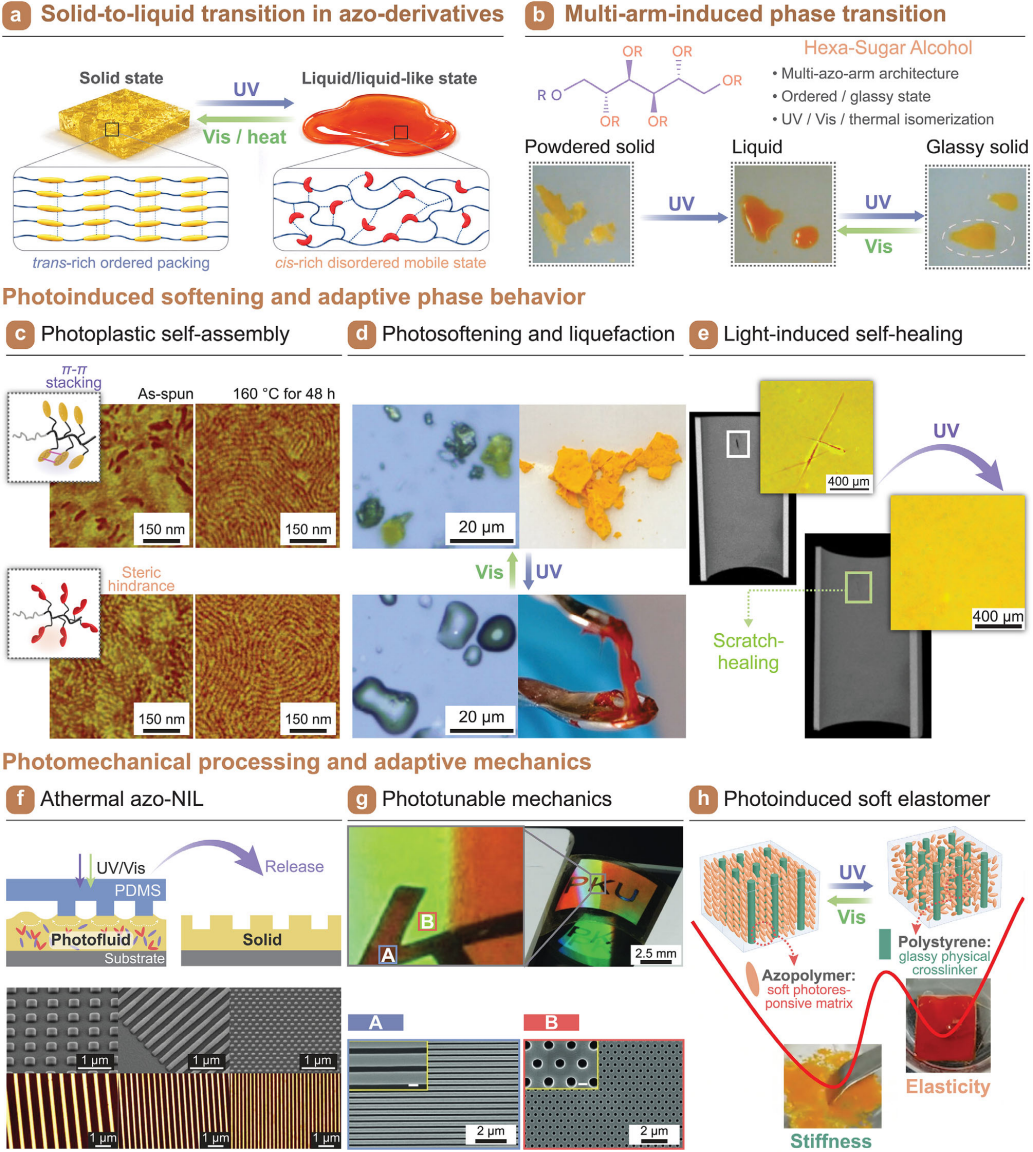
Solid‐to‐liquid phototransition. (a) Schematic illustration of photoinduced solid‐to‐liquid transition in azobenzene‐containing materials via disruption of ordered molecular packing. (b) Chemical structure of azobenzene derivative with hexa‐sugar alcohols and phase transition upon light stimuli. Reproduced with permission [336]. Copyright 2012, Wiley‐VCH. (c) Surface morphologies of azobenzene‐containing block copolymer films annealed in the *trans* state at 160°C and in the *cis* state at 40°C, illustrating the temperature‐dependent microphase organization induced by photoisomerization. Reproduced with permission [337]. Copyright 2023, American Chemical Society. (d) *Trans*‐to‐*cis* photoisomerization lowers the glass‐transition temperature below room temperature, converting the *trans‐*rich glassy state into a *cis‐*rich liquid‐like state. Reproduced with permission [54]. Copyright 2017, Springer Nature. (e) Photoinduced repair before and after UV irradiation of azopolymer inner‐coated pipe. Reproduced with permission [340]. Copyright 2023, Elsevier Ltd. (f) Athermal azobenzene‐based NIL (azo‐NIL) using an azobenzene molecular glass. SEM and AFM images of nanopatterns imprinted into films of azo1. Reproduced with permission [226]. Copyright 2016, Wiley‐VCH Verlag GmbH & Co. KGaA, Weinheim. (g) Athermal and soft multi‐nanopatterning of PM6AZC4 azopolymers. Reproduced with permission [225]. Copyright 2019, Wiley‐VCH Verlag GmbH & Co. KGaA, Weinheim. (h) Light‐enabled mechanically mutable ABA triblock copolymer containing a photoresponsive azopolymer midblock and polystyrene end blocks. Reproduced under the terms of the Creative Commons Attribute 4.0 International License [341]. Copyright 2022, AAAS.

A particularly useful class of side‐chain azopolymers undergoes reversible liquefaction or strong softening under UV irradiation. These systems typically combine azobenzene chromophores with long alkyl spacers and terminal chains [54, 342, 343]. Nearly complete conversion of *trans* azobenzene to the bent *cis* form can lower the *T_g_* below room temperature, converting a *trans*‐rich glassy solid into a *cis*‐rich liquid‐like or highly softened state (Figure 14d) [54]. This phototunable *T_g_* provides a direct route to repairable coatings and light‐assisted healing, as illustrated by scratch‐healing azopolymer coatings in Figure 14e [340].

These photoinduced solid‐to‐liquid or solid‐to‐soft transitions have also enabled mechanically assisted processing routes that go beyond photomelting and healing [54, 225, 334]. A representative example is athermal azobenzene‐based NIL (azo‐NIL), in which an azobenzene molecular glass is patterned at room temperature by placing a transparent stamp in contact with the film and irradiating it with UV/vis light (Figure 14f) [226]. Unlike conventional thermal NIL, this process does not require heating above *T_g_* or high‐pressure imprinting; repeated *trans–cis–trans* isomerization lowers the effective viscosity of the azo material, allowing capillary filling of the stamp cavities and faithful replication of nanoscale patterns. This strategy was further advanced in PM6AZC4 azopolymers, where UV irradiation switches the material from a glassy plastic to a viscoplastic state, whereas visible irradiation partially restores the mechanically hardened *trans*‐rich state (Figure 14g) [225]. The phototunable *T_g_* and reversible nanomechanical response permit regionally selective multiple nanopatterning on flexible polyethylene terephthalate (PET) substrates, yielding structural‐color patterns through sequential athermal NIL and photolithography. More recently, this concept was expanded from viscoplastic azopolymer films to mechanically mutable ABA triblock copolymers, in which a photoresponsive azopolymer midblock provides light‐tunable softness while polystyrene end blocks act as physical crosslinking domains (Figure 14h). In the *trans*‐rich state, the triblock copolymer behaves as a stiff plastic; after UV irradiation, the *T_g_* of the azopolymer midblock drops below room temperature, converting the material into a soft elastomer with skin‐like mechanical compliance [340, 341]. Together, these examples show that azobenzene photoisomerization can regulate not only molecular packing and phase state, but also processing mechanics, enabling reversible transitions among solid, liquid‐like, viscoplastic, and elastomeric states for nanoimprinting, self‐healing, flexible patterning, and mechanically adaptive photonic/electronic platforms.

In all these cases, photon energy is converted into mechanical or structural change through azobenzene isomerization. The relevant output is not merely a change in molecular geometry, but a shift in packing, mobility, modulus, viscosity, or phase state that can be used for repair, processing, pattern transfer, or adaptive interfaces. This phase‐transition route therefore complements the orientation‐stress route discussed above and provides an additional pathway from molecular photoswitching to material‐level function [55, 206].

### 2.6 Summary of Fundamental Photoresponse Mechanisms

From the above sections, we see that the fundamental molecular event (i.e., azobenzene's *trans*–*cis* photoisomerization) can lead to a hierarchy of effects: (i) reorientation of chromophores and polymer backbones (photoinduced anisotropy), (ii) plasticization and mass transport (surface pattern formation), (iii) bulk mechanical deformation (bending/strain), and (iv) photoinduced phase transitions [3, 12, 24, 55, 206]. These effects are often interconnected. For example, the formation of SRGs requires both anisotropic reorientation of chromophores and sufficiently large light‐induced stress to create directional plastic deformations [206, 221, 222, 223, 224, 255]. Similarly, the bending of azobenzene‐containing LCNs often involves penetration depth‐dependent contraction along the nematic director, which is itself set by anisotropic molecular orientation [45, 289, 296, 298].

A crucial point is that light provides spatiotemporal, spectral, and polarization control over these coupled processes. Figure 15 makes this hierarchy explicit by placing *E/Z* photoswitching at the center and linking it outward to molecular tuning parameters, primary property outputs, collective material responses, and application families. The same hierarchy motivates the function‐first organization of section 3, where the application space is classified not by material platform alone, but by the dominant device‐level output: information and wavefront control, energy and heat, adaptive optics, mechanical motion, transport, and bioadaptive interfaces.

**FIGURE 15:**
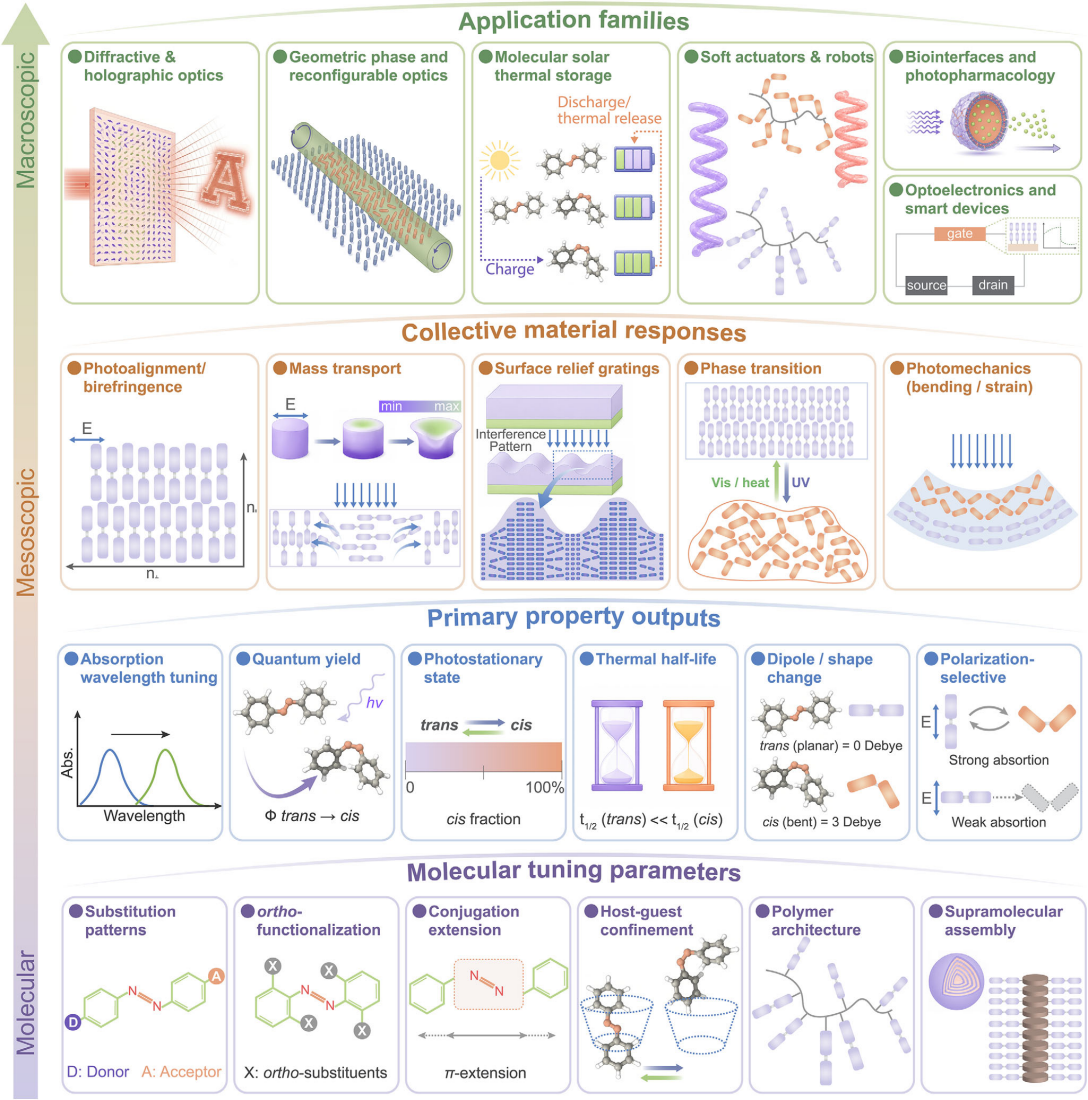
Cross‐scale map of azobenzene optics and photonics. *E/Z* photoswitching is placed at the center and connected to four levels of design and function: molecular tuning parameters, primary property outputs, collective material responses, and application families. The map summarizes the structure‐property‐function logic used throughout this review.

Before moving to applications, we emphasize that modern experiments and simulations increasingly bridge these scales: single‐molecule force spectroscopy and ultrafast spectroscopy [80, 83, 104, 105, 151, 152, 153] quantify the primary molecular event, while molecular dynamics, continuum mechanics, and VPA models connect molecular orientation to stress, flow, and deformation [206, 221, 222, 223, 224, 272, 273, 316, 317, 318, 319, 320]. This cross‐scale understanding is essential because the same photoswitch can serve different device functions depending on how it is embedded, organized, and coupled to a host matrix. Figure 16 and Table 2 therefore serve as a roadmap for the application section.

**FIGURE 16:**
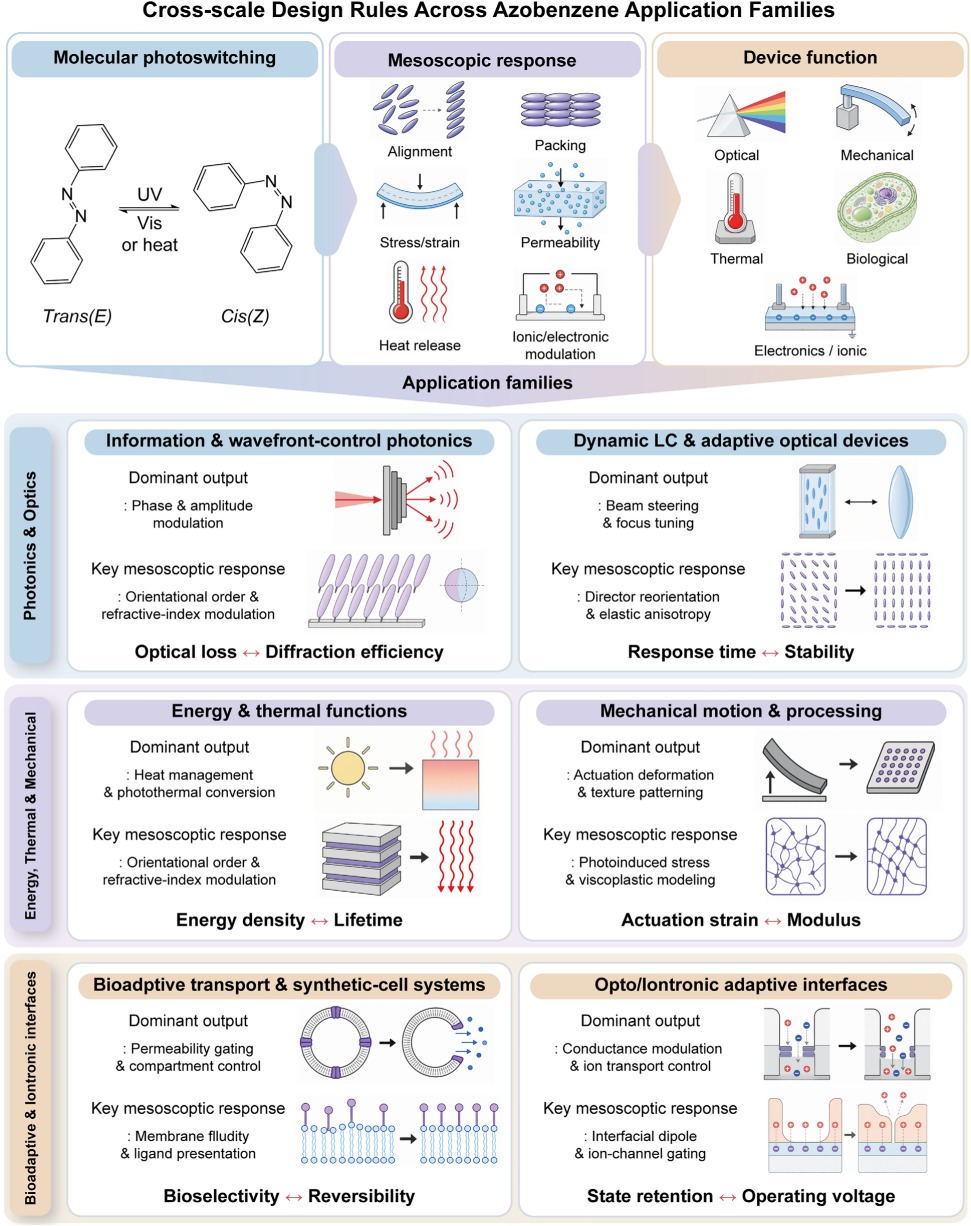
Cross‐scale design rules across function‐based classification of azobenzene applications within a cross‐scale structure‐property‐function framework. The top row summarizes the common structure‐property‐function pathway: molecular *E/Z* photoswitching is converted into mesoscopic material responses such as alignment, packing, stress/strain, permeability, heat release, or ionic/electronic modulation, which in turn produce optical, thermal, mechanical, biological, and iontronic/electronic device functions. The six application families are compared by their dominant output and key mesoscopic response: (1) information and wavefront‐control photonics, (2) dynamic LC and adaptive optical devices, (3) energy and thermal functions, (4) mechanical motion and processing, (5) bioadaptive transport and synthetic‐cell systems, and (6) opto/iontronic adaptive interfaces. Each family also highlights the representative trade‐off that must be optimized: optical loss versus diffraction efficiency, response time versus stability, energy density versus lifetime, actuation strain versus modulus, bioselectivity versus reversibility, and state retention versus operating voltage.

**TABLE 2:** Function‐based classification of azobenzene applications within a cross‐scale structure‐property‐function framework.

Application family	Dominant device output	Material response used	Representative implementations/design priorities
Information and wavefront‐control photonics	Optical phase, diffraction, polarization, holography, optical memory	Photoalignment, birefringence, SRGs, OFSs, LC director patterns	Holographic data storage [33, 67, 261, 344], PB/geometric‐phase optics [35, 59], PVGs [34, 62, 63], OFSs [57, 58], metasurfaces [36], encryption; [345, 346, 347] prioritize optical loss [60, 348, 349], birefringence [3, 31, 164, 165], diffraction efficiency [28, 350, 351, 352, 353], and rewritability [26, 33, 261, 354, 355].
Dynamic LC and adaptive optical devices	Tunable transmittance, reflection, retardation, scattering, display contrast	Azobenzene‐doped LC order changes, Cholesteric LC pitch modulation, photoalignment layers	Smart dimmers [356, 357, 358], reflective filters [359, 360, 361, 362, 363], AR/VR waveguides [34, 64, 364, 365], photoaddressed LC lenses [366, 367, 368], switchable shutters; [358, 369, 370, 371, 372] prioritize low dopant loading [361, 373, 374, 375], contrast [356, 357, 376, 377], response time [26, 378, 379, 380], and device stability [381, 382, 383].
Energy and thermal functions	Stored solar energy and on‐demand heat release	Metastable Z‐isomer enthalpy, packing/cooperative storage, catalytic or optical discharge	MOST fuels [5, 384, 385, 386], polymer/nanocarbon templates [387, 388, 389, 390], phase‐coupled storage; [30, 391, 392, 393] prioritize energy density [385, 386, 394, 395], solar overlap [77, 396, 397, 398], Z lifetime [56, 114, 390, 395], fatigue resistance [30, 384, 389, 395], and controlled release [30, 56, 399, 400].
Mechanical motion and processing	Shape change, actuation, pattern transfer, healing, conformal processing	LCN/LCE contraction, light‐induced stress, directional mass transport, photosoftening, *T_g_* switching	Soft robots [68, 70, 401, 402, 403, 404], grippers [68, 287, 405] motors [45, 70, 310], microfluidic valves [312, 406] azo‐NIL [225, 226], multi‐nanopatterning [195, 214, 225, 262, 407], repairable coatings; prioritize force [45, 53, 408, 409], strain [45, 410, 411, 412], speed [413, 414], penetration depth [299, 415, 416], modulus [417, 418, 419], and cycling [54, 231, 340, 420].
Bioadaptive transport and synthetic‐cell systems	Cargo release, pore gating, permeability control, biochemical compartmentalization	Membrane packing changes, pore‐size switching, DNA‐pore opening/closing, host‐guest regulation	Drug delivery [72, 421, 422, 423, 424], photopharmacology [19, 425], light‐gated membranes [421], DNA nanopores, synthetic cell microreactors; prioritize biocompatible wavelengths, aqueous stability, selectivity, and reversibility [19, 72, 421, 425].
Opto/iontronic adaptive interfaces	Conductivity, mobility, resistance, synaptic or memory states	Dipole/polarity changes, ionic transport modulation, dielectric switching, morphology change	Ionic skins [426], light‐responsive ionic films [233, 427], phototransistors [428, 429], molecular/optical memory [430], neuromorphic devices; [431, 432, 433] prioritize state retention [434], endurance [426, 433, 435], low‐voltage operation [427, 433], and integration with soft substrates [426].

## 3 Application Families Organized by Dominant Device Function

Azobenzene applications can be classified by molecular scaffold, host matrix, optical input, or device geometry. For the present cross‐scale Review, however, the most useful organizing criterion is the dominant device‐level function delivered by the material. This function‐first classification follows the central structure‐property‐function question of the manuscript: how does molecular *E/Z* photoswitching propagate into mesoscopic responses such as photoalignment and birefringence, [3, 31] stress, mass transport [3, 12, 199], LC‐order modulation [26, 436], heat release [5, 30, 77], or permeability gating [71, 72, 437], ionic/electronic modulation [426, 438, 439, 440] and how are these responses converted into adaptive devices? Figure 16 and Table 2 summarize this logic by grouping applications into six families: [5, 26, 33, 55, 71, 426] (i) information and wavefront‐control photonics [3, 26, 28, 31, 33, 34, 35, 36, 57, 58, 59, 60, 62, 63, 67, 164, 165, 261, 344, 345, 346, 347, 348, 349, 350, 351, 352, 353, 354, 355], (ii) dynamic LC and adaptive optical devices, [26, 34, 64, 356, 357, 358, 359, 360, 361, 362, 363, 364, 365, 366, 367, 368, 369, 370, 371, 372, 373, 374, 375, 376, 377, 378, 379, 380, 381, 382, 383] (iii) energy and thermal functions, [5, 30, 56, 77, 114, 384, 385, 386, 387, 388, 389, 390, 391, 392, 393, 394, 395, 396, 397, 398, 399, 400], (iv) mechanical motion and processing [45, 53, 54, 68, 70, 195, 214, 225, 226, 231, 262, 287, 299, 310, 312, 340, 401, 402, 403, 404, 405, 406, 407, 408, 409, 410, 411, 412, 413, 414, 415, 416, 417, 418, 419, 420], (v) bioadaptive transport and synthetic‐cell systems [72, 421, 422, 423, 424], photopharmacology [19, 72, 421, 425], and (vi) opto/iontronic adaptive interfaces [233, 426, 427, 428, 429, 430, 431, 432, 433, 434, 435].

This organization also separates design problems that are often mixed in azobenzene literature. In information photonics, the desired output is optical phase, diffraction, polarization conversion, holography, or optical memory. In adaptive LC devices, the target is tunable transmission, reflection, retardation, scattering, or display contrast. In MOST systems, the device function is stored enthalpy and on‐demand heat release. In mechanical systems, the output is shape change, actuation, pattern transfer, healing, or conformal processing. In bioadaptive systems, the function is transport, pore gating, permeability control, or biochemical compartmentalization. In opto/iontronic interfaces, the output is conductivity, mobility, resistance, synaptic weight, or memory state. The same azobenzene photoswitch may contribute to more than one family, but the dominant output determines the relevant molecular and materials‐design priorities.

### 3.1 Information Processing and Wavefront‐Control Photonics

The earliest device‐oriented azobenzene photonic applications emerged from photoinduced birefringence, polarization holography, and SRG formation. In these systems, azobenzene is used as a rewritable optical information medium: polarized or structured light writes molecular orientation or surface topography into the film, and the recorded anisotropy or relief modulates phase, diffraction, polarization, and holographic reconstruction. Figure 17a illustrates the classical route based on interference writing of azopolymer films, where grating formation produces diffracted readout orders and can be erased or rewritten by subsequent optical exposure [33, 58]. This platform established the connection between molecular reorientation or mass transport and optical memory.

**FIGURE 17:**
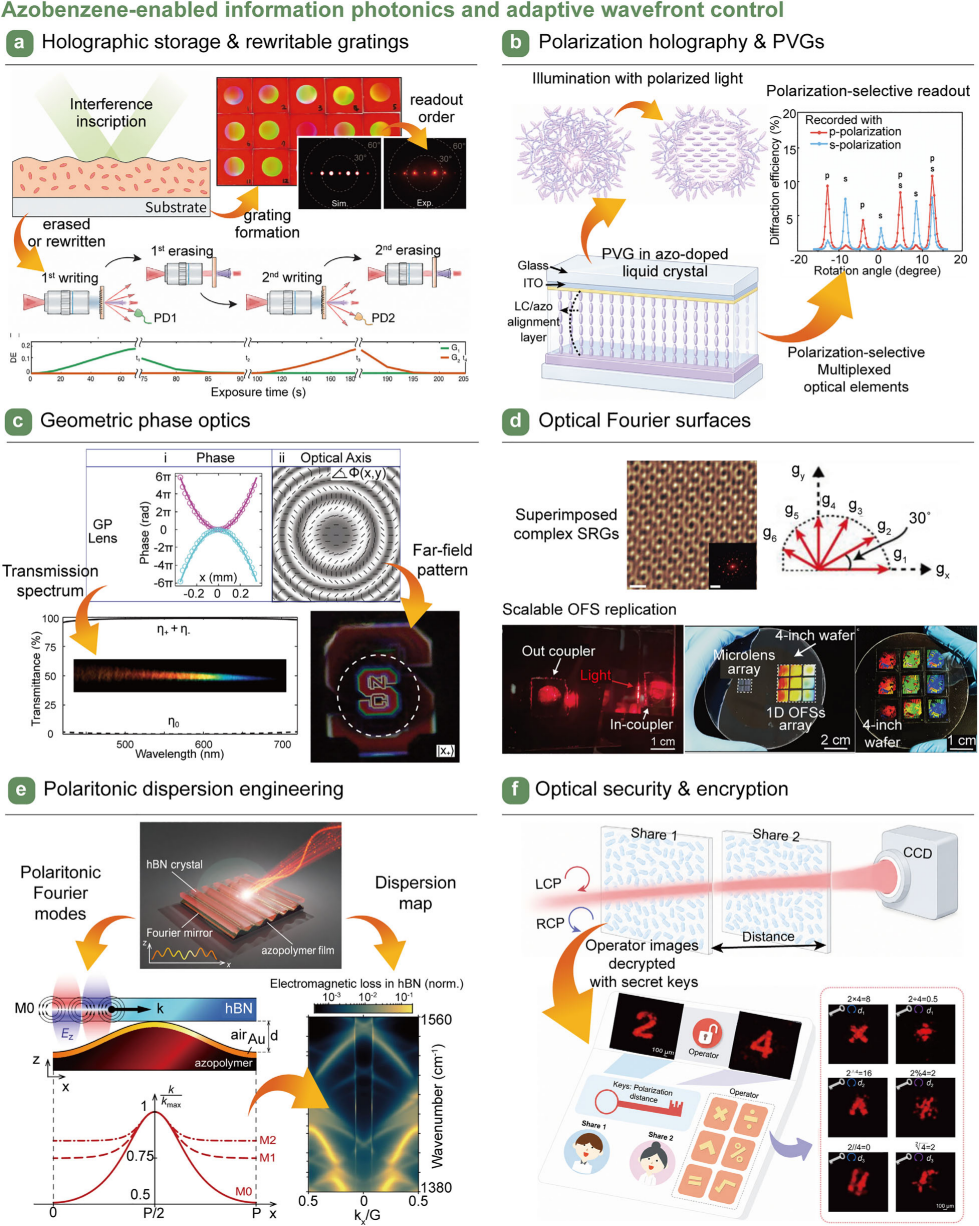
Information processing and wavefront‐control photonics enabled by azobenzene materials. (a) Holographic data storage and rewritable diffraction gratings based on interference writing, SRG/volume‐grating formation, diffractive readout, and optical write‐erase‐rewrite cycles. Adapted with permission [58]. Copyright 2021, Wiley‐VCH GmbH (top panel); adapted under the terms of the Creative Commons Attribution 4.0 International License [260]. Copyright 2021, The Authors, published by Wiley‐VCH GmbH, Advanced Materials Interfaces (bottom panel). (b) Polarization holography and PVGs, where polarization‐selective photoalignment or azo‐doped LC ordering produces polarization‐dependent diffraction and multiplexed optical elements. Adapted with permission [33, 441]. Copyright 2001, Wiley‐VCH Verlag GmbH, Weinheim, Fed. Rep. of Germany (top left panel); Copyright 2010, The Society of Polymer Science, Japan (top right panel). (c) Geometric phase holograms with arbitrary wavefronts, enabling broadband operation high reconstruction efficiency. Adapted under the terms of the Creative Commons Attribution 4.0 International License [35]. Copyright 2015, Optical Society of America. (d) Optical Fourier surfaces (OFSs), where superimposed SRG components encode a target grating‐vector spectrum into one azopolymer relief, scalably replicated into waveguide couplers and microlens or 1D OFS arrays. Reproduced under the terms of the Creative Commons Attribution 4.0 International License [57, 199]. Copyright 2022, The Authors, published by Wiley‐VCH GmbH, Laser & Photonics Reviews (top panel); Copyright 2024, The Authors, published by Wiley‐VCH GmbH, Advanced Materials (bottom right panel); Reproduced with permission [58]. Copyright 2021, Wiley‐VCH GmbH (bottom left panel); (e) Polaritonic Fourier surface formed by smooth azopolymer OFSs that harmonically tunes phonon‐polariton momentum in a van der Waals crystal (e.g., hBN), yielding a clean Bloch band structure and bandgap. Reproduced under the terms of the Creative Commons Attribution 4.0 International License [442]. Copyright 2025, The Authors, published by Springer Nature, Nature Communications. (f) Information security, encryption, and optical communication using polarization selectivity (left‐handed circularly polarized (LCP)/right‐handed circularly polarized (RCP) beams), distance multiplexing, and key‐based optical decryption. Adapted under the terms of the Creative Commons Attribution 4.0 International License [347]. Copyright 2024, The Authors, published by Institute of Optics and Electronics, Chinese Academy of Sciences.

A second information‐photonic route uses polarization holography and PVGs. As summarized in Figure 17b, [33, 441] counterpropagating or interfering circularly polarized beams write a periodic director distribution into an azobenzene‐containing or azobenzene‐photoaligned LC medium. On readout, the handedness of the incident circular polarization selects the diffraction order, enabling polarization‐selective beam steering and multiplexed optical elements [33, 34, 61, 350, 352, 353, 441, 443]. Figure 17c shows the related but now widely used Pancharatnam‐Berry (PB) or geometric‐phase concept: a spatially varying photoalignment pattern imposes an in‐plane optical‐axis distribution, which is converted by the LC layer into a spin‐dependent phase profile for vortex beams, lenses, gratings, and polarization converters [35, 59, 60, 351]. In this case, the azobenzene layer functions as a high‐resolution, rewritable director‐patterning tool, whereas the LC layer supplies the required birefringent phase retardation.

Azopolymer SRGs and OFSs provide a third branch of wavefront‐control photonics. Figure 17d highlights OFSs, where complex surface profiles generated by superimposed or digitally designed SRG components encode the spatial‐frequency content needed to produce focal spots, Bessel or ring beams, letters, or other far‐field patterns [57, 199]. These devices are especially attractive because the same material layer can store optical information as topography and can be erased, overwritten, or replicated. This concept has recently been extended to flat diffractive couplers, optical in/out‐couplers for waveguides, and multifunctional Fourier‐coded surfaces [36, 58, 260, 265, 444].

A practical limitation of azobenzene SRGs is their strong absorption below 550 nm wavelength, which restricts applications across the full visible spectrum. This challenge has been mitigated by replicating azo‐SRG templates into transparent materials via soft‐lithographic molding: Liu, Wang, and coworkers first suggested this direction [445]. Using a polydimethylsiloxane (PDMS) replica, the SRG pattern can be transferred into diverse optical materials, enabling new applications such as light‐trapping layers in solar cells [446], large‐area microlens arrays [192], and bio‐inspired diffusers for enhancing light‐emitting diode (LED) outcoupling [267]. Very recently, Lee et al. demonstrated reusable elastic molds for imprinting high‐refractive‐index (∼2.0), optically transparent organic‐inorganic hybrid resins (TiO_2_‐nanoparticle‐dispersed acrylate) [57], thereby fabricating OFSs or DOEs with full‐color diffraction capability [57]. These molds can be reused multiple times, achieving wafer‐scale throughput (Figure 17d) [57] that far surpasses direct laser inscription on azopolymer films and thus bringing azobenzene‐based photonic components closer to practical deployment [57, 58, 199, 447].

The newest systems move from single gratings toward reconfigurable metasurfaces and adaptive photonics. Figure 17e highlights one azopolymer route, the polaritonic Fourier surface: a smoothly modulated azopolymer OFSs harmonically modulates the momentum of phonon‐polaritons in an adjacent van der Waals crystal (e.g., hBN), and its continuous, edge‐free relief minimizes scattering, producing a well‐defined Bloch band structure and a polaritonic bandgap [442]. Although such polaritonic platforms remain largely at the proof‐of‐concept stage, the significance is that a smoothly programmable SRG template, which, as an azopolymer, is in principle rewritable, converts a fixed polariton dispersion into a harmonically engineered one, extending azopolymer wavefront control from the far field (Figure 17d) into the near‐field polaritonic regime for dispersion engineering and subdiffractional nanolight guiding. Information security and optical communication form a complementary output class, illustrated in Figure 17f [347]. Hidden patterns can be encoded through polarization selectivity, wavelength multiplexing, or multilayer optical channels and decoded only under prescribed optical conditions [345, 346, 347]. Thus, Figure 17 as a whole shows how the same molecular event, *E/Z* photoswitching, can be translated into birefringence, relief, or director states for optical phase, diffraction, polarization, holography, memory, and polariton dispersion.

### 3.2 Dynamic Liquid Crystal and Adaptive Optical Devices

LC systems constitute a distinct application family because they translate molecular photoisomerization into macroscopic optical modulation through the strong orientational response of the LC host. In azobenzene‐based photoalignment layers, linearly polarized UV or visible light induces anisotropic molecular ordering and defines an easy axis at the surface, thereby controlling LC anchoring and director orientation without mechanical rubbing [26, 33, 39, 436]. Figure 18a illustrates this photoalignment principle. Polarized irradiation writes an azimuthal easy axis into the azobenzene alignment film, and the LC director subsequently follows this surface‐imposed orientation through anchoring interactions, enabling noncontact control of azimuthal alignment, anchoring strength, and spatially patterned director fields [436]. The functional separation between the photoresponsive recording layer and the optical LC layer underpins a wide range of LC photonic devices, including patterned retarders, polarization gratings, LC lenses, and display‐compatible optical components [366, 367, 368].

**FIGURE 18:**
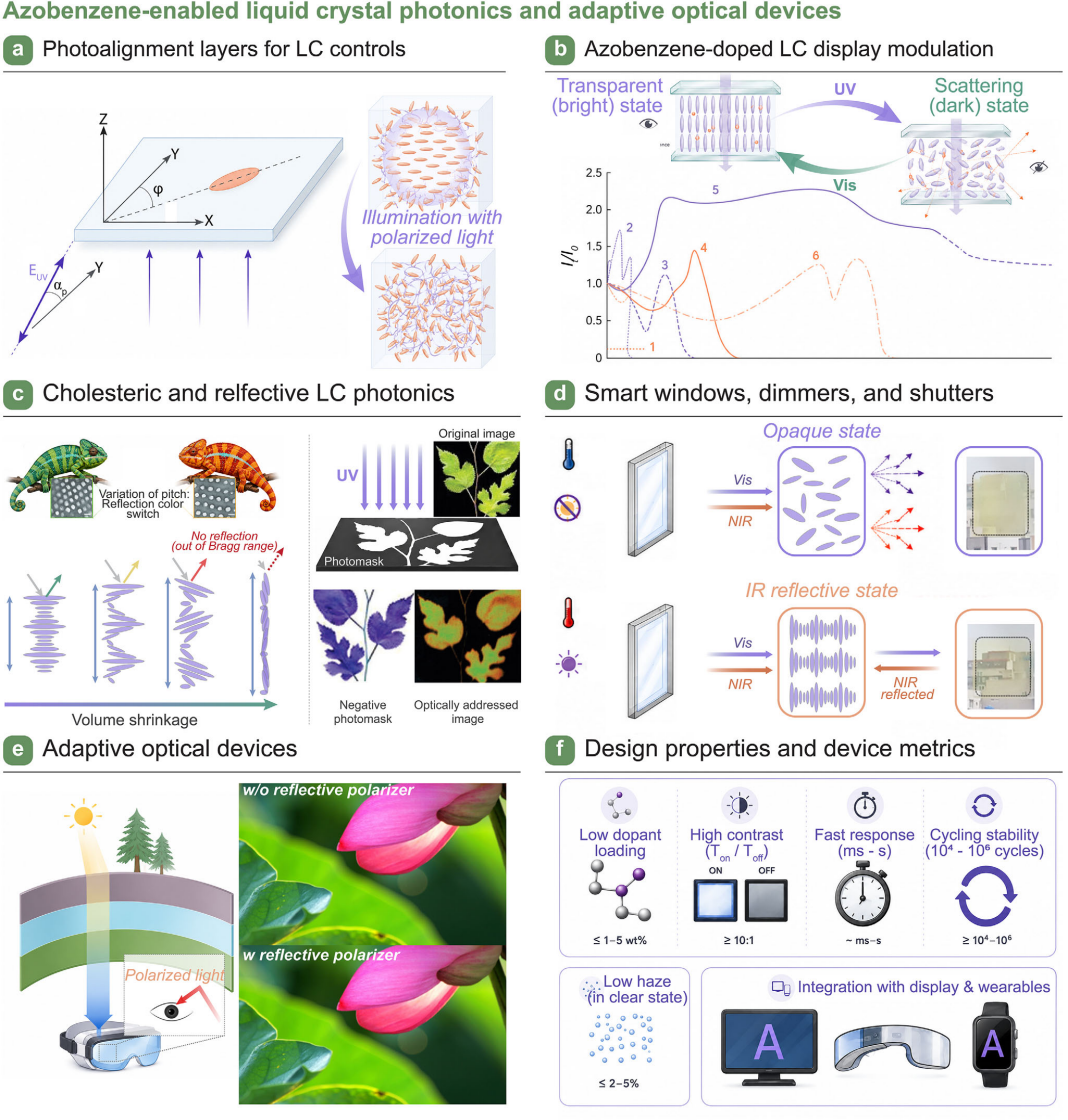
Dynamic LC and adaptive optical devices enabled by azobenzene. (a) Polarized light writes an azimuthal easy axis in an azobenzene alignment film, and the LC director follows this surface‐imposed orientation through anchoring interactions, enabling noncontact control of LC alignment. Adapted with permission [436]. Copyright 2012, American Physical Society. (b) Azobenzene‐doped LC switching and display modulation, in which *trans–cis* isomerization changes LC order, transmission, scattering, and display contrast. Adapted with permission [448]. Copyright 1987, The Chemical Society of Japan. (c) Cholesteric photonics and photoaddressable color patterning. Stimulus‐induced structural changes tune Bragg reflection in chameleon skin and azobenzene cholesteric LCs (left panel), while masked UV irradiation enables spatially resolved reflective color patterns (right panel). Adapted with permission [375, 449]. Copyright 2020, The Royal Society of Chemistry. Copyright 2016, American Chemical Society. (d) Representative azobenzene‐doped LC smart window illustrating switching between scattering (privacy) and IR‐reflective states for adaptive regulation of visible transmission and solar heat gain. Adapted with permission [382]. Copyright 2021, American Chemical Society. (e) Adaptive LC optics for AR/VR systems. An LC‐based tunable‐transmittance film and reflective polarizer dynamically regulate ambient‐light transmission and optical coupling, improving contrast and visibility under varying illumination conditions. Adapted with permission [450]. Copyright 2016, Society for Information Display. (f) Representative design priorities and device metrics, including low dopant loading, high contrast, fast response, cycling stability, low haze, and integration with displays and wearables.

In the 1980s, dichroic azobenzene dyes were used in so‐called guest‐host LCDs, where applying UV could turn the cell dark by disrupting alignment (this didn't become widespread, but was explored for light modulators) [370, 451, 452]. More successfully, Tazuke et al. showed an “amplified image recording” in an LC medium using photochemical phase transitions. In Figure 18b, *trans–cis* photoisomerization of a small amount of azo dopant disrupts LC order and triggers a nematic‐to‐isotropic phase transition, causing a dramatic nonlinear change in optical readout through crossed polarizers [448]. These systems established the principle that a low concentration of molecular photoswitch can modulate a macroscopic optical texture. The relevant design criteria are low dopant loading, high photostationary conversion, rapid recovery, low haze in the clear state, high contrast in the switched state, and fatigue resistance under repeated cycling [26, 448].

Azobenzene‐doped cholesterics have also been used to achieve optically tunable reflection colors. Cholesteric and reflective LC devices add helical pitch as an additional optical degree of freedom, enabling dynamic control of selective reflection, photonic bandgaps, and structural color [375, 449, 453, 454, 455, 456, 457]. Because the reflected wavelength follows the Bragg condition, changes in periodic spacing or cholesteric pitch directly shift the reflected color [359, 360, 361, 362, 363, 372, 373, 374, 376]. Figure 18c illustrates this principle through chameleon skin, where stimulus‐induced changes in periodic photonic nanostructures produce structural color variation. In azobenzene‐containing cholesteric LC systems, an analogous mechanism occurs when photoisomerization of an azobenzene chiral dopant changes its helical twisting power, modifies the cholesteric pitch, and shifts the Bragg reflected wavelength [449]. This mechanism enables optically tunable reflective filters, switchable photonic bandgaps, color‐tunable cells, and smart dimmers. This concept was further demonstrated in photoswitchable axially chiral azobenzene dopant systems reported by Quan Li and coworkers, where masked UV irradiation produced spatially resolved color patterns in a chiral nematic display cell [375]. Building upon this foundation, recent advances have extended the concept beyond the visible spectrum. Qin et al. reported a tristable chiral switch that integrated both azobenzene and *o*–fluoroazobenzene units within a single molecule, enabling piecewise photo tuning of the helical pitch across two adjacent reflection windows, one spanning the visible range (RGB colors) and another extending into the near‐IR. Under sequential irradiation, the cholesteric film effectively realized full‐color (RGB+black) reflective displays [458]. Together, such azobenzene‐based cholesteric systems, from earlier bistable to more recent tristable designs, demonstrate how light‐driven molecular switching can dynamically tune the helical pitch and reflection color across visible and near‐ IR regions, paving the way for reconfigurable reflective displays, full‐color E‐paper, and adaptive camouflage materials.

Azobenzene‐doped LC systems are attractive for smart windows, dimmers, and optical shutters because nanoscale photoisomerization can be converted into macroscopic control over transmittance, haze, and selective reflection. Depending on the LC phase and alignment state, these materials can operate as transparent dimmers, scattering privacy shutters, or wavelength‐selective heat‐regulating windows [357, 358, 369, 382, 459, 460, 461, 462]. Figure 18d shows a representative push–pull azobenzene‐doped LC smart window, where UV or thermal stimuli induce SmA* to N* phase switching, producing a NIR reflective state that blocks solar heat while retaining visible transparency. When the initial LC alignment is modified, the same platform can switch between hazy opaque and IR reflective states, enabling combined privacy and thermal management [382]. More broadly, these examples show how azobenzene LC materials can regulate not only the amount of transmitted light but also its spectral content, scattering state, and viewing background, providing a bridge from smart windows and shutters to adaptive optical systems.

Beyond display modulation and smart window technologies, azobenzene‐enabled LC systems and related photoaligned LC optics have expanded the scope of adaptive optical components that regulate transmission, selective reflection, polarization, and light propagation. Through photoresponsive dopants, azo dye photoalignment, and birefringence patterning, LC devices can adapt their optical response to external stimuli or predesigned director fields, enabling applications ranging from tunable reflectors and optically addressed displays to lenses, beam steering elements, wearable optics, and display‐integrated optical systems [34, 356, 450, 455, 463, 464]. Figure 18e illustrates a representative adaptive‐optics application in AR systems, where a tunable‐transmittance LC film is integrated with a reflective polarizer to dynamically regulate ambient‐light transmission and improve viewing contrast [450]. Figure 18f therefore lists the most important device metrics: low dopant loading to reduce absorption, high optical contrast or transmittance ratio, fast response, long cycling stability, low haze, and compatibility with displays or wearables. Compared with azopolymer SRGs, LC devices often require weaker molecular motion but exploit stronger mesoscopic amplification through LC elasticity and birefringence. They are thus a distinct function family even when they use the same azobenzene photoalignment chemistry as wavefront‐control photonics.

### 3.3 Energy Conversion, Storage, and Thermal Release

MOST materials follow a different application logic from wavefront‐control or LC devices. Here, the metastable *Z* isomer stores photon energy as chemical enthalpy, and the device‐level output is on‐demand heat rather than optical phase or image contrast [465]. Figure 19a presents the general operating principle of a MOST system: solar or artificial illumination charges the *trans/E* form into the higher‐energy *cis/Z* state, and thermal, catalytic, or photochemical triggering releases heat during *Z*‐to‐*E* back‐isomerization [5, 30, 466, 467]. Among the molecular candidates explored for this purpose, azobenzene is particularly attractive owing to its substantial isomer enthalpy difference (∼50 kJ mol^−1^ or ∼0.5 eV per molecule), robust photocyclability with minimal fatigue over extended irradiation cycles, low‐cost synthesis, and exceptional amenability to structural modification [147, 468, 469]. The relevant performance metrics that govern practical MOST implementation include energy storage density, photoisomerization quantum yield, solar‐spectrum overlap, *Z*‐state thermal lifetime, fatigue resistance over repeated charge–discharge cycles, and the ability to release stored heat controllably under mild triggering conditions.

**FIGURE 19:**
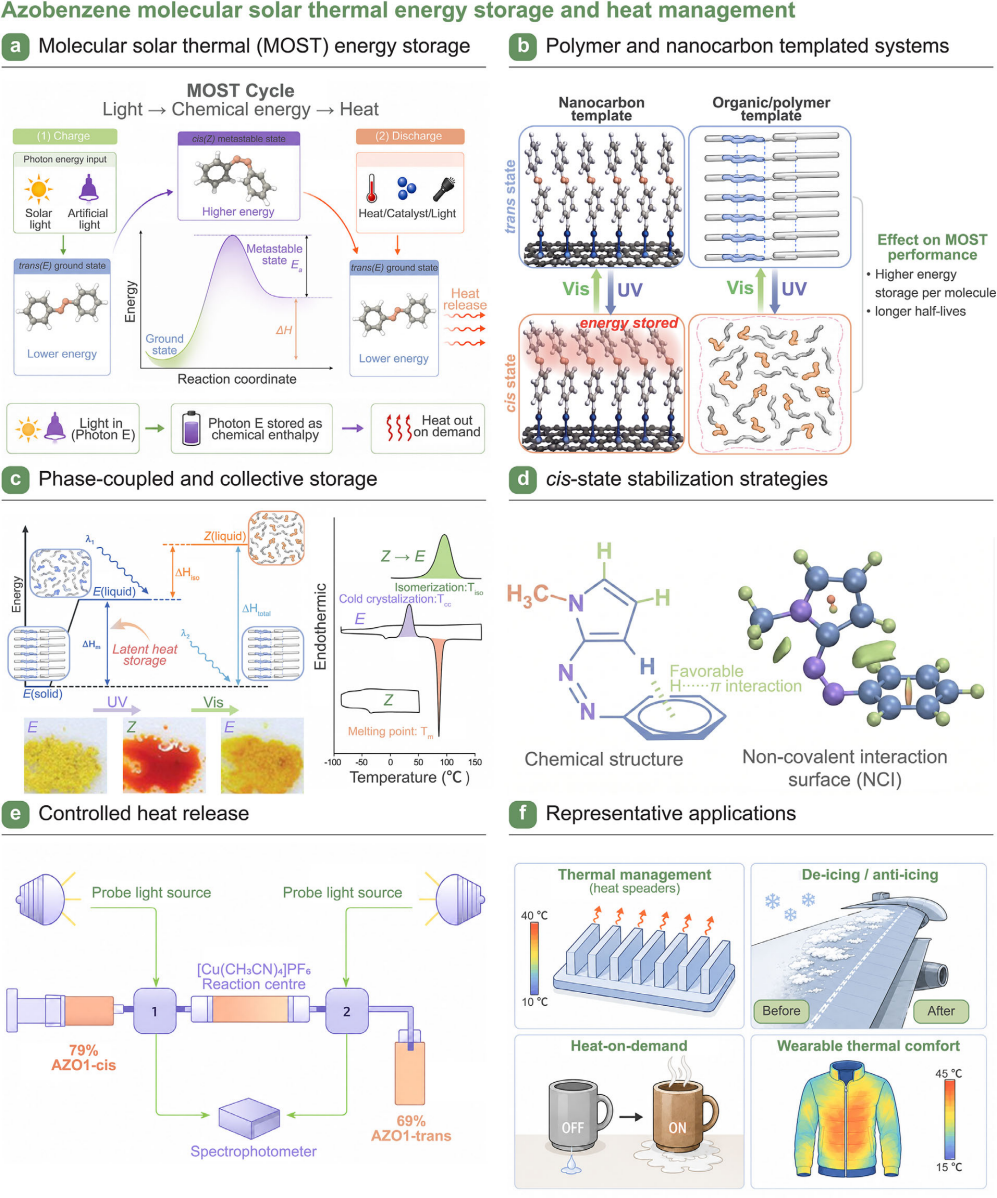
Energy and thermal functions of azobenzene materials. (a) MOST cycle based on light‐driven *E*‐to‐*Z* charging and triggered *Z*‐to‐*E* heat release [5, 30, 466, 467]. (b) Polymer and nanocarbon template systems that improve cooperative packing, storage density, and heat transfer through materials architecture [5, 30, 387, 388, 466, 470, 471, 472]. (c) Phase‐coupled MOST energy storage in azobispyrazoles. Energy is stored through coupled *E*→*Z* photoisomerization and melting, and released upon visible‐light‐driven back‐isomerization and crystallization. Adapted under the terms of the Creative Commons Attribution 4.0 International License [473]. Copyright 2022, The Authors, published by American Chemical Society. (d) Strong intramolecular C–H···*π* interaction between the pyrazole ring and the adjacent phenyl ring stabilizing the *cis* isomer and resulting in an exceptionally long thermal half‐life for arylazopyrazoles. Adapted under the terms of the Creative Commons Attribute 4.0 International License [122]. Copyright 2016, American Chemical Society. (e) A custom‐built microfluidic MOST device where catalytic triggering of the *cis–trans* isomerization achieves on‐demand heat release. Adapted under the terms of the Creative Commons Attribution 4.0 Internation License [384]. Copyright 2019, The Royal Society of Chemistry. (f) Representative thermal applications, including thermal management, de‐icing/anti‐icing, heat‐on‐demand devices, solar‐charging media, reusable thermal devices, and wearable thermal comfort. The lower bars summarize the dominant outputs and mesoscopic responses relevant to energy and thermal functions.

Materials architecture plays an important role in MOST performance. As shown in Figure 19b, polymer and nanocarbon templated systems can regulate photoswitch packing, mobility, thermal transport, molar energy storage density, and cycling through covalent attachment or supramolecular organization [5, 30, 387, 388, 394, 466, 470, 471, 472]. For example, in azobenzene‐nanocarbon systems, computational studies first predicted that nanotube templating could enhance photoswitch packing and molar energy storage density [387, 470]. This concept was later demonstrated experimentally in azobenzene‐functionalized single‐walled carbon nanotubes, where solid‐state packing increased the stored energy per azobenzene from 58 to 120 kJ mol^−1^, and the material maintained robust photochemical and thermal cycling stability. Phase‐directing groups, such as linear or branched alkyl chains, diacetylene units, and H‐bonding groups, can be attached to azobenzene to control noncovalent intermolecular interactions and tune the thermal properties of the system [56, 384, 391, 397, 473]. Typically, the *E* isomer forms a solid, whereas the *Z* isomer remains liquid; consequently, triggering heat release from the *Z* state delivers a total energy output that combines the energy stored in the photoisomerized chemical bonds (Δ*H*_iso_) and the latent heat associated with the phase transition (Δ*H*_m_). As a representative example of this phase‐coupled storage strategy, Figure 19c illustrates an azobispyrazole system in which energy is stored through coupled *E→Z* photoisomerization and melting, and released upon visible‐light‐driven back‐isomerization and crystallization. More broadly, azobenzene is not merely an isolated energy carrier; rather, its energy‐storage and heat‐release behavior is governed by the surrounding matrix or by phase‐directing groups that regulate molecular packing, mobility, and heat release [473].

Another key parameter governing MOST performance is the *t*_1/2_ of the *cis* isomer. A longer storage time is highly desirable for practical applications [474]. Pristine azobenzene exhibits a *t*_1/2_ of approximately 4.3 days, whereas tetra‐*ortho*‐fluorinated azobenzene shows a *t*_1/2 _of about 700 days [114, 117] at 25°C, representing a significant improvement over the pristine system. Fuchter and coworkers reported an arylazopyrazole derivative with an exceptionally long *t*_1/2_ of approximately 1000 days [122]. They discovered that the presence of a C─H bond on the pyrazole ring, positioned *ortho* to the azo group, enables an intramolecular C─H···π interaction with the adjacent phenyl ring, forming a T‐shaped *cis* isomer (Figure 19d) [122]. This noncovalent interaction effectively stabilizes the *cis* isomer, significantly extending the lifetime of the metastable state and resulting in prolonged heat storage capability.

Controlled heat release represents a central challenge in practical MOST implementation. In 1938, Hartley reported that adding a few drops of acetic acid significantly accelerated the *cis* to *trans* conversion, completing the back‐isomerization within seconds [10]. This acceleration was attributed to resonance stabilization of the azonium ion, which weakens the azo double bond and becomes more pronounced when electron‐rich substituents are conjugated to the azo linkage [475]. In 2019, Moth–Poulsen and coworkers identified two efficient metal catalysts, cobalt(II) phthalocyanine and [Cu(CH_3_CN)_4_]PF_6_, that promote *cis* to *trans* isomerization within their custom‐built microfluidic MOST device (Figure 19e) [384]. More recently, Greenfield, Fuchter, and Han demonstrated that arylazopyrazole motifs undergo efficient redox‐induced *cis* to *trans* switching in both solution and condensed phases [476]. In this system, a catalytic amount of charge drives the redox process, enabling *cis* to *trans* conversion through an electrochemical pathway. Such electrochemical switching offers a mild and controllable method for heat release, particularly under low‐light conditions or when the PSS does not favor the trans isomer. Collectively, these results indicate that no single trigger mechanism simultaneously maximizes energy density, release rate, operational simplicity, and integration capability, highlighting the importance of application‐specific trigger selection in future MOST technologies. Figure 19f summarizes representative applications, including de‐icing/anti‐icing, heat‐on‐demand devices, and wearable thermal comfort.

### 3.4 Mechanical Motion, Soft Robotics, and Mechanically Enabled Processing

Mechanical applications can be divided into two linked categories: systems that convert light into motion and systems that use light‐tunable mechanics for processing, repair, or conformal fabrication [12, 70, 337, 341, 477, 478, 479, 480, 481, 482]. Figure 20a summarizes LCN/LCE photomechanics, where azobenzene *E*‐to‐*Z* switching perturbs LC order and produces contraction along the director [24, 25, 45, 289, 320, 408, 409, 410, 411, 418, 419, 483]. The magnitude and mode of this response are governed by chromophore concentration, network architecture, backbone rigidity, LC order, and photomechanical coupling between molecular switching and elastic deformation [44, 45, 296, 298, 300, 301, 302, 303, 304, 308, 313]. The importance of LCNs/LCEs is that they amplify nanoscale isomerization into millimeter‐scale motion through LC elasticity and network anisotropy [12, 25, 289, 320, 484].

**FIGURE 20:**
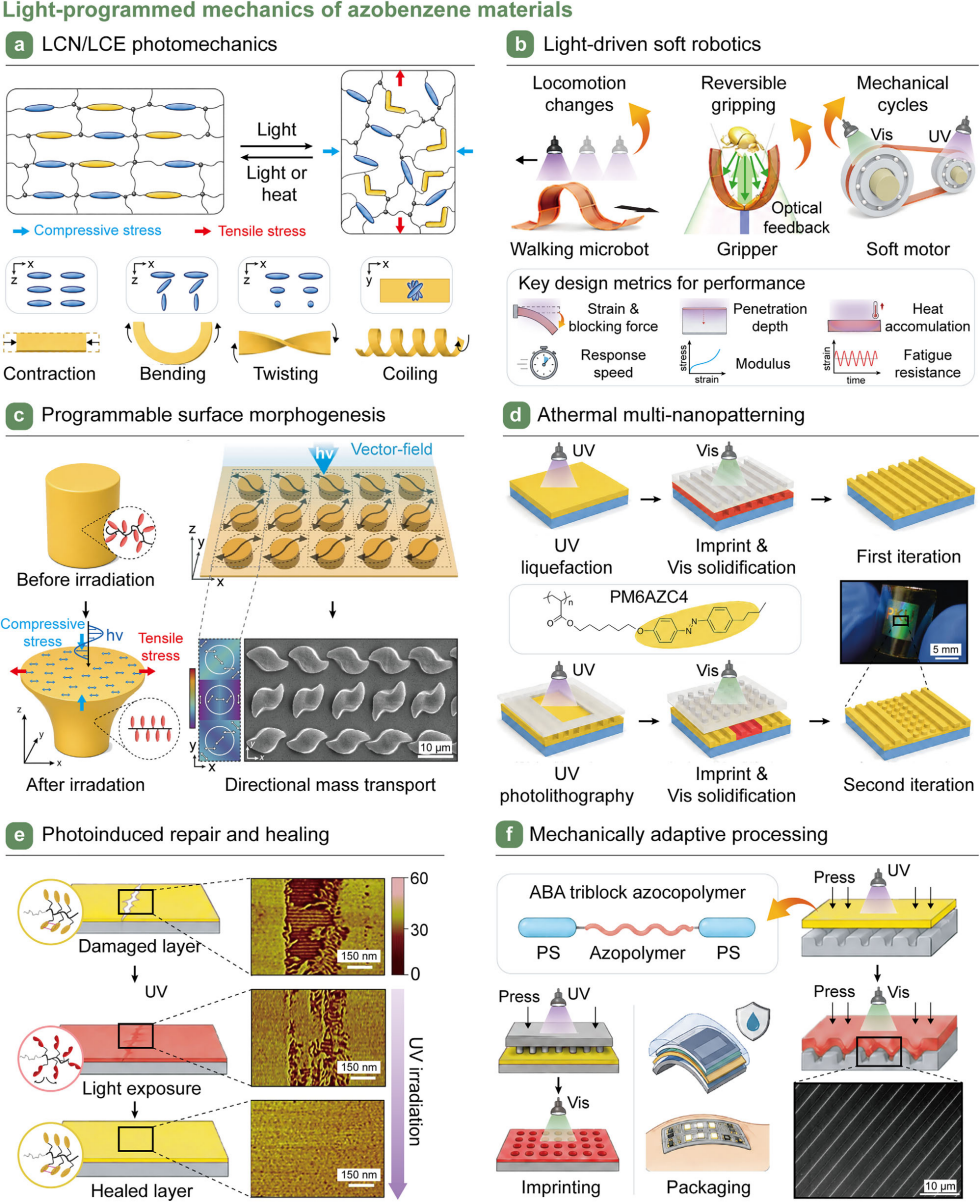
Mechanical motion and mechanically enabled processing in azobenzene materials. (a) Liquid‐crystalline polymer network (LCN)/liquid crystal elastomer (LCE) photomechanics, in which light‐driven director reorientation and LC‐order reduction generate contraction, bending, and twisting [44, 45, 302, 308, 313]. (b) Light‐driven walking actuators, grippers, and mechanical systems driven by global, patterned, or vectorial light fields, with key actuation metrics summarized below [68, 70, 302, 308, 313, 485]. Adapted with permission [68, 70, 485]. Copyright 2017, Wiley‐VCH Verlag GmbH & Co. KGaA, Weinheim; reproduced under the terms of the Creative Commons Attribution 4.0 International License. Copyright 2017, The Author(s); Copyright 2008, Wiley‐VCH Verlag GmbH & Co. KGaA, Weinheim. (c) Directional mass transport and surface reconfiguration, vectorial‐light‐field‐guided deformation, and programmable topography change; these processes link local polarization to local stress and anisotropic material displacement. Adapted under the terms of the Creative Commons Attribution 4.0 International License [220]. Copyright 2026, The Author(s). (d) Athermal NIL and multi‐nanopatterning based on light‐softened azobenzene molecular glasses or phototunable azopolymers. Adapted with permission [225]. Copyright 2020, Wiley‐VCH Verlag GmbH & Co. KGaA, Weinheim. (e) Repairable coatings and self‐healing/solid‐to‐soft transitions enabled by photosoftening, *T_g_* switching, and enhanced chain mobility [54, 55, 337, 340]. Adapted with permission [337]. Copyright 2023, American Chemical Society. (f) Mechanically mutable materials and conformal processing, exemplified by ABA triblock copolymers that switch from stiff plastic to soft elastomeric states at room temperature for nonplanar imprinting and packaging [341]. Reproduced under the terms of the Creative Commons Attribution 4.0 International License [341]. Copyright 2022, The Author(s); exclusive licensee AAAS.

Figure 20b highlights soft actuators, grippers, walking microrobots, bending actuators, and robotics [68, 70, 284, 285, 306, 307, 308, 310, 311, 313, 314, 402, 403, 404, 405, 485]. These examples use global, patterned, or vectorial light to select actuation modes, while feedback or sequential illumination can convert simple bending into locomotion, gripping, or repeated mechanical cycles [68, 70, 302, 308, 313, 401, 402, 403, 404, 405, 406, 485]. The key design metrics differ from those of optical devices: actuation strain, blocking force, response speed, penetration depth, modulus, fatigue resistance, and control of heat accumulation determine performance [412, 418, 419, 483, 486, 487, 488]. Mechanical robustness and manufacturability are particularly important because devices must survive repeated stress cycles [299, 306, 307, 480, 489, 490].

A second mechanical route is directional mass transport and surface reconfiguration [28, 29, 171, 194, 213, 215, 220, 479, 491]. Figure 20c connects SRG formation, vectorial‐field‐guided deformation, and programmable topography: local polarization or structured light generates local stress, and VPA‐like stress pathways drive surface relief, anisotropic transport, or 3D microstructure reconfiguration [194, 200, 212, 215, 220, 222, 223, 224, 255]. This concept moves azopolymer deformation from empirical photofluidization toward model‐guided surface morphogenesis [206, 220, 222, 492]. Figure 20d extends light‐softened azobenzene materials to athermal NIL and multi‐nanopatterning. Azo‐NIL uses photoinduced softening to fill a stamp at room temperature without conventional thermal NIL processing, and PM6AZC4‐type polymers enable regionally selective multiple nanopatterns on flexible substrates [225, 226, 227].

Solid‐to‐soft and solid‐to‐liquid transitions further broaden processing functions. Figure 20e shows repairable coatings and self‐healing behavior, where light exposure closes scratches or increases chain mobility through photosoftening, *T_g_* switching, or solid‐to‐soft transition [54, 55, 337, 340, 343, 413, 415, 416, 417, 481, 482]. Figure 20f shows mechanically mutable materials and conformal processing: an ABA triblock copolymer with a photoresponsive azopolymer midblock and glassy polystyrene end blocks can switch at room temperature from a stiff plastic to a soft elastomer, enabling imprinting or packaging on curved, nonplanar, or skin‐like substrates [341]. Together, Figure 20 demonstrates that azobenzene mechanical functions are not limited to actuation; they include shape programming, surface morphogenesis, nanoimprinting, healing, and mechanically adaptive integration [12, 24, 226, 337, 340, 341, 479, 480, 481, 482, 493].

### 3.5 Bioadaptive Transport, Photopharmacology, and Synthetic‐Cell Systems

Bioadaptive azobenzene systems form a distinct application family because the desired output is transport, permeability, binding, or biological activity rather than diffraction or mechanical work. Figure 21a summarizes photopharmacology and molecular biocontrol, where azobenzene switching changes molecular geometry and thereby regulates receptor activation, ion‐channel conductance, antimicrobial activity, or cellular signaling [71, 92, 107, 425, 494, 495, 496, 497, 498, 499, 500]. The central design challenge is to operate at biocompatible wavelengths, in aqueous or membrane environments, with sufficient selectivity, fatigue resistance, and switching lifetime to match biological timescales [501, 502, 503, 504].

**FIGURE 21:**
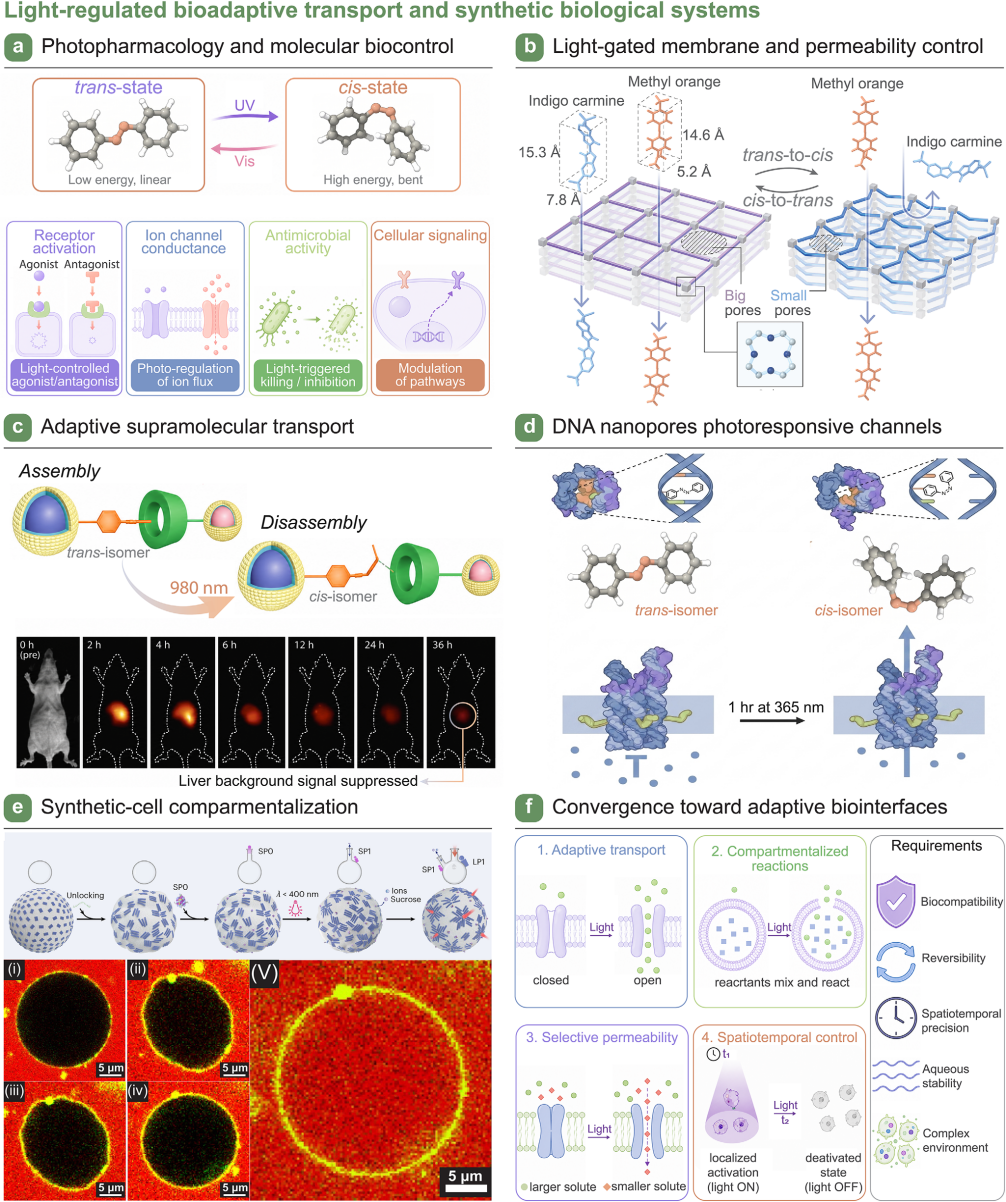
Photopharmacology and molecular biocontrol enabled by azobenzene photoisomerization. (a) Reversible *trans–cis* switching changes molecular geometry and polarity, enabling light‐regulated modulation of receptor activation, ion‐channel conductance, antimicrobial activity, and cellular signaling [71, 92, 107, 494, 495, 496, 497, 498, 504]. (b) Schematic illustration of light driven gating and release mechanisms enabled by azobenzene photoisomerization. Adapted with permission [72]. Copyright 2020, AAAS. (c) Reversible assembly and disassembly of supramolecular nanoprobes through *trans–cis* switching of azobenzene under 980 nm near‐IR (NIR) irradiation. NIR‐induced *trans–cis* photoisomerization triggers disassembly, accelerating probe clearance and suppressing background liver signals over time. Adapted with permission [505]. Copyright 2018, Wiley‐VCH Verlag GmbH & Co. KGaA, Weinheim. (d) Reversible light‐gated opening of membrane‐inserted light‐controlled nanopore modified with azobenzene moieties. Adapted under the terms of the Creative Commons Attribution 4.0 International License [506]. Copyright 2022, The Authors. Angewandte Chemie International Edition published by Wiley‐VCH GmbH. (e) Synthetic‐cell microreactors and compartmentalization based on interacting dynamic DNA‐based pores: azobenzene‐modified small pores regulate small‐molecule or ion transport, while DNA‐raft‐mediated large pores enable macromolecular exchange and compartmentalized biochemical reactions. Adapted under the terms of the Creative Commons Attribution 4.0 International License [437]. Copyright 2026, The Authors, published by Springer Nature. (f) Generalized framework of the key functional principles and design requirements underlying emerging synthetic‐cell and biohybrid platforms.

Azobenzene units incorporated into lipid membranes, polymer membranes, or nanoporous frameworks can reversibly switch between closed and open states, thereby modulating molecular transport and enabling light‐controlled cargo release under UV/vis/NIR irradiation [72, 421, 422, 423, 424]. Figure 21b highlights representative self‐standing covalent organic networks (CON), where azobenzene photoisomerization reversibly switches the pore aperture between open and closed states, thereby modulating molecular sieving and solvent permeance [72]. Azobenzene‐based host‐guest interactions provide a versatile platform for supramolecular transport and bioadaptive permeability control. Through reversible trans to *cis* photoisomerization, azobenzene containing assemblies can dynamically regulate intermolecular recognition, nanoscale assembly states, and membrane transport activity, enabling light‐controlled modulation of ion transport, permeability, and molecular release [507, 508]. As a representative example, Zhao et al. reported a near‐IR (NIR) responsive supramolecular imaging platform based on reversible host‐guest interactions between azobenzene‐functionalized upconversion nanoparticles and *β*‐cyclodextrin‐modified NIR‐II probes. NIR‐induced photoisomerization triggered reversible assembly/disassembly, enabling adaptive tumor retention and clearance through light‐controlled transport behavior (Figure 21c) [505]. These examples show how changes in molecular shape and polarity are amplified through membrane packing, pore‐size switching, and host‐guest binding [424, 509].

Recent DNA nanopores have progressed from solid‐state hybrid structures to lipid‐embedded and reconfigurable channels, enabling selective molecular sensing, tunable conductance, and controlled cargo exchange across synthetic membranes [510, 511, 512]. In Figure 21d [506], a light‐gated DNA nanopore exemplifies this concept: an azobenzene‐modified lid undergoes photoinduced duplex destabilization, opening the channel and activating small‐molecule transport across the membrane [513]. Figure 21e highlights the newly reported double‐necked synthetic cell microreactor containing two interacting dynamic DNA‐based pores [437]. The first pore is a light‐responsive small pore bearing an azobenzene‐modified lid strand: UV light (365 nm wavelength) opens the pore by converting *trans* to *cis*, whereas visible light (450 nm wavelength) recloses it. The second pore is a sealable large pore formed through DNA‐raft reconfiguration and giant unilamellar vesicle (GUV) membrane dynamics [514]. The interplay between these pores enables sequential import, compartmentalized reaction, and product export with high spatiotemporal control [437].

Azobenzene‐based bioadaptive systems have rapidly expanded across the broader biomedical and biointerface landscape, including drug delivery, light‐regulated transport, artificial organelles, biosensing, and synthetic‐cell communication [515, 516]. Across these platforms, molecular photoisomerization acts as a unifying transduction mechanism that converts nanoscale conformational changes into adaptive transport, compartmentalized reactions, selective permeability, and dynamic biointeractions [13, 423]. In contrast to conventional photonic or mechanically driven azobenzene systems, bioadaptive platforms must operate reliably within aqueous, crowded, and chemically complex biological environments while simultaneously maintaining biocompatibility, reversibility, and high spatiotemporal precision (Figure 21f) [78, 501, 502]. These emerging capabilities position azobenzene photoswitches as promising molecular building blocks for next‐generation synthetic‐cell systems, programmable therapeutic interfaces, and light‐regulated biohybrid materials [506, 510, 517].

### 3.6 Opto/Iontronic Adaptive Interfaces and Emerging Hybrid Devices

A final emerging application family uses azobenzene to modulate charge, ions, dipoles, interfacial potential, and mixed ionic‐electronic transport [426, 434, 438, 439, 518, 519]. Figure 22a shows the basic molecular mechanism: *E/Z* switching changes dipole moment, polarity, and interfacial orientation, which can alter surface potential, dielectric response, or local electrostatic environment [440]. Such polarity modulation is useful when the desired output is not an optical image but a conductance, charge mobility, resistance state, or electronic/ionic gating response [426, 434, 438, 518, 519, 520, 521, 522].

**FIGURE 22:**
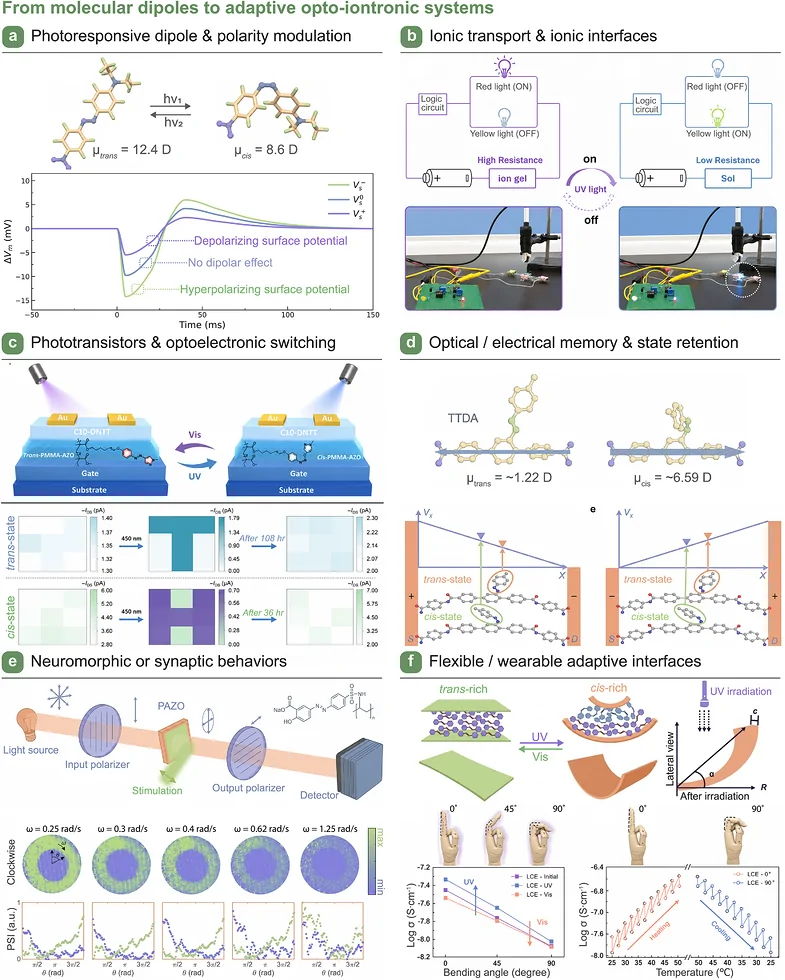
Opto/iontronic adaptive interfaces and electronic functions of azobenzene materials. (a) Dipolar and mechanical mechanisms jointly govern membrane photoresponses in azobenzene systems. Light‐induced dipole changes can either oppose or reinforce opto‐mechanical capacitance effects, thereby modulating the amplitude and polarity of membrane‐voltage responses. Adapted under the terms of the Creative Commons Attribution 4.0 International License [440]. Copyright 2025, The Authors, published by Springer. (b) Light‐responsive iontronic switching based on an azobenzene ion‐gel system. UV‐induced *trans*‐to‐*cis* photoisomerization changes the material from a high‐resistance ion‐gel state to a low‐resistance sol state, thereby reversibly modulating ionic conductance and logic‐circuit output. Adapted with permission [427]. Copyright 2020, American Chemical Society. (c) Azobenzene‐modulated phototransistors and optoelectronic switching devices based on PMMA‐AZO dielectric interfaces. Adapted under the terms of the Creative Commons Attribution 4.0 International License [434]. Copyright 2024, The Authors, published by AAAS. (d) Dipole projections of the *trans* and *cis* isomers along the molecular backbone and schematic bias‐dependent energy diagrams illustrate how the electric field shifts their relative stability in the single‐molecule junction. Adapted under the terms of the Creative Commons Attribution 4.0 International License [519]. Copyright 2019, The Authors, published by Springer Nature. (e) PAZO‐based neuromorphic optical platform for motion perception and spatiotemporal visual processing. Polarization‐dependent birefringence changes generate dynamic PSI responses that encode the speed and direction of moving optical stimuli. Adapted under the terms of the Creative Commons Attribution 4.0 International License [435]. Copyright 2025, The Authors, published by Wiley‐VCH GmbH. (f) Flexible and wearable adaptive interfaces based on azobenzene‐containing ionic LCEs. Photoisomerization and thermal stimulation reversibly modulate bending deformation and ionic conductivity, enabling smart ionic skins and adaptive soft robotic interfaces. Adapted with permission [426]. Copyright 2023, Wiley‐VCH GmbH.

Iontronic systems translate azobenzene photoisomerization into changes in ionic transport behavior. Upon light irradiation, the *trans*‐to‐*cis* transition modifies molecular polarity, packing density, and free volume, thereby altering ion mobility and conductive pathways within soft ionic media [426, 523]. Figure 22b highlights a representative light‐responsive iontronic device based on an azobenzene ion gel, where UV‐induced isomerization switches the material from a high‐resistance ion‐gel state to a low‐resistance sol state, enabling reversible optical control of ionic conductance and logic‐circuit operation [427]. Azobenzene‐based optoelectronic phototransistors operate through reversible photoisomerization at semiconductor/dielectric interfaces, where *trans–cis* switching dynamically alters dipole moment, interfacial polarity, charge trapping, and molecular packing. These interfacial changes modulate carrier transport and channel conductivity under optical stimulation, enabling light‐programmable switching, tunable photoresponse, and nonvolatile photomemory behavior [428, 431, 433, 434, 518]. Figure 22c illustrates this concept using an arylazopyrazole‐functionalized PMMA dielectric integrated with a C10‐DNTT (Dinaphtho[2,3‐b:2′,3′‐f]thieno[3,2‐b]thiophene) phototransistor platform. Upon alternating UV and visible‐light irradiation, the *trans‐* and *cis‐*PMMA‐AZO states produce distinct transport environments, leading to large differences in carrier mobility, photocurrent, and retention time. The device further demonstrates reversible optical image sensing and long‐lived photomemory behavior with retention extending up to 108 h in the *trans* state [434]. Optical/electrical molecular memory systems utilize bistable *trans*/*cis* azobenzene states to generate reversible high‐ and low‐conductance states with retention and rewritability [519, 520, 521, 522]. Photo‐ or electric‐field‐induced isomerization modifies dipole alignment and charge transport, enabling nonvolatile molecular memory operation. Figure 22d illustrates this concept using a graphene–molecule–graphene single‐molecule junction containing a TTDA (2′‐(*p*‐Tolyldiazenyl)‐[1,1′:4′,1″‐terphenyl]‐4,4″‐dicarboxylic acid) azobenzene side‐group gate. The distinct dipole moments of the *trans* and *cis* states produce different energy landscapes and conductance states, enabling reversible optical/electrical switching and rewritable molecular memory behavior [519]. Neuromorphic and synaptic azobenzene systems emulate biological information processing by converting optical stimuli into adaptive and history‐dependent responses [431, 432, 433, 435]. Figure 22e illustrates this concept using a PAZO azopolymer platform operating within a cross‐polarized optical architecture. Polarized light stimulation induces reversible birefringence and molecular alignment in the PAZO film, generating tunable post‐synaptic intensity (PSI) signals that depend on stimulation history and temporal correlation [435]. Azobenzene‐based flexible and wearable adaptive interfaces combine molecular photoisomerization with soft ionic and mechanical functionality. Reversible *trans–cis* switching dynamically changes molecular packing, polarity, and liquid‐crystalline order, enabling light‐controlled actuation, ionic conductivity switching, and mechanoresponsive sensing. Recent azobenzene‐integrated LCEs further demonstrate wearable ionic skins and soft robotic platforms capable of adaptive deformation, strain sensing, and multimodal environmental responsiveness [426, 524]. Figure 22f highlights flexible and wearable adaptive interfaces based on azobenzene‐containing ionic LCEs, where photoisomerization and thermally induced molecular reorganization dynamically modulate both mechanical deformation and ionic conductivity. UV/vis irradiation and thermal stimulation reversibly control bending actuation, molecular alignment, and ion‐transport pathways, enabling multimodal strain sensing and adaptive ionic switching. These properties further enable wearable ionic skins and soft robotic interfaces capable of programmable motion, environmental responsiveness, and real‐time mechanical signal detection [426].

Across all application families, the design lesson is that azobenzene is not a universal switch with one optimal molecular structure. Its usefulness depends on matching molecular switching metrics to the desired output. The same *E/Z* photoswitching event can lead to alignment, packing, stress/strain, permeability, heat release, or ionic/electronic modulation, but each application family imposes a different trade‐off: optical loss versus diffraction efficiency, response time versus cycling stability, energy density versus lifetime, actuation strain versus modulus, bioselectivity versus reversibility, and state retention versus operating voltage [1, 12, 71, 76, 289, 518, 525, 526, 527]. This function‐first framework therefore provides a unified criterion for section 3 and clarifies how azobenzene molecular design, mesoscopic material response, and adaptive device function should be co‐optimized.

## 4 Conclusion and Outlook

This review has used azobenzene to illustrate a cross‐scale design principle: reversible molecular *E/Z* switching can be amplified into optical anisotropy, stress generation, mass transport, phase transitions, macroscopic deformation, heat release, and device‐level photonic functions [5, 12, 24, 30, 287, 432]. The field is mature in its molecular foundations, yet many of the most important questions are now multiscale: how can molecular excitation and orientation be quantitatively connected to stress, flow, deformation, optical phase, and device reliability under realistic operating conditions?

Several challenges recur across the application families discussed above. First, visible‐, red‐, and NIR activation remain essential for biological, thick‐film, and solar applications, but red‐shifting often competes with high PSS conversion, high quantum yield, and long *Z*‐state lifetime [77, 494, 528, 529, 530, 531, 532]. Second, fatigue resistance and photobleaching must be improved under high‐intensity or long‐cycle operation [1, 2, 296, 297, 298, 305]. Third, limited penetration depth and local heating complicate the translation from thin films to bulk materials and devices [296, 297, 298]. Fourth, predictive models must connect molecular structure, packing, orientation dynamics, light‐induced stress, viscoplasticity [223], LC elasticity [318, 319, 320], and device geometry. Finally, scalable manufacturing, mechanical robustness, and integration with photonic, energy, and biomedical platforms remain decisive for practical deployment [57, 71, 77].

Figure 23 summarizes these bottlenecks and the corresponding opportunities. At the molecular level, red/NIR photoswitches, triplet sensitization, two‐photon excitation, upconversion, and host‐guest confinement can expand the usable spectral window [533, 534]. At the discovery level, machine‐learning and high‐throughput computation can help identify structures that balance absorption wavelength, PSS, half‐life, quantum yield, and fatigue resistance [156, 535, 536]. At the materials level, multiscale theory and experiments should link orientation‐stress‐deformation with hierarchical architectures, chiral assemblies, LC elasticity, and polymer mechanics [319, 537, 538]. At the manufacturing level, printable azopolymers, reusable molds, additive manufacturing, and large‐area photopatterning can make adaptive photonic components more accessible [205, 262, 539]. At the systems level, integration with metasurfaces [65], polaritonic dispersion and band‐structure engineering [442], MOST modules [540], soft robotics, and bioadaptive interfaces could move azobenzene materials from responsive films toward multifunctional adaptive matter.

**FIGURE 23:**
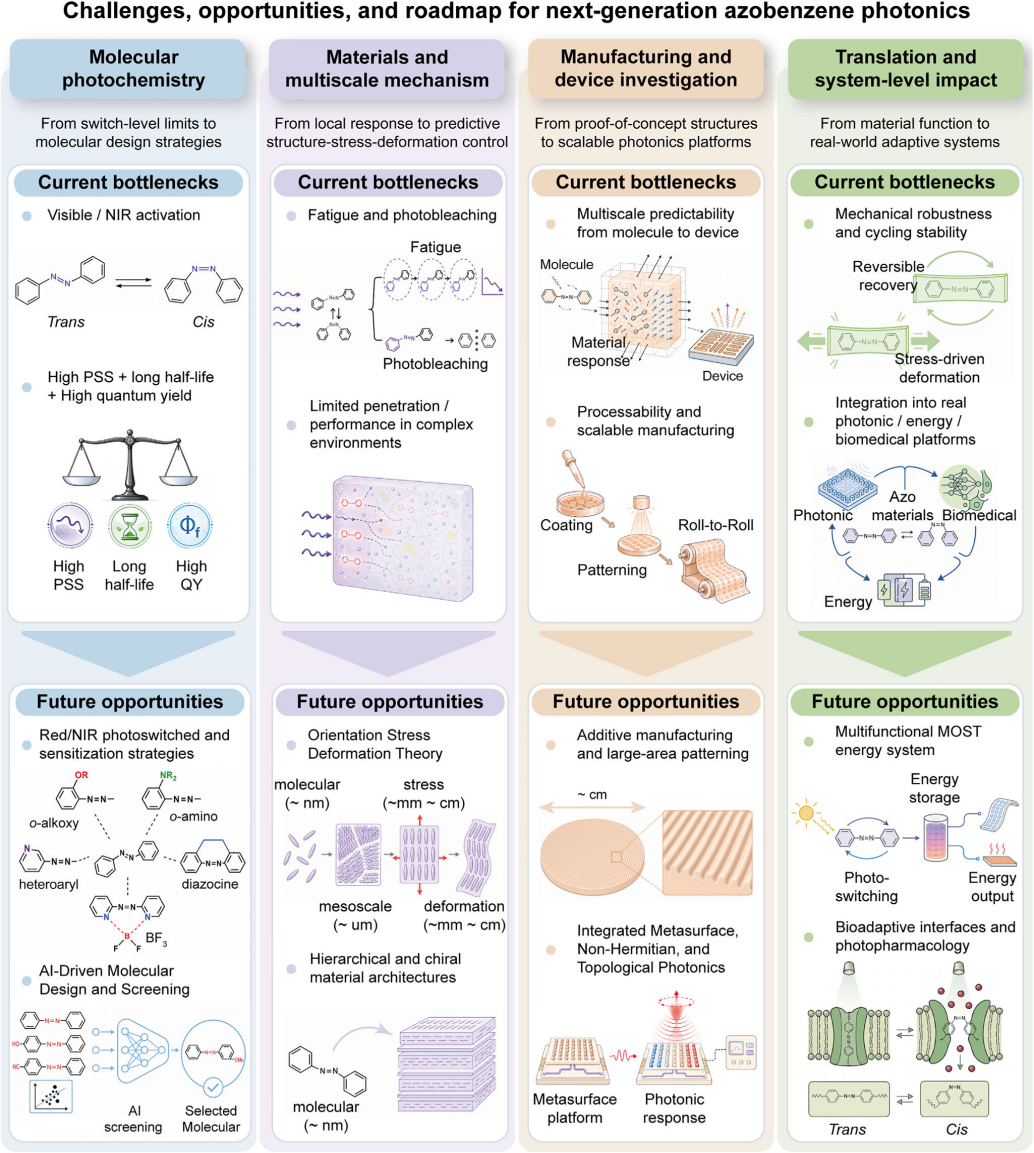
Challenges, opportunities, and roadmap for next‐generation azobenzene photonics, organized into four domains (molecular photochemistry, materials and multiscale mechanism, manufacturing and device investigation, and translation and system‐level impact) that map current bottlenecks (top) onto future opportunities (bottom). Current bottlenecks include visible/NIR activation, simultaneous optimization of PSS, half‐life and quantum yield, fatigue resistance, penetration depth, multiscale predictability, scalable manufacturing, mechanical robustness, and device integration. Future opportunities include red/NIR photoswitches and sensitization strategies, AI‐guided molecular design, multiscale theory, hierarchical and chiral materials, large‐area patterning, integrated photonics, MOST‐coupled multifunctional systems, and bioadaptive interfaces.

The most promising future directions will likely combine functions that have often been studied separately. For example, a material could couple MOST heat release to shape change or refractive‐index modulation; a photonic surface could store optical information while also acting as a switchable mechanical or thermal interface; and a LC architecture could combine photoalignment, chiral optical response, and active wavefront control [525, 526, 527]. Such multifunctionality will require design strategies that treat molecular switching metrics, collective material response, and device architecture as a single optimization problem rather than as independent stages.

In this context, azobenzene remains valuable not because it solves every application challenge, but because it provides a chemically versatile and experimentally accessible model system for studying how molecular motion becomes macroscopic function [12]. Continued progress will depend on coordinated advances in synthesis, spectroscopy, theory, mechanics, manufacturing, and device engineering [1, 5, 12, 541]. A realistic goal for the next stage is visible‐light‐addressable, durable, scalable, and multifunctional azobenzene‐based adaptive photonic matter that can operate reliably in practical optical, energy, robotic, and biomedical environments.

## Conflicts of Interest

The authors declare no conflicts of interest.

## Data Availability

The authors have nothing to report.
